# The Overlooked Tradition of “Personal Music” and Its Place in the Evolution of Music

**DOI:** 10.3389/fpsyg.2019.03051

**Published:** 2020-02-18

**Authors:** Aleksey Nikolsky, Eduard Alekseyev, Ivan Alekseev, Varvara Dyakonova

**Affiliations:** ^1^Braavo Enterprises, Los Angeles, CA, United States; ^2^Independent Researcher, Boston, MA, United States; ^3^The State Institute for Art Studies of the Ministry of Culture of the Russian Federation, Moscow, Russia; ^4^Experimental Laboratory of the North-Eastern Federal University, Yakutsk, Russia; ^5^International Jaw Harp Music Center, Yakutsk, Russia; ^6^Department of Art Studies, Arctic State Institute of Arts and Culture, Yakutsk, Russia

**Keywords:** timbre-based music, personal song, Jaw harp (aka Jew's harp), musicality, arctic hysteria, music evolution, thematicism, musical texture

## Abstract

This is an attempt to describe and explain so-called timbre-based music as a special system of musicking, communication, and psychological and social usage, which along with its corresponding beliefs constitutes a viable alternative to “frequency-based” music. Unfortunately, the current scientific research into music has been skewed almost entirely in favor of the frequency-based music prevalent in the West. Subsequently, whenever samples of timbre-based music attract the attention of Western researchers, these are usually interpreted as “defective” implementations of frequency-based music. The presence of discrete pitch is often regarded as the structural criterion that distinguishes music from non-music. We would like to present evidence to the contrary—in support of the existence of indigenous music systems based on the discretization and patterning of aspects of timbre, rather than pitch. This evidence comes mainly from extensive ethnographic research systematically conducted in eastern European and Asian parts of Russia from the 1890s. It involved the efforts of thousands of specialists and was coordinated by dozens of research institutions, and it has included not just ethnomusicology but linguistics, philology, organology, archaeology, anthropology, geography, and religious, and social studies. Much of the data has not been translated into Western languages. Although some Soviet-era publications were tainted by Marxist ideology, many researchers strove to provide accurate information (despite at times having been prosecuted for their work), and post-1990 research undertook a substantial revision of ideologically compromised concepts. Timbre-based tonal organization (TO) differs from that based on frequency in its personal orientation: musicking here occurs primarily for oneself and/or for close relatives/friends. Collective music-making is rare and exceptional. The foundation of timbre-based music seems to have vocal roots and rests on “personal song”—a system of personal identification through individualized patterns of rhythm, timbre, and pitch contour, utilized like a “human voice”—whose sound enables the recognition of a particular individual. The instrumental counterpart of the personalized singing tradition is the jaw harp tradition. The jaw harp is the principal musical instrument for at least 21 ethnicities in Russia, who occupy over half the territory of the country. The evolution of its TO forms the backbone for the development of timbre-based music art. Here, we provide the acoustic, socio-cultural, geographic, and chronological overview of timbre-based music.

In the past few decades, a lively discussion on matters concerning the origin and evolution of music has finally begun to move toward a consensus among specialists (Cross and Morley, 2009): the biological importance of music is being seen in its capacity to foster and sustain social interactions within a group, to the mutual benefit of its members. Here, music stands as an important counterpart to language—another biological marker of *Homo sapiens*—specializing in managing the emotional aspect of human interaction. Without diminishing the importance of this perspective, we wish to cast light as well on the *personal* function of music—its capacity to organize and sustain the psychological identity of an individual. This function must have factored in the evolution of music since at least the Upper Paleolithic.

The need for the update grows as more scholars lean toward regarding music as an exclusively collective phenomenon. Thus, Lewis (2013) concludes: “In most parts of the world, and *for most of human history*, music exists *only* because of the *social relations that enable its performance*.” Levinson (2013) infers that in the evolution of music its structural complexity “may have its origins in *joint action* rather than in *abstract representations* or *solitary mentation*”^1^. We will argue the contrary—that at least in the traditional lifestyle of numerous native Siberian ethnoses, music serves *primarily* as means of solitary mentation and abstract representation of reality. There is no reason to believe that this manner of “musicking” (Small, 1998) is a recent development, and there are valid reasons to prototype prehistoric North Eurasian music upon this type of music.

## Dichotomy Between Timbre-Based and Frequency-Based Music

Almost everything that we know about the perception of music and what has served as a scientifically established foundation for modern views on the origin of music comes from a musical tradition based on frequency discrimination of musical sounds. It is this tradition that currently prevails in the world. Its prevalence probably started with the rise of Bronze Age urban civilizations, whose palace and temple music traditions relied on math-based theory (Nikolsky, 2016). Rationally defined pitches have made the corresponding music practices rely on the frequency aspect. Civilizations that cultivated frequency-based music imposed their influence on the music cultures of neighboring peoples. On a global scale, this must have resulted in a steady decline of the alternative form of TO that was based on timbre. This process is evident in the music cultures of many native Siberian peoples, e.g., the Nganasan, whose timbre-based tradition has been recently overtaken by the Russian frequency-based tradition (Bicheool, 2009). Schneider (2001) qualifies such development as “pitch reductionism”—the replacement of timbre-based tuning standards by frequency-based standards, most obvious in the indigenous gong/bell and xylophone music. Timbral vocal music is no less vulnerable to pitch reductionism.

**Examples-1/2**^2^

Ex.1. Timbre-based vocal music: throat-singing song, “Seagull,” in archaic style, by Anna Ankhani, a 70-year-old Koryak woman from a remote reindeer-breeding settlement, Khailino, in Northern Kamchatka (reachable only by aerial transportation). Singing involves throat-rasping and double phonation that allows the singer to produce sounds an octave below her speaking voice range (http://chirb.it/n5JNvk).

Ex.2. Modern style Koryak “Festive song” by Maria Appolon, a 48-year-old woman from a little town, Ossora, a seaport at Bering Sea. Maria is a legislator in the local district Council and a member of the folk ensemble, “Agya,” that often performs at international festivals, special events, and for tourists. Her singing exhibits traits of “frequency-based music”: no timbral effects, clear discrete pitches that are interconnected without gliding that is so typical for traditional singing in Kamchatka, hexatonic mode, consisting of 2 motifs (a descending trichord and an ascending tetrachord) which retain amazing precision in intonation, without noticeable fluctuations, and strict formulaic structure (http://chirb.it/FK7A8A).

In the literature in English, the distinction between “pitch-centered” and “timbre-centered” musics was drawn by Levin and Suzukei (2006, p. 45–72). Fales (2002) discussed the downsides of “pitch-centrism” and “timbre-deafness” in approaching timbre-based music. In the Russian literature, the dialectics of timbre and pitch was acknowledged much earlier. The pioneer of ear-training and music psychology in Russia, Maykapar (1900), noticed that the musical ear developed from a timbre- to a frequency-reference frame, and proposed to begin musical education with timbral exercises. Since then, ear-training has become an obligatory part of music education in Russia, and has attracted many researchers (Blium, 1977). Adoption of a state-controlled, unified system of early music education by the Bolsheviks created an exceptionally diversified cross-cultural pool of subjects for the investigation of music skills acquisition (Arakelova, 2012). Based on such data, Teplov (1947)^3^ formulated his theory of interference between “timbral” (speech-like) and “pitch” (music-like) hearing—the latter typically replacing the former by the beginning of a child's primary education, provided there was cultural exposure to frequency-based music^4^.

Teplov's theory was substantiated by Leontyev (2009, p. 115–36), Vygotsky's pupil, in a series of experiments that disclosed a “timbre-centered” estimation of pitch in adults who lacked music schooling^5^. To demonstrate that pitch-ear is a “functional organ”^6^—absent at the moment of birth and forming as a consequence of cognitive development—Leontyev built a vibrator machine and successfully taught subjects to discriminate pitch exclusively by touch. Another Vygotskian, Zaporozhets, headed the research on the emergence of frequency-oriented hearing in preschool children (Endovitskaya, 1959, 1964; Lisina, 1966; Mukhina and Lisina, 1966; Repina, 1966a,b; Zaporozhets, 2003)^7^. His group concluded that the genesis of frequency discrimination reflected a fundamental re-organization of motoric, visual, and spatial orientations throughout childhood—embodying a form of abstraction “of a number of perceptual activities, directed at the exploration of objects and phenomena of reality, identification and fixation of their perceptual attributes and interrelations” (Zaporozhets, 1985, p. 1:18).

The frequency-based “musical ear” corresponds to the urban Western environment, where systemically organized straight parallel lines and right angles are widespread and dominant. But these do not exist in the steppe or tundra (Nikolsky, 2016, Appendix-7 in Supplementary Material). Life in the tundra promotes music systems based on *indefinite (ekmelic or khasmatonal) pitch* (Alekseyev, 1986) and *fine timbral distinctions*, corresponding to life in an open terrain that lacks landmarks. There, orientation occurs via *qualitative* (non-quantitative!) evaluations of wind, light, snow, distance, etc., rather than the incremental and reversible pathfinding in the urban landscape. Most likely, timbre-centrism originates from life in such natural sound-stages where “definite” rhythm and frequency are mostly bound to human activities, and are overall less important than timbre-differentiated environmental sounds (Fales, 2002). The opposition of indefinite/definite pitch systems in Russian musicology finds its match in the opposition of “smooth” and “striated” pitch in Western ethnomusicology (During and During, 2015).

Timbre-centeredness distinguishes the vocalizations of newborns: crying, screaming, rasping, grunting, whining, sobbing, whimpering, etc. (Loewy, 1995). Even prenatally, fetuses recognize parental voices (Lee and Kisilevsky, 2014), which involves at least some timbral discrimination. Newborns distinguish different musical instruments by timbre (McAdams and Bertoncini, 1997). Timbre discrimination is so crucial for a newborn's life that it appears innate (Simons, 1986, p. 43). Perhaps the ontogenetic progression from timbre to frequency (Teplov, 1947) takes the same course as cultural evolution, and “indefinite-pitch” music systems precede “definite-pitch” systems (Alekseyev, 1986, p. 14–15)—following the general paradigm suggested by Foster (1994). Unfortunately, published research on the acquisition of musical skills overwhelmingly focuses on the experience of children in Western societies^8^. Garfias (1990) seems to share Alekseyev's conviction that in indigenous music cultures infants start their musical development from speech-like timbral hearing. Tuvan children learn early to vocally imitate typical environmental sounds with amazing precision, adopting learned timbral distinctions for the creation of their own music (Levin and Suzukei, 2006, p. 85–7)—very much like Western children model their vocal improvisations upon commonly heard tunes (Bjørkvold, 1992). Although Tuvans also use frequency-based music, it remains secondary in importance for them.

In Western classical music, the opposite pertains: it is timbre that is secondary (Scruton, 1997, p. 77–8). Even its standard definition—the “attribute of sensation in terms of which a listener can judge that two sounds having the same loudness and pitch are dissimilar” (ASA, 1951)—is culturally skewed toward prioritizing frequency. Western “frequency-centrism” is most evident in the tendency to ascribe pitch values to clearly non-musical sounds (e.g., car brakes)—including sounds of non-Western timbral music, such as Inuit (Walker, 1997, p. 323). Unsurprisingly, Westerners' attempts to reproduce indigenous timbral music introduce distortions and are rejected by native users (Ojamaa, 2005).

The reverse bias affects the use of frequency-centered music by timbre-centered musicians. Soviet researchers of the collectivization/*likbez* era discovered that children (Beliayeva-Ekzempliarskaya, 1925) and adolescents (Antoshina, 1939) who lacked exposure to classical music could not detect a harmonic mismatch when a well-known melody was performed against accompaniment in a wrong key. Transference of popular tunes across different music systems usually involves systemic pitch conversion. Thus, the Russian-Ukrainian diatonic heptatonic song “Provody” becomes anhemitonic pentatonic in Buryat and whole-tone tetratonic in Yakut reproductions, severely distorting the original intervallic structure (Alekseyev, 1986, p. 148–55). Both, Buryats and Yakuts, cultivate timbral music. It might be appropriate here to speak of the intuitive substitution of “pitch classes” of the original foreign song (which was composed circa 1918 in Ukraine within the framework of Western tonality) by the “timbral classes” of traditional Buryat and Yakut music which sounded the closest to the original. The “strange” and complex sounding tonality of the foreign song, which for some reason attracted the attention of the indigenous musicians, was substituted by the TO that was habitually “normal” and easy for them.

Timbre-oriented musical cultures seem to base their musical modes on a set of pitch levels that are joined together according to some common trait(s) of timbral coloration and/or sound production technique(s). Thereby a number of such related “timbre-classes” become united into a “timbral mode.” This is in contrast to a specific intervallic distance in pitch that distinguishes one “degree” of a musical mode and/or key from another “degree” in frequency-oriented music cultures. Inevitable conversion from pitch- to timbre-based frame of reference must be responsible for the intervallic distortions, discovered by Alekseyev in a Russian-Ukranian tonal song upon its appropriation by the indigenous Buryat and Yakut musicians.

## Personal Nature of Timbre-Based Music

Often overlooked is the fundamental unsuitability of timbre-centered music for collective use—in sharp contrast to frequency-centered music. Even before the crystallization of Western tonality, musicians realized that the more instruments that played the same harmony, the more euphonic it appeared (Mersenne, 1957, p. 270). However, the more speakers who collectively pronounce the same sentence, the less articulate it sounds. People sing together, but take turns speaking (Brown, 2007). Fusion of sounds dominates production and perception of music (Huron, 2001) because pitches readily conjoin in unisons, “double-notes” and chords. Timbre-centered music, however, resembles speech in its soliloquacity: for a native listener, simultaneous throat-singing by a dozen singers would make the song unintelligible.

Unlike the simple dimensionality of height for pitch, the multi-dimensionality of timbre considerably complicates its categorization (Krumhansl, 1989). Timbre-based music also misses the systemic rationality of frequency-based music systems. The interrelation of pitches cross-refers any given pitch value, facilitating the interpretation of pitch by making it “rational”—whereas timbre stands as “irrational,” lacking anything akin to intervallic ratios (Balzano, 1986). To add to the confusion, the temporal dimensions of timbre interact with its spectral dimensions (Caclin et al., 2007), disallowing multiple timbres to synchronize perfectly. Different timbres, dubbed in unison, either blend into a new timbre, intensifying one of the constituents, or repel each other (Sandell, 1995). Blending depends primarily on the onset of synchrony, similarity of attacks, and/or spectral centroids—traditionally studied within the field of orchestration (Tardieu and McAdams, 2012). Orchestration is limited to frequency-centered music, where musical instruments are perfected to produce clear pitch. Even many popular instruments exhibit registrally fixed formants, and can therefore be considered “pitch-generalized”—which greatly influences their timbral blend (Lembke and McAdams, 2015). Unsurprisingly, unison blending is greater than non-unison blending, inverse to the identifiability of constituent timbres (Kendall and Carterette, 1993). The fusion of multiple “pitch-generalized” instruments generates orchestral *tutti*—an undistinguishable mass of timbres that nevertheless remains “pitch-clear.”

*There is no tutti in indigenous timbre-centered music*^9^. Such music cultivates very different instruments (whip, buzzer, cane, flask), distinguished by a characteristic timbre and indefinite pitch (Mazepus and Galitskaya, 1997). In Russian musicology, these are qualified as “phono-instruments”^10^—sound-producing tools manufactured for some common application other than music-making (Yesipova, 2008). The most important of them is the Jaw Harp (JH)^11^. In the contiguous area from the Urals to the Okhotsk Sea, all indigenous ethnicities have their JH traditions (Emsheimer, 1986). A century ago, every ethnicity of Asiatic Russia possessed the JH (Jochelson, 1928, p. 217). The JH can be qualified as the archetypical instrument of Siberia (Sheikin, 1996). It and other “mouth-resonating instruments” (mirlitons, bows, “singing-pipes”) *specialize in making “music for oneself”* (Sheikin, 2002, p. 116–66). The JH's solitary use prevails over the Volga Plateau (Zagretdinov, 1997), Tuva/Altai (Suzukei, 1989), Afghanistan (Koskoff, 2008, p. 1062), Sakhalin (Mamcheva, 2005), Taiwan (Blench, 2004), Indochina (Sam, 2008), Indonesia (Matusky, 2008), and New Guinea (Pugh-Kitingan, 1977).

Such a wide geographic distribution indicates the inter-cultural—*acoustic*—reason for JH's personal application: except for the recently invented JH models^12^, traditional instruments are barely audible beyond a few meters. Even in social settings, JH remains **private**. Such are JH's romantic responsorial duets between a young man and a young woman, known, other than in Russia, in Southern China (Picken, 1957, p. 154), Tibet (Arcones, 2013, p. 216), Hainan (Hsu, 2001), Taiwan (McGovern, 1922), Laos (Simana and Preisig, 2003), Vietnam (Rault and Brenton, 2000, p. 83), Indonesia (Kartomi, 2012, p. 160), New Guinea (Pugh-Kitingan, 1977), and Western Europe (Kolltveit, 2006, p. 111).

**Example-3**

“Serenading” *khomus* (Yakut JH): a romantic duet in traditional style, performed by Erkin Alekseyev and Tokuiaana Nikolayeva. Music proceeds in a responsorial setting, but ends with simultaneous playing that reflects the union of feelings (http://chirb.it/rH6bFD).

Germane in this context is that “serenading” duets involve taking turns, like interlocution: one player reproduces the sonic material suggested by another player (Haid, 1999).

The JH is also a child's favorite plaything—another cross-cultural sphere of private use—in Yakutia (Dyakonova, 2017), Ural (Aleksandrova, 2017), Sakhalin (Mamcheva, 2012, p. 197), Uzbekistan (Beliayev, 1933), Kyrgyzstan (Vinogradov, 1958, p. 180), Afghanistan (Slobin, 1976, p. 53), Mongolia (Pegg, 2001), Japan (Ishi, 1916), southeastern China (Picken, 1957), Polynesia (McLean, 1999), Indonesia (McPhee, 1955), and Western Europe (Kolltveit, 2006, p. 109). Toying with a JH is typical during the long hours of herding (Shchurov, 1995), a task entrusted to children in nomadic Asiatic societies (Stépanoff et al., 2017). In Altai, the JH is regarded as a herding instrument (Dorina, 2004). Children start working in pastures at ages 5–6 (Yekeyeva, 2011). In Yakutia, mothers teach their children to make JHs from tree splinters (Tchakhov, 2012). Nivkh 5-year-olds learn to play and make bamboo and grass JHs, guided by parents, grandparents, or older siblings^13^.

Self-manufacturing is an important marker of timbral instruments; personal use accompanies personal manufacturing (Dorina, 2004)^14^. Even metallic JHs are often self-made, such as by flattening brass rifle cartridge cases (Mamcheva, 2012, p. 50). Forging of metallic JHs by a metalsmith is a relatively recent historic development, but instruments made that way are no less personal. Even in modern Western societies, JH gatherings promote private musicking—mostly solo playing, without electronic amplification, and submerging into a meditative state (Morgan, 2017). For Siberian vocal and instrumental traditions such musicking constitutes the norm (Zabolotskaya, 2009).

Despite the JH's tuning to a certain fundamental frequency (**FF**), indigenous techniques obstruct standard Western notation (frequency-based), since the production of discrete pitches constitutes only a fraction of the possibilities (Alekseyev, 1991b).

**Example-4**

“Mary had a little lamb,” produced on khomus by Erkin Alekseyev. This music demonstrates inauthentic, Western-style treatment of JH as a “frequency-based” musical instrument whose purpose is to accurately reproduce the pitches of “tunes” (http://chirb.it/HNcBsq).

The bulk of the JH's autochthonous repertory consists of speech-like articulations and special sound effects (Alekseev, 1988). Common among players, the verbal characteristics of JHs' sounds are all timbre-oriented (Zagretdinov, 1997). Each timbre-distinguished device usually carries a particular semantic value (Shishigin, 1995)^15^.

The JH *tutti* would mess up the clarity of articulation, preventing the parsing of meaningful musical elements such as *tabygyr* (staccato) (Alekseyev, 1986). Therefore, autochthonous styles are strictly soliloquy-based. A good example is the Yakut *syyia tardy*^16^ (“moderate playing”), nicknamed “talking *khomus*”^17^ (Grigoryan, 1957), which is closely related to the vocal *toyuk* (Shishigin, 1995). *Toyuk* (song) is the most ancient form of *dieretii yrya*—a smooth-flowing, drawn out improvisational singing style, based on a limited set of melodic intonations, and timbrally individualized (Alekseyev, 2016). *Syyia*'s closeness to *toyuk* enables the simultaneous singing and playing of a JH.

**Example-5**

“*Tuluktan doborum*” (“Friendly bullfinch”)—spontaneously improvised simultaneous singing and playing on khomus by Agrafena Ptitsyna from Megino-Kangalasskii district of Yakutia. The performer starts in the style of degeren (rhythm-oriented, metrically regular), but after 30 s switches to another style, *dieretii* (smooth, metrically free style), and thereafter keeps alternating between the two. Instrumental and vocal parts interfere with each other: khomus as though obstructs attempts of the voice to carry out a coherent song (http://chirb.it/2D3pJh).

*Yakuts do not consider the khomus to be frequency-based*. They evaluate its sound not as “pitch,” but as “coloration.” Therefore, concurrent singing-playing constitutes “coloring” the same song. All Yakut music is predominantly solo-vocal, where “vocal” is understood as “mouth-driven”—incorporating both vocals and JHs (Alekseyev, 1991a).

The inclusion of the JH in bands is uncommon in indigenous traditions. It is bound to the South: i.e., India and Indonesia (Morgan, 2008). In northern Eurasia, JH playing is usually solitary, and an additional player can participate only if he/she is a close relative^18^. The JH “combo” is a recent phenomenon^19^, inspired by Western chamber music (Alekseyev, 1988, p. 185–7)^20^. Still, Siberian ensembles resemble the jazz combo by limiting *tutti* to only the start/finish of the music. Superimposition of JHs is understood here as a timbral mixture rather than as “frequency intervals”—unlike the counterpoint-style mentality of Austrian and German performers who group up to five or six JHs to produce “chords.”

## Transmission and Participation in Performance of Timbre-Based Music

Frequency- and timbre-based musics fundamentally differ from one another with respect to transmission. It is much harder to reproduce someone's timbre than their pitch. Timbre is usually personalized. We recognize a person's voice and a musical instrument by its timbre. Consequently, timbre-based music is designed to reflect the state of a unique individual rather than that of a group. Timbre (Saitis and Weinzierl, 2019) and sonority (McAdams, 2019) have been inherently connected to the display of emotions—even within the Western classical music tradition (Maddox, 2009).

- **Frequency-centered music** *attunes the individual to the collective* for their mutual benefit, enmeshing and empowering each participant through integration^21^;
- **Timbre-centered music** attunes the individual to the surrounding nature, supernatural forces, a soulmate, or “oneself-in-the-past”^22^—all of which also integrate but do not enmesh.

The inherent individualism of timbre-centered music originates from mother-infant vocal communication. Their vocalizations imitate each other in pitch-contour and timbre to establish a personal bond (Malloch, 2000). These imitation games are deeply private, defining conventions of a “**mini-culture**” for each caretaker-infant pair (Trevarthen, 2008). Such a mini-culture opposes “culture” by circumnavigating social conventions for the sake of securing effective communication with any child, no matter how anomalous he/she may be.

Participation in a group always imposes obligations, and group music makes no exception. However, timbral music performance is fundamentally “free.” The performer plays to his/her own satisfaction rather than the satisfaction of others. This is also true with a mini-culture: the caretaker and infant lock in a sympathetic communication that preserves individual freedom.

For timbral music cultures, infant **mini-cultures** develop into an adolescent “**maxi-culture**”—a maximally inclusive culture that allows everyone to remain himself/herself, rewarding them with a positive experience.

Timbral music acts like self-directed speech in problem-solving situations: it encourages behavioral reorganization and compensates for negative experience. And here the JH exemplifies timbral music. Notably, in many indigenous Eurasian cultures the JH is renowned for being a *female and children's* instrument (Mazepus and Galitskaya, 1997). The JH creates a mini-culture for each player within the ethnic maxi-culture.

## Personal Song (PS) as a Form of Timbre-Based Vocal Music

A peculiar form of “timbre-based” music is “**personal song**”^23^ (**PS**)—a traditional custom of assigning a specific “tune” to each individual that represents his/her identity and satisfies the required sonic uniqueness: i.e., it does not resemble the timbre and melody of another person's singing (Novik, 1999). A “personal tune” incorporates a particular melodic contour and timbre, and is used to coin multiple variations that the singer spontaneously forges while singing/humming during everyday activities (Novik, 2003). Most of one's day is accompanied by such semi-conscious singing. A comparison of different versions of one's PS recorded on different occasions shows great variability in text and emotional states along with stability in the melodic structure (Ojamaa and Ross, 2004), suggesting a link between “self” and melody. Furthermore, when a person is absent and missed by relatives, they will sing that person's PS as a substitute for his/her presence (Dobzhanskaya, 2017). The unauthorized performance of a PS by strangers is prohibited, and is punishable by the offended party (Grachiova, 1983, p. 56)^24^.

Parents create PSs for their children as though “sound-painting,” employing acoustic parameters to express the visual-motoric traits of the child (hyperactive/calm, headstrong/social, big/small).

**Examples-6/7**

Ex.6. Dinimiaku's children personal song, created and directed to Dinimiaku by her father, Tubiaku Kosterkin, from the settlement Ust'-Avam in Taimyr, in Nganasan. The song uses the timbral markers: contrasting “clean” high and “dirty” low registers connected together by sliding, and embellished with the “tremolo” effect. According to the performer, the song expresses tenderness and playful teasing. The lyrics question if the girl is upset at the old grouchy woman (Dobzhanskaya, 2014, p. 159) (http://chirb.it/7htOzK).

Ex.7. Derkuptie's children personal song, performed as if “coming from him” to the house guests, by his mother, Valentina Kosterkina, in Nganasan. The song is “infantilized”: raised in pitch and “whiny”—it emphasizes the descending sliding intonations, as though gently “complaining.” The lyrics imply that the infant-boy is overwhelmed by the visitors' attention and wishes to be left alone (Dobzhanskaya, 2014, p. 156) (http://chirb.it/zsxAEm).

After reaching adulthood, one might create or accept as a gift a second PS. Such renewal addresses the discrepancies between “infantile” and grownup states. The new PS often emulates the PS of a relative who is considered a role model^25^.

**Example-8**

Adult personal song of Ver'a Nenyang, performed by his daughter, Liubov' Nenyang. Nenets masculine personal songs are characterized by praising one's own strength, luck, and smartness (Nenyang, 2006). Nenets adult personal songs usually oppose two contrasting vocal registers, joined by strong portamento, use of few “degrees” (3 in this example), and exaggerated intonations - the melodic intervals between the degrees are stretchable within the range of about 700 cents (Dobzhanskaya, 2017) (http://chirb.it/dgenGN).

The third^26^ PS is permissible at old age to reflect age-induced personality changes (Sheikin, 1996, p. 67).

**Example-9**

*Murun Yrya* (“nasal” song). Old age personal song of Maria Sleptsova-Kustui Maaia, of Yakut-Evenk ancestry, reproduced by her granddaughter, Marina Vasilyeva. Her grandmother used to sing this particular arrangement during knitting. The lyrics list many different garments made by Maria in the past and explain what they are good for (Dyakonova, 2014). The song is characterized by nasalization and *kylysakh*, applied to the simple formula of 3 pitches (“degrees”) (http://chirb.it/4Fc24f).

A PS resembles a renewable passport photograph, identifying principal temperamental shifts throughout life^27^. In this way a PS maintains *personal integrity*, reminding the singer of an anchor-state to return to after an emotional perturbation. This is most obvious in lengthy “autobiographical” PSs, where singers list dramatic events of their life (Ojamaa, 2002). A PS is replaced if the return to the anchor-state somehow becomes impossible.

PS “anchoring” is invaluable in the solitary lifestyle of Northern nomads, who are infamous for psycho-pathological disorders known as “arctic hysteria” (AH) (Tseng, 2003). In the colonial past, this diagnosis reflected the Eurocentric evaluation of the indigenous population by Western physicians (Tseng, 2007), but throughout the twentieth century AH captured the attention of anthropologists (Foulks, 1985). Eventually, AH fell within the scope of cross-cultural ethno-psychiatry as a “cultural syndrome” (Kirmayer, 2018). Such qualification somewhat undermines the life-threatening power of AH (Czaplicka, 1914, p. 307–25), perhaps because the English literature on AH focuses on Inuits, whose symptomatology appears milder and rarer than in the Russian North. Mitskevich (1929, p. 21) reported severe disabilities (and even deaths) in 60% of AH incidences in Upper Kolyma. He considered this to be an underestimate, however, since many concealed AH (13), and locals regarded it a “bliss” not to be medically treated (16). According to a Mitskevich's confidant, 100% of women in his settlement suffered from AH (21). AH ran through many families (24) and sometimes broke into mass “epidemies,” affecting up to a third of the population (25–29)^28^. Many feared contracting AH from others. Even in modern Russia, ethnographers are afraid to research AH-related matters^29^.

Earlier researchers explained AH by the negative influence of extreme cold and Polar night (Novakovsky, 1924). Later research has identified such contributing physiological factors as calcium and vitamin D deficiencies (Wallace and Ackerman, 1960). Similar symptoms were observed among Southern neighbors (Mongols), along with phobias and “copycat” syndrome—attributed to a sufferer's excessive submissiveness (Aberle, 1952). Alcoholism was also considered a contributing factor (Foulks, 1985), although alcohol was exceedingly expensive and thus unaffordable to the indigenous Siberian population before 1917 (Mitskevich, 1929, p. 16). More convincing is Mitskevich's connection of AH with chronic starvation, constant stress, excessive pre-pubertal sexual activities, and sensory deprivation, which affect the Northern population much more than the Southern (45) and are environmental rather than cultural—they are as common among local Russians as among Yakuts (9). Such etiology and epidemiology are confirmed by numerous authors (Tokarsky, 1893; Gamov, 1894; Sieroszewski, 1896; Sakaki, 1903; Bogoras, 1910; Vitashevsky, 1911; Anuchin, 1926; Shreibler, 1927; Shirokogoroff, 1929; Shternberg, 1936; Petrov, 1960; Grigoryeva, 1996).

The reduction in AH must be attributed to the considerable improvement in living conditions throughout the twentieth century^30^. Prehistoric life in a tundra-like climate, then, must have been compromised by even more severe AH.

What remains unknown outside the Russian literature is the connection between AH and music. AH sufferers sing, first quietly, then more excitedly, swinging hands and shivering (Jochelson, 1910, p. 31). Their songs comprise a special genre—*menerik yryata* (crazy songs)^31^—characterized by a confusion of identity and consciousness: the singer haphazardly switches between multiple identities in dialogic singing, with incoherent words and intense timbral modulations (tremolo, rasping, falsetto), raving-like, occasionally shrieking, moaning, and clapping in metric disarray (Alekseyev, 2008). Sufferers report hearing songs of evil spirits, and can ameliorate their suffering by singing the evil spirits' songs, to vent the spirits out (Vitashevsky, 1911, p. 188).

**Examples-10/11**

Ex.10. Reproduction of *menerik yrya*, usually performed during the attacks of *meneriyi* by the anonymous old Yakut from Tattinskii ulus. He described his singing later as a reaction to seeing an evil spirit abaasy in the corner of his yurt and, causing him shriek in deep fear, trying to scare the spirit away. His yelling is interspersed with singing out the lines, supposedly pronounced by *abaasy*. Characteristic is the “dialogic” representation of at least two characters (http://chirb.it/tIandL).

Ex.11. Another *menerik yrya*, imitated by Vissarion Gavrilyev from the Maar settlement in Niurbinskii ulus - according to his experience of frequently witnessing *meneriya* of his neighbor, an old woman. The lyrics are more comprehensible than in the example above and present an argument between the patient and the spirit *ichchi*. The song is characterized by the alternations of a recitative-like excited singing/talking and brief tremolo motifs in a free metric setting, interrupted (rather than accompanied) by spontaneous clapping (http://chirb.it/mr28fk).

AH singing drastically contrasts with the stability of PS and its regular, endless repetitions of the same formula in a characteristic “personal” timbre. This opposition reveals the orderly power of PS and how crucial it is for survival in Arctic conditions. Continuous singing during long, solitary travel in the tundra prevents the rider from falling asleep and losing track of direction, or helps in surviving snowstorms (Krushanov, 1987, p. 234). PS keeps one's mind present under critical pressures.

In contrast, *menerik* loss of personal identity corresponds to suicidal behavior—e.g., running away and freezing to death (Nissen and Haggag, 1988)—supporting the indigenous beliefs that *meneriya* occurs when evil spirits invade one's soul. According to anamnesis, singing starts precisely during the attacks of such “possession” (Mitskevich, 1929). Although pathological singing clearly *results* from AH, there might be some *feedback* between the disruption of “normal” PS use and AH incidents. The Nanai, another PS ethnos, regard any mental disease as a “personality disorder” caused by the invasion of *multiple* spirits (Shimkevich, 1896)^32^. Culture is known to construct methods for handling common mental illnesses, which often include music (Robertson-DeCarbo, 1974). If music can heal, it should also have the power to aggravate—PS disfunction could contribute to AH.

Another musical-psychological anomaly, common in Siberia even now, is *tyyl yryata*^33^—sleep-singing, caused by extreme exhaustion or distress, especially when these are chronic. It resembles sleep-talking and can last for hours until the sleep-singer awakes (Jochelson, 1910, p. 13:37). Locals do not consider this a disease (Mitskevich, 1929, p. 10).

**Examples-12/13**

Ex.12. Genuine *tyyl yrya*, captured by Eduard Alekseyev from an overnight recording of Prokopii Sleptsov from the settlement Druzhina, Abyiskii ulus. Upon listening to this recording, Sleptsov remembered his dream of hunting a moose, but could not recognize the language of his singing (presumably, Yukaghir, a native tongue of his mother, that he later forgot). Singing is based on a brief descending gliding motif consisting of 2 pitches (degrees), possibly with a third complementary degree (http://chirb.it/086zkG).

Ex.13. Reproduction of *tyyl yrya* of an old woman, a relative of the famous Yakut singer, Luka Turnin, who had overheard her singing on numerous occasions. The song is based on the repetitions of a brief formula of 6 tones, engaging 3 pitches (degrees) - most likely a personal motif of the old woman. The lyrics complain about disappointing the barn-master spirit, and promise to please the spirit with a gift (http://chirb.it/cg0cvL).

The formulaic structure of *tyyl yrya* suggests that it constitutes a deeply ingrained PS, “automatically” reproduced by the sleeper. Perhaps *tyyl yrya* is a byproduct of the hyper-activation of the self-identifying circuit that attempts to cope with a stressful exertion, whereas *menerik yrya* reflects the failure of such coping. This would confirm Jochelson's observation that sleep-singing relates to *meneriya*.

In indigenous Yakut culture, there are numerous vocal genres dedicated to meditative psychological self-regulation in stressful situations: *kögus yryata* (pain-reducing songs), *enelgen yrya* (complaining songs), and *sulanyy yryata* (dying songs), which are collectively described as *uiulga yrya* (singing for mental endurance) (Dyakonova and Grigoryeva, 2017).

In Arctic conditions, the significance of PS is truly “Cartesian”: “I *sing*; therefore I *am*” (Sheikin, 2002, p. 330). Loss of PS amounts to “disintegration.”

Musically, a PS reflects one's affiliation with a kin and territory (Sheikin, 1996, p. 12). Specific intonations, rhythms, and timbres characterize specific geographic locations and kin groupings (Novik, 2004, p. 80). Comparative musicological analysis of PSs allows for identification of one's genealogical tree in Samoyed cultures (Niemi et al., 2004). The need for geographic tagging originates in the custom of taking a wife from neighboring ethnoses (Goltsova et al., 2005); territorial PS markers prevent incestuous marriage (Aizenshtadt, 1982). Patriarchic lineage determines the wife's social identification through her symbolic “rebirth,” in which she loses membership in her birth kin upon adoption by her husband's kin (Sagalayev and Oktiabr'skaya, 1990, p. 18). Kin membership legitimizes a human being (Tyukhteneva, 2015). In such a system, the absence of PS amounts to “excommunication” from traditional society and the patronage of supernatural forces.

## The Concept of Musicality in Timbre-Based Music

The existential cardinality of PS increases the value of musicality. In Western societies, a lack of musical abilities bears no biological cost; amusiacs do not suffer damage to their social life (Patel, 2010, p. 371–379). In PS cultures, however, amusia is costly.

Most traditional indigenous societies engage all members in active music production, since musical deficiencies pose serious obstacles to “normal” social interaction (van der Schyff, 2013). Like speech, music-making fuels **autopoiesis—**ongoing biological self-organization that optimizes the antithesis “I”-“non-I” to support one's autonomy (Maturana and Varela, 1980). Essentially, this is no different from the formative role of musical communication in the cognitive development of an infant (Cross, 1999). The absence of such communication endangers the psycho-emotional well-being of the child (Malloch and Trevarthen, 2009).

Inability to sing one's PS is perceived in indigenous societies as a handicap that requires family assistance. That is why, in Chukotka, those incapable of singing have their relatives sing their PS for them (Dyakonova, 2015). According to Siberian mythology, aphonia distinguishes dead souls from the living, and losing one's voice to a spirit equals death (L'vova et al., 1989, p. 90). Through its association with breathing, voice is considered an immanently live object capable of “invading” a person and “subduing” his/her mind (Pashina et al., 2005, p. 50). This is of special concern for shamans, who frequently imitate the voices and personal songs of dead people. Hence, using a “correct” voice is crucial for personal and societal well-being, and in some communities traditions call for the very old and sick to stay silent during public rituals (Dorokhova, 1995).

Unsurprisingly, in PS cultures the standard of vocal musical proficiency is decidedly low. Sieroszewski expressed discontent at “voiceless” Yakuts, who were always singing their “endlessly repeated monotonous tunes,” “pleasant only to [the] performer,” and “annoying like [a] mosquito buzz” (Sieroszewski, 1896, p. 569). His annoyance is evidence that contemporaneous Polish and Russian folksong standards, influenced by the classical *bel canto*, exceeded the Yakuts' in aesthetic demands. Frequency orientation and the absence of PS in Russian folksong enabled the *bel canto* influence, propping up its underlying idea of the necessity for “musical gift” and “proper” education. Such an attitude pushes toward *exclusiveness* of musicking and its professionalization. PS, on the contrary, promotes *inclusiveness*^34^.

Timbral music does not observe “wrong notes”: informants are puzzled by questions about musical mistakes, as they believe that any expression is “right” (Ojamaa, 2003). In addition, intervals in timbral-based cultures are not fixed, as they are in frequency-based cultures, but are stretchable (*ekmelic*) depending on the singer's emotional state (Nikolsky, 2015). A performer, when repeating a song, sets the same lyrics to varying pitches, unaware of pitch discrepancies despite a fine musical ear that is evident whenever he/she sings other styles of music. Indefinite pitch certainly reduces to a minimum the demand on musical skills necessary for a “decent” performance.

A PS can be exceedingly simple, performable by virtually anyone.

**Example-14**

Personal song of a Nenets old woman, Utchi, from the Kazym river region, covered with taiga (courtesy of Triinu Ojamaa). The melodic contour of this song's formula is exceedingly simple—engaging only 3 degrees within the range of only 274 cents: the lowest (228–231 Hz), the middle (249–252 Hz), and the highest (c. 254-267 Hz) degrees (http://chirb.it/0wM28B).

Structurally, a PS consists of “leitmotif” and “leittimbre”^35^—a reused motif and timbral quality. One family's PS can be distinguished from another's by its timbral style (Ojamaa, 2002). Musical elements that stay intact in all uses of a PS represent the PS's owner (musical “foreground”). Elements that change represent the circumstances of his activities (“background”): e.g., riding engages a different rhythm than fishing in an otherwise identical PS. Together, *foreground and background define the position of an individual in space and time*—indispensable in the solitary conditions and open spaces of Siberia.

Discrimination between foreground and background features requires the perception of ***thematicity***—musical material that constitutes a ***theme*** (Mazel, 1979, p. 150–5)^36^. Although thematic analysis evolved within Western classical music (Réti, 1951)^37^, it should nevertheless be extended to all types of music (Val'kova, 1992)—like the Greek *thema* (proposition), which emerged in rhetoric theory and therefore suits any application related to semiotic representation where the perceiver needs to remember a particular expression essential for a musical work (Drabkin, 2001). Matters are complicated by the theme's salience. It might fall anywhere between two poles: “concentrated” (e.g., symphony) or “dispersed” (prelude) (Val'kova, 1992, p. 33)—as of the case in centonization in plainchant (Treitler, 2007). Timbral music also utilizes themes of various salience; JH players “compose” by developing selected thematic material^38^.

PS culture relies on ***monothematicism***: “personal leitmotif-leittimbre” represents the same person. PS users identify themes intuitively, without conscious differentiation between melody and lyrics, but nevertheless *associate musical sameness with personality, and verbal diversity with environmental circumstances* (Ojamaa and Ross, 2004). Worth noting is that altering a PS's melodic contour constitutes a change of ownership between family members, but altering a PS's words does not (Ojamaa, 2002). A person sings one's PS without words when no contextual reference is needed, only a personal reference, such as missing a dear relative. However, changing words *per se* is considered changing the music. Typically, asking one to sing “another song” results in retexting the same **monotheme**. Only upon request to “sing like someone else” would a singer switch from that monotheme to the PS of the person mentioned (Alekseyev, 1988, p. 163–65).

“Timbral musicians” parse songs primarily by lyrics—not surprisingly, since phonemic oppositions constitute the timbral domain (Ojamaa and Ross, 2011)—rather than by pitch, like “frequency musicians” and non-musicians (Bonnel et al., 2001)^39^. Lyric-based parsing is common for Western infants (Lebedeva and Kuhl, 2010). Centering on lyrics generally characterizes the initial stage of acquisition of vocal musical skills (Welch, 1994). The earliest children's non-imitative singing surprisingly resembles an *ekmelic* PS with its stretchable intervals, formulaic structure with retexted lyrics, and private musicking to accompany various activities (Moog, 1976; Dowling, 1984; Bjørkvold, 1992; Campbell, 1998; Barrett, 2011; Koops, 2012). By 7 months, infants can differentiate the timbre of complex tones (Trehub et al., 1990) and remember timbre-specific information (Trainor et al., 2004).

If PS processing requires skills available to normal 1 to 2-year-old children, then amusia in timbral music societies must be virtually non-existent. Indeed, native PS-processing skills are widespread and effective. There are accounts of Siberian non-musicians retaining memory of a once- heard PS for decades (Novik, 2003). Timbral music culture is definitely designed to support this personal thematicism. Niemi et al. (2004) considers the absence of collective singing and instrumental pitch-based accompaniment among Northern autochthons a consequence of the identification functionality of PS^40^.

## Jaw Harp (JH) as a Principal Musical Instrument of Eurasian Timbre-Based Music

Ps provides a model for the “musicalization” of other sounds. Animal calls, wind, rain, etc., all are regarded as the “personal voices” of natural objects, and are characterized by specific timbres and pitch contours (Novik, 1999). Like human PSs, their monotheme could take different shapes to provide information about their “whereabouts”: e.g., Evens distinguish between the sad, angry, and happy “talk” of the fire in a fireplace. Moreover, “fire-talking” is believed to interact with human speech (i.e., respond to human conversation). Every environmental/household object could interact with humans. And this subjects them to the same “personalization” as humans.

Most Siberian **phono-instruments** (Sheikin, 2002, p. 46–67) possess their own “PS”: flask, spoon, or cane, each is easily recognizable by its sound. Environmental sounds too represent “personal owner-spirits.”

**Examples-15/16**

Ex.15. *Vyvko*—the Nenets buzzer used to imitate wind. In the past this had to do with the rituals of calling on rain, but now it is primarily a children toy, promptly made from a thread and a button (http://chirb.it/mcGarg).

Ex.16. *Symysky*—the Khakass male maral call, made from a piece of birch bark (http://chirb.it/8zt1tw).

To a human, such instrumental “PSs” form an “objective” sound-scene collectively representing the surroundings.

And, here, the JH emerges as the simplest archaic instrument capable of producing *multiple* “PSs” of natural objects—it is universally renowned for its onomatopoeia capacity (Maslov, 1911; Beliayev, 1933; Le Roux, 1950, p. 2:507–8; Picken, 1957, p. 186; Koizumi et al., 1977; Alekseyenko, 1988; Zagretdinov, 1997; Bulgakova, 2001; Sermier, 2002, p. 103; Alexeyev and Shishigin, 2004; Mamcheva, 2005; Canave-Dioquino et al., 2008; Yesipova et al., 2008; Suzukei, 2010; Kartomi, 2012, p. 159).

**Example-17**

*Temir-khomus* (Altaic JH) is imitating something that human voice cannot—the sound of the water stream, Tuva (http://chirb.it/aa35p2).

Onomatopoeic versatility explains JH's popularity amongst children, which reflects their desire (typical in traditional societies) to resemble adults^41^.

Onomatopoeic vocalizations play an important role in early verbal acquisition (Menn and Vihman, 2011). Onomatopoeic words are easier to spot, remember, and comprehend (Laing, 2017). Similarly, onomatopoeic sounds facilitate comprehension of JH music, help in mastering the JH, and model new expressive means. The sounds one makes in learning the JH are no different than the babbling that comes from an infant learning to talk; both are playful explorations of the phonemic variants of a selected articulation (Davis and MacNeilage, 1995). The frame/content theory explains not only the acquisition of speech (Davis and Zajdo, 2008) but also of JH playing. If the vocal apparatus constitutes the infant's first “sound-toy” (Papoušek and Papoušek, 1995), for many timbral-based cultures the JH is the second. The “frame” of each JH syllable is filled with selective spectral content according to the same principles of phonetic symbolism that govern verbal acquisition (Shinohara and Kawahara, 2010).

The universal semantic values of ideophones and phonaesthemes (Svantesson, 2017), especially in vowels (Fischer-Jørgensen, 1978), apply to JH articulations. Thus, [i] implies nearness, narrowness, forwardness; [a] broadness, openness; front vowels tenderness, clarity, luminosity; back vowels firmness and concreteness^42^. Many associate sounds with colors/tints.

**Example-18**

The comparative demonstration of the principal khomus articulations: (a) front vs. (b) back, (c) high vs. (d) low vowels—performed by Ivan Alekseyev. Each of these 4 “poles” of JH articulations is characterized by salience of a particular register in generating a respective “JH formant”: 2.4–3.1 kHz for “front,” 0.3–1.6 kHz for “back,” 1.2–2.5 kHz for “‘high,” and 0.6–0.9 kHz for “low” vowels. The most similar are the “back” and “low” vowels, distinguished by greater intensity of the lowest 1st, 2nd, 3rd, and 4th harmonics of the “back” vowel versus the “low” vowel which has much narrower bandwidth of its lowest formant (http://chirb.it/Az51G7).

“Front” syllables are described as tense and unpleasantly “tart.”

**Example-19**

“Front” syllables alone. They require tension in throat, face, lips, jaw, cheeks and tongue—isolating the mouth chamber. Erkin Alekseyev, a distinguished khomus player and the director of the research department at the International Museum of Jaw Harp at Yakutsk, describes his sensations while playing or hearing this articulation as though tasting an extremely sour apple (http://chirb.it/HNwHpq).

“Back” syllables feel pleasantly relaxed.

**Example-20**

“Back” syllables. They require relaxation in vocal apparatus. Erkin Alekseyev describes his sensations as “comfortable” to the extent of feeling “lazy” (http://chirb.it/MCzDkN).

“High” syllables resemble “smiling,” from joyful to sarcastic.

**Example-21**

“High” syllables. They resemble “front” articulation, except that facial muscles remain relaxed. Erkin Alekseyev experiences this configuration as though “smiling” to oneself as in a situation when finding something funny. Emotionally, this state can be charged with joyfulness/playfulness (positive) or sarcasm (negative) – depending on how strained the larynx is (http://chirb.it/n7z749).

“Low” syllables are experienced as “sublime,” yet constrained.

**Example-22**

“Low” syllables. They strongly activate the soft palate, configuring it into a “cupola.” Majority of khomus performers associate it with the sound of a big church-bell—and imagine something sublime and lofty while playing or listening to it. Like “high syllables,” sensing “low syllables” can appear “positive” or “negative”—depending on whether it is accompanied by the exertion of larynx and the discomfort resulting from this (http://chirb.it/6Iy1z8).

JH musicking explores a subject by chaining such elementary “meanings,” interspersed with the referential meanings of onomatopoeic devices. In *syyia tardy*, expressing oneself via the JH is preferable to speaking/singing whenever one feels unclear about something and tries to figure out his/her attitude toward it. JH proficiency enables one to pick any thematic idea and track it, exploring one's emotional state—akin to self-directed speech, yet addressing subjects that are too intangible for verbal expression.

**Example-23**

Demonstration of a typical session of “playing for oneself” on Yakut khomus by Ivan Alekseyev (http://chirb.it/bg32m9).

Numerous North Asiatic ethnicities did not develop frequency-based musical instruments, probably because the JH satisfies the need for an objective reference frame and the PS for a subjective frame, thus leaving no void to fill.

## Complementarity of PS and JH Playing in Timbre-Based Music Cultures

The JH presents a counterpart to the PS. Onomatopoeia, citations of popular melodies, the use of conventional genre and stylistic features, and a “storytelling” compositional approach, common among JH players, all elaborate on environmental *objects*. Moreover, the JH is “anti-personal”: it replaces the player's natural voice.

The JH's lamella makes women, men, the old, and the young sound the same despite all their physical differences. Its capacity to conceal the most telling source of personal information, the human voice, while supporting diverse mimicking, reflects the JH's power to “objectivize” sound. The camouflaging capacity of the JH goes a long way—it even allows aphoniacs to emit sounds (Shimomura, 2016).

Despite its “chameleonic” nature and different constructions, the JH remains easily recognizable (Ledang, 1972). Its “proprietary” timbre is evident in the practice of vocally imitating the JH with help from the fingers, a popular activity among Altai children (Fomin, 2018). Paradoxically, the “proprietary” timbre does not impede the imitation of other timbres; the JH makes an effective **vocoder** (Leipp, 1963). The lamella-generated tone provides a “carrier signal,” and the vocal apparatus, a “modulator filter.” What is unusual is the *hybridization* of different domains: the JH's modulator is human, while the carrier is instrumental.

- The JH is not an ordinary musical instrument that produces a desired tone, it is a musical “centaur”: an organically indivisible human-instrument (Alekseyev, 1991b).

All JH constructions are “centauric”: their instrumental component determines the frequency, while the vocal component—its amplification/attenuation (Dournon-Taurelle and Wright, 1978, p. 21). Since personalization comes largely from the vocal component, the JH becomes “impersonal.” Totally unlike the PS, the JH hides the player's identity, whether in romantic serenading, shamanic rites, animistic hunting rituals, or children's pretend-games.

Vocoding generally relates to hiding. Electric vocoders were invented for ciphering military telecommunications to conceal the speaker's identity (Tompkins, 2010). In popular music, vocoders surfaced in urban genres that replaced a “humanistic” expression with a “robotic” one; they also penetrated other genres to conceal a singer's personal traits (gender, ethnos, class, age) that were perceived as potential vulnerabilities (Dickinson, 2001). Likewise, in animistic societies the JH reduces the risk of the player being identified and hurt while coming in touch with “spirits” (Popov, 1949, p. 265). In modern societies, the JH protects against eavesdropping (Morgan, 2008).

- The JH remedies the undesired ramifications of PS usage.

However, the JH offers a workaround for its camouflaging: it supports personalization through *pitch contours* rather than *timbres*. In the Russian Far East, a player engages individualized patterns, each of which exposes the JH “voice” in its own way—often enabling the recognition of a particular player (Mamcheva, 2012, p. 223). This applies to other Siberian instruments as well. The Udege perceive typical instrumental patterns as the “personal songs” of a specific instrument (Sheikin, 1982). JH patterns constitute “motifs” of “timbral music,” equivalent to PS motifs; the equivalence becomes apparent whenever the JH borrows thematic material from a song (Sheikin, 2002, p. 7).

**Examples-24/25**

Ex.24. “*Hyttya-hyttya, syrdyk k*γ*mm*γ*t”* (Summer is coming), Yakut folk song, sung by Fedora Gogoleva, describes a bright sun looking out from the skies and sending its warmth to mark the beginning of summer. The song is based on a simple 3-degree formula with regular dance-like rhythm in the *degeren* style. However, timbrally, music stays in the *tangalai yryata* style — “palatal singing,” where tongue is abruptly pushed to the soft palate while taking a loud inspiration (http://chirb.it/vpnhv1).

Ex.25. “*Hyttya-hyttya, syrdyk k*γ*mm*γ*t”* improvisation on khomus, Fedora Gogoleva. The same performer takes the melodic formula of the song above (Ex.24) and elaborates its motifs/articulations (*naigryshi*), arranged to form a proprietary JH composition that is intended to express joy and happiness. The JH version loses the traits of “palatal singing,” but keeps the *degeren* style rhythm (http://chirb.it/dp391O).

JH personalization must have emerged *after* its vocoding capacities became established. As archaic cultures lose their animistic ideology, musical instruments turn into producers of a specific musical arrangement, purposed for a specific application, thereby obtaining distinct semantic value and becoming “informative” about their creator (Novik, 1998). Thus, Mansi use instrumental patterns along with PS to display the birthplace/kin of Bear Festival participants (Tchernetsov, 1971, p. 110). Mansi and Khants conduct annual riverboat races where musician crew-members perform patterns representing the locations through which the boat passes (Sheikin, 1990, p. 8–9).

Each musical instrument embodies a particular gesture abstracted from the performing motion, plus the timbre abstracted from that instrument's sound—to form a new modus of musical thinking (Zemtsovsky, 1987). Like any instrument, a musical instrument is a technological tool for the (re)-production of a desired sound/texture^43^. To earn the reputation of a folk instrument for a certain ethnos, the instrument must execute a culturally important function (Stockmann, 1987).

Ainu, Ulch, Evenki, and Indonesian JHs are surprisingly similar in construction, sound, and technique (Duvan, 2003). So are Udmurt, Bashkir, Tatar, and Komi JHs of the Kama region (Aleksandrova, 2017). Nearly identical are the ancient Japanese and modern Nivkh and Karelian JHs (Wright, 2001). Intercultural contacts can hardly explain the commonalities among areas so remote from one another, as attempted by Fischer (1986, p. 156). A more likely explanation is the psycho-acoustic properties of the JH. There are four transcultural traits of JH music:

- Onomatopoeic imitations in “musicking for oneself,” discussed above;
- Concealing the identity of a “serenader”^44^ or his/her message from bystanders^45^ (Hauser-Schäublin, 1995; McLean, 1999, p. 265; Hsu, 2001; Levin and Suzukei, 2006, p. 116–17; Canave-Dioquino et al., 2008; Kartomi et al., 2008; Matusky, 2008; Sam, 2008; Uchida and Catlin, 2008);
- “Transporting” shamans to the other world (Agapitov and Khangalov, 1885; Czaplicka, 1914; Vasilevich, 1969; Galaiskaya, 1973; Rouget, 1985, p. 126; Alekseyenko, 1988; Vainshtein, 1991; Suleimanova, 1994; Maskarinec, 1995, p. 192; Duvan, 2000; Solomonova, 2000; Pegg, 2001; Sarangerel, 2001; Yousof, 2004, p. 70–71; Canave-Dioquino et al., 2008; Honeychurch, 2015, p. 19; Chuluunbaatar, 2016; Tadagawa, 2017b);
- “Supernatural” protection (Popov, 1949, p. 265; Dyakonova, 1981; Dobzhanskaya, 1991; Pegg, 2001; Tadagawa, 2017b)^46^.

Common to all four traits is the representation of *multiple* entities by a *single* JH “voice.”

*The JH acts like a wild card, adopting different identities to benefit the player*. For hunters, the JH attracts prey by imitating their sound (Alekseyenko, 1988), or by pleasing the master-spirit of a particular place to get his favor (Galdanova, 1987). For sweethearts, the JH unites them by appropriating the other's articulations (Haid, 1999). For children, the JH presents unlimited make-believe impersonations (Yekeyeva, 2014). All these applications disregard the player's identity and focus on the impersonation act using the JH as a “fit-all” entity. The JH's vocoding nature empowers it.

In archaic cultures, the JH opposes the PS mainly through the implementation of privacy:

- PS aims at an **individual** by referencing his unique voice to him and his relatives/countrymen;
- The JH aims at the **environment** by reproducing and thereby appropriating its voices, attaining harmony through “centauric magic.”

**Figure 1:**
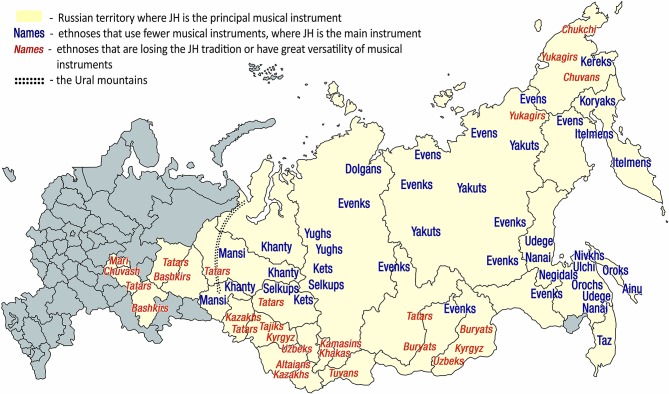
Russian Ural, Siberia, and Far East administrative regions where JH is the principal musical instrument. Such regions are colored beige. This figure displays the area of dominance of the indigenous Eurasian JH traditions according to the current ethnographic data. This area is confined to Russian territory. The names of those ethnicities that favor JH in the scarce musical instrumentarium are colored blue—altogether, 21 ethnicities. Each ethnicity name in the map reflects the areas of that ethnicity's greatest concentration. The only region where the importance of JH tradition remains on par with indigenous ethnicities of Russian Ural, Siberia and Far East (blue names) is Austronesia. Orange color marks those ethnicities that are either losing the JH tradition altogether (e.g., Chukchi) or retain it but of diminished overall significance due to great versatility of folk musical instruments (Tatars). The territories outside of Russia where JH music continues to occupy an important cultural role (approximating those of the orange-marked names of Russian ethnicities) include neighboring Kazakhstan, Uzbekistan, Kyrgyzstan, Turkmenistan, Mongolia, Tajikistan, Northern Afghanistan, Northwestern China and Hokkaido (not marked on this map). The names for JH in indigenous languages are listed in English by Bakx and Crane (2017) and in Russian, by Galaiskaya (1987), and in the encyclopedia of musical instruments (Yesipova et al., 2008).

**Figure 2:**
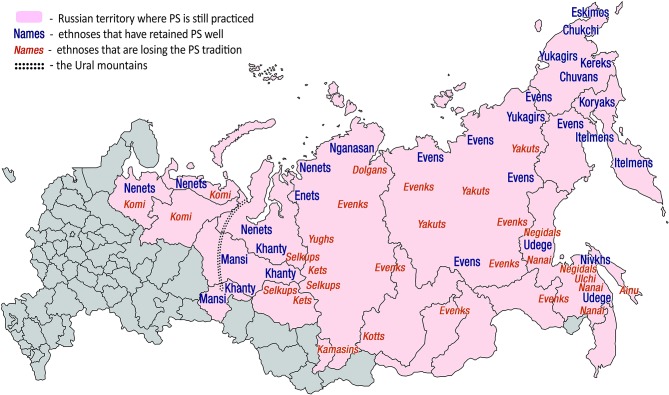
Russian Ural, Siberia and Far East administrative regions where PS is still practiced. Such regions are colored pink. The names of those ethnicities that have preserved PS well are colored blue. Ethnicities that are losing their PS tradition are marked orange. The tradition of PS retains its social significance (blue names) only on the territories of modern Russian Federation. Outside of Russia, this tradition survives amongst Amerindian tribes of the northeastern US and Canada (Ojamaa, 2002) (not marked on this map). This tradition existed amongst Ainu (Sheikin, 1996; Novik, 1999). The indigenous names for PS are: northern Mansi *eryg*, southern Mansi *erei*, Enets *barae*, Nganasan *baly*, Evenk *ikaee* (singer's PS) and *alma* (PS of close relatives or friends), Amur Nivkh *lu*, Sakhalin Nivkh *lund*, Ainu *yayan yuukara*, Chukchi *chinitkin grep*, Koryak *sinkin ulikul* (nominal song), and *ikoleyavan kuli* (kin-song) (Sheikin, 1996), Kerek *kuligul*, Even *tiinmei* (singer's PS), *alma* (someone else's PS) and *ikaan* (“cover” PS that has turned into a popular song), northern Khanty *ar*, central Khanty *arae*, eastern Khanty *lulpany*, Nenets *khybants* (Sheikin, 2002). Comparison of Figure 1 and this figure demonstrates that PS and JH are shared by Mansi, Khanty, Selkups, Kets, Yughs, Kamasins, Evens, Dolgans, Evenks, Yakuts, Udege, Yukagirs, Chukchi, Kereks, Chuvans, Koryaks, Itelmens, Nivkhs, Ulchi, Nanai, Negidals and possibly Ainu. Of them all, Mansi, Khanty, Evens, Udege, Nivkhs, Itelmens and Koryaks have retained both traditions well. Area of their traditional habitat—the Urals, the Arctic Circle, Chukotka, Kamchatka, Okhotsk Coast, Sakhalin and Primorye—must delineate the stronghold of “timbre-oriented music.” Prior to the Russian colonization and Chinese cultural influences, which have both been spreading out the “frequency-oriented music,” this stronghold most probably included also Eastern, Western and Southern Siberia, where today both traditions, of JH and PS, are relatively weak.

Self- and world-orientations complement each other. Not accidentally, the JH and PS share the same territory (Figures 1, 2).

## Animistic Roots of Timbre-Based Music

Initially, the JH worked like a talisman, connecting its owner with a supernatural force via “magic contract” (Novik, 1998). The first JHs were most likely made from grass, e.g., the Nivkh *konga-chnyr* (Mamcheva, 2005); tree twigs, such as the Altaian/Tuvan *taia/yyash-khomus* (Modorov and Dvornikov, 2015); and splinters, such as the Yakut *mas-khomus* (Sheikin, 2002, p. 119). It is exceedingly easy to make such instruments, even for a child; the materials are gathered rather than manufactured. The antiquity of JHs can be deduced from myths ascribing the invention of the JH to a bear—a totemic ancestor for numerous Siberian ethnoses (Startsev, 2017)^47^.

The JH might have originated from the “aeolian harp”—its eerie sounds produced by wind hitting a splintered tree (Duvan, 2003). Siberians believe that lightning purifies trees that it strikes, and items made of their wood have protective power (Suzukei, 1991). Additional protection came from totemic phytomorphism: thus, Nivkhi drew their ancestry from larch, Ulchi from cedar, Oroks from birch, and Ainu from fir (Duvan, 2003). Plants were believed to breathe and, therefore, to possess a soul, possibly to even have PSs, just like animals (L'vova et al., 1989, p. 89). *Live creatures could have PS*.

**Example-26**

“Personal song” of reindeer Urdy, performed “on behalf of him” by his owner, Valentina Kosterkina, in Nganasan. The lyrics describe reindeer's exhaustion from work, complaints on a dog that likes to bite his legs and anticipation of a good rest (Dobzhanskaya 2014, p. 124) (http://chirb.it/M46DrK).

Personalized songs and instrumental patterns are also assigned to spirits to attract their attention, and even musically reflect the mythological family relations between spirits (Gemuyev and Sagalayev, 1986, p. 68). “Spiritual” Ugric PSs were transcribed by Sheikin (1990). They were used to call a patronizing spirit for healing (Voldina, 2017).

Ancestor cults dominated most of Asia. Like mountains, rivers, and animals, plants too were believed superior to humans and could be totemic ancestors. Even today, Khakassian and Altaic kin groups consider larch and birch their ancestors (Sagalayev and Oktiabr'skaya, 1990, p. 50–59). Myths tell of trees nourishing or giving birth to human kin-founders (Tadysheva, 2018). A dying nearby tree is seen as prediction of death for someone from a corresponding kin: for Jyc, fir, Kuzen pine, Komdosh birch, Tubalar aspen, and Todosh honeysuckle (Kypchakova, 2006). Cutting ancestral trees is still taboo in Altai. Trees were actually used to trace genealogical “trees”: thus, *Irkit* kin drew their origin from father-honeysuckle and mother-birch (Potanin, 1883, p. 7).

Zoo/phytomorphic ancestors are depicted on the Uralic metallic disks, 1st millennium BCE, from the Khanty-Mansi Museum (Gemuyev, 2000, p. 63–64); one of them depicts a “plant-woman,” identified by ethnographers as mythological *Por*'s ancestress, birthed by a bear after eating *poryg* (Heraclium sibiricum) (Tchernetsov, 1971, p. 91–93). *Poryg* and similar herbs are commonly used for making musical instruments in Siberia (Sheikin, 2002, p. 4–7); these are sacred for *Por* people. Such instruments probably served as kin talismans before becoming regional musical instruments (Vasilyev, 2016). *Kongon* (Leymus), used for grass JH-making, was also mythologically anthropomorphized into a woman (Kreinovich, 1928, p. 192), and was possibly ancestral to some kin.

Trees serve as kin markers for South Siberian Turks (Sagalayev and Oktiabr'skaya, 1990, p. 43), who distinguish an individual by membership in *seok*^48^ (kin) and believe that the bones of co-members are made of the same “wood” type. Following the tree/forest paradigm, each person corresponds to some tree, and his kin to the same tree-species—in the forest that represents the entire ethnos (53–54). The correspondence of people-grouping to tree-grouping is also observed among Ugric ethnicities (57). In the 2010 census, 82 *seok*s were registered in the Altai Republic (Tyukhteneva, 2015). Standard Altaic identification includes a totemic animal, tree, and mountain (Tadysheva, 2016).

Within this identification system, a plant-made JH would represent an individual, kin, or birthplace by the instrument's “voice.” Initially purely timbral, such identification eventually became “melodic,” engaging personal patterns. This development likely came about through vocoding. Since a wife is considered estranged to her husband's kin even after marriage, and is tabooed to call her husband's totemic entities by name^49^ (Tadysheva, 2018), articulating names on a JH or onomatopoeically hinting at them would circumnavigate the taboo. This could explain the female affiliation of the JH among many Ural-Altaic ethnicities. As a rule, men married foreign women (Pakendorf, 2007), who then became restrained by the taboo. Most archaeological finds of JHs in Ural vicinities come from personal belongings in female burials (Aleksandrova, 2017). Across Eurasia, the JH is one of few artifacts typical for burials, indicating the JH's connection to the supernatural and ancestral (Oleszczak et al., 2018). In the Far East, the JH is still used in lamentations for deceased ancestors (Sheikin, 2002, p. 364).

The ubiquity of the JH in Asia can be explained by its shamanic use (Emsheimer, 1986). *Shamanism constitutes a unified religious system in the entire Uralo-Siberian area* (Alekseyev, 1992). Samoyedic female shamans still use a JH instead of a tambourine (Alekseyenko, 1988), the primary shamanic instrument across North Asia. Around 2000 BCE, the entire area from Bactria to China was strongly influenced by shamanism (Francfort, 1994).

In Altai, novice shamans are restricted to playing the JH until they achieve spiritual maturity (for some never achievable) and receiving a tambourine—a pattern reflecting the antiquity of the JH in shamanic practice (Vainshtein, 1991, p. 273). In Mongolia, shamans use a JH to initiate shamanic rituals and engage the tambourine only upon reaching a trance-state (Chuluunbaatar, 2016). This suggests that the JH's onomatopoeic and vocoding capacities make it more socially inclusive than the shamanic tambourine, as they do not require exceptional “supernatural” powers from the JH's user (Sheikin, 2002, p. 69–134). Surviving beliefs in the personal protecting capacities of the JH reveal its origin as an egalitarian “magic” tool, preceding the professionalization of shamanism and the establishment of the tambourine as one of its attributes.

JH musicking surpasses PS in its accessibility. Across northeastern Asia, toothless people use a hammer or ax to play JH (Tadagawa, 2017b).^50^ In Yakutia, the ax and JH are even welded together^51^. The JH is used as an aid in *alalia* (Everstova et al., 2019). Complete aphoniacs play the JH (Shimomura, 2016)—and probably have been doing so for a long time in the Far East. The usage of JH-like devices (*koouchyntzyy*) to aid in aphonia was recorded during the Song dynasty (Picken, 1957). Such “prosthetics” highlights JH's great importance in native cultures.

Together, a JH and a PS provide “axes of coordinates” in the animistic worldview. The PS defines the “subjective” aspect as follows (Sheikin, 2002, p. 255):

1. “Thus I sing” (individual);
2. “Thus my countrymen sing” (territorial);
3. “Thus my family sings” (genealogical).

These hierarchic levels also reflect an evolutionary progression, inferred from cross-examination of all existing Siberian traditions. Sheikin adopts the Even culture for a model, because it has retained all three levels (263–8)^52^. In the past, Chukchi observed a similar hierarchy^53^.

An analogous three-stage model applies to the JH, defining its “objective” aspect:

1. “This JH protects me in my environmental encounters” (individual);
2. “These onomatopoeic JH imitations control their original owners from my environment” (territorial);
3. “This JH style optimizes my kin's relationship with the environment” (genealogical).

This model might apply to other Siberian mouth- and phono-instruments.

The intra-cultural interaction of the PS and the JH promotes the emergence of JH patterns. Thus, Nivkh players often engage the same pattern for different compositions (Mamcheva, 2012, p. 222).

**Examples-27/28**

Ex.27. Nivkh JH PN by Vera Khein, as presented in her original improvisation which she named “The flatfish dance.” This piece, like many others that were recorded by Natalia Mamcheva from Vera Khein's, is based on her personal motif consisting of two descending intervals: of a 3rd and a 2nd (e.g., E-C-Bb), where the latter is often marked by the shorter rhythm of the middle tone (C) and its multiple repetitions, marking the lowest tone (Mamcheva, 2012, p. 297–300) (http://chirb.it/aIrCvG).

Ex.28. Nivkh JH PN by V.M. Persina, as presented in her improvisation (No. 130 from Appendix 1, Mamcheva, 2012, p. 301). It uses 2 motifs: of an ascending 2nd (passing C-D-E or auxiliary C-D-C) and of an ascending auxiliary 3rd (E-G-E), where C and E are marked as anchors, and the middle tones (D and E) are often given shorter rhythmic values (http://chirb.it/2L65H3).

Nivkhs also differentiate JH patterns by specific timbral words matched to a genre^54^ (Sheikin, 2002, p. 132).

**Example-29**

The Nivkh rhythmic formula “Kan Vai,” typical for various instruments and vocal music, in JH music is characterized by the onomatopoeic dog-like articulation of “khav-khav” (Mamcheva, 2012, p. 119). This formula distinguishes the genre of a dog-racing music, used in festive competitions that are held by Nivkhi, Ulchi, and Negidals. This genre is also used during the sacrifice of a dog in an annual bear festival (103) (http://chirb.it/q2xxbJ).

The amalgamation of non-onomatopoeic JH devices must have accompanied the historic development of PS: timbral themes prototyped **timbral words**. Many indigenous Siberian cultures employ specific vocables^55^ as ethnic/territorial markers (Sheikin, 2002, p. 251). Their timbral and rhythmic makeup could direct JH improvisation toward elaborating standards. Thus, Yakut *khomusists* frequently engage the timbral vocable “*hyttya*.” Timbral words comprise “timbral phrases,” employed as in magic incantations (Tchernetsov, 1987, p. 35)^56^. Animistic hierarchy somehow transforms into syntactic hierarchy.

As the traditional lifestyle modernized, animism and totemism lost relevance, along with the need for anonymity. Subsequently, playing the JH acquired melodic/rhythmic/timbral patterns idiosyncratic for a player. They differ from a PS by engaging a *repertory of playing devices* rather than a single *monothematic* device.

- The JH was preferable to the PS whenever timbral music was “about” an **external** (to the performer) “object” rather than an **internalized** “subject^57^.
- The JH's **plurality** matched an “objective” perspective, while the PS's **singularity** matched a “subjective” perspective with its single “self” (for a mentally healthy individual).

## Jaw Harp Articulations vs. Singing and Speech

Musical and verbal vocalizations adopt opposite parsing strategies (Alekseyev, 1993):

- Comprehensibility of **elements**. Speech prioritizes the clarity of contrasts to mark discrete elements in the speech-flow. **Division** prevails through timbral **contrasts** (oppositions).
- Comprehensibility of **gestalt-total** (melody). Singing prioritizes the integration of sounds into a melodic line to secure an empathic response to it. **Unification** prevails through timbral **similarities** (equalization).

The JH's vocalization introduces tonal homogeneity and monotony. Both are considered detrimental to speaking and singing, reducing the intelligibility of their prosody. JH prosody is unaffected by this. *It relies on contrasts only of the mid/high-frequency spectrum, confining monotony to FF*.

In frequency-oriented cultures, singing requires timbral uniformity while stressing the FF changes (Titze, 1988). Voicing every consecutive utterance by *definite pitch per se* homogenizes the spectrum of sung tones^58^. Like singing, verbal intonation engages FF changes even in non-tonal languages (Ladd, 1996), while stressing spectral contrasts, as JH does.

- The JH **combines singing and verbal approaches**. Its monotony conceals the player's identity yet preserves all articulations: “timbral words” become monotonously “flattened.” Such “flattening” presents a form of timbral equalization similar to singing.

**Figure 3:**
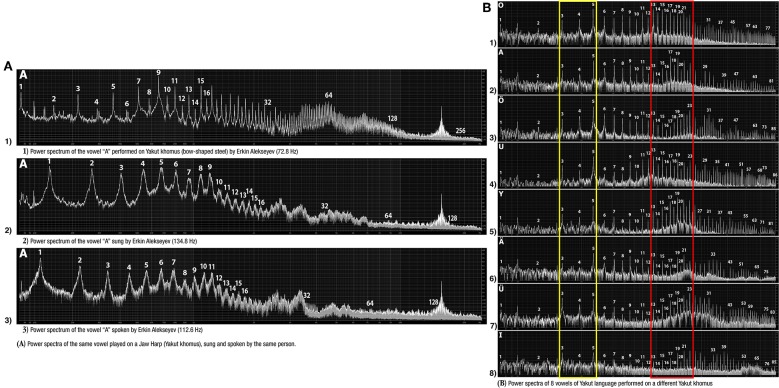
Characteristic acoustic features of the JH vowel articulations. This figure demonstrates the importance of harmonic structure for the JH vocal system. The numbers on the curves mark the harmonics. Vertical axis indicates amplitude, and horizontal frequency. **(A)** Power spectra of the vowel [a] performed on khomus, sung and spoken. (1) Khomus. There are 145 harmonics discernible above the noise floor, peaking at f9. (2) Singing (bass). There are 23 active harmonics, peaking at f1. (3) Speaking. There are 19 active harmonics, peaking at f1. Weakness of formants seems to be part of the indigenous Yakut prosody—judging by the recordings of native Yakut speakers we had at our disposal. In their number of active harmonics, position of the loudest harmonic and distribution of peaks (2) and (3) are harmonically closer to each other than to (1). JH reserves higher intensity for odd harmonics, as opposed to speaking and singing. The bandwidth of harmonics is significantly narrower for khomus than for singing and speaking. If to measure the bandwidth at the harmonic's baseline level, the khomus f2 occupies a range between 141 and 151 Hz, making 117 cents (a semitone). The f2 of the singing “A” ranges between 243 and 297 Hz (349 cents—a neutral third). And the f2 of the speaking “A” ranges between 197 and 238 Hz (325 cents—a narrower third). The last notable difference is a much smaller noisiness of the khomus “A”—easily detectable in the absence of the “furry” looking jiggles in the contour line. These jiggles are very prominent in the singing of “A” and even more so in the speaking of “A,” especially >f7. The recordings were made on the recorder Ritmix RR-989, with the sampling rate of 44.1 kHz and the bit depth of 16 bit, and analyzed by the software application RX Pro by iZotope with the following settings: 262144 samples FFT size, Hann window, 4x time overlap, average channel mode, 80 dB/s decay, extended log frequency scale. The peak at 15.6 kHz reflects a permanent background noise in the room. The analysis was the average of 4 versions of (1), (2) and (3). **(B)** Power spectra of “active harmonics” of all Yakut vowels articulated on Yakut khomus. The vowels are numbered according to their perceived “pitch values” in an ascending scale (Ogotoyev, 1988), according to the convention common amongst Yakut JH players—which generally coincides with the comparative vowel's height. JH's “instrumental formant,” F1, marked by the yellow rectangle, falls on f5 in all vowels except “Ü” and “I,” where F1 broadens to f3–f5. In contrast, “vocal formant,” F2, marked by the red rectangle, falls on different harmonics in 6 vowels: f13 for “O,” f17 for “A,” f23 for “Ö,” f19 for “Y,” f21 for “Ä,” and f21-f23 for “I.” Only 2 vowels, “U” and “Ü,” engage the same harmonics as, respectively, “O” and “Ö.” However, “U” differs from “O” by a smoother shape and consistent dynamic prevalence of odd over even harmonics (>f13). “Ü” differs from “Ö” by an even greater contrast of odd/even harmonics and louder high harmonics >f47. Altogether, the harmonic composition of F2 varies by the number of active harmonics, the ratio of the intensity of odd to even harmonics and the magnitude of dynamic “dropouts” amongst the adjacent harmonics in the harmonic series. Therefore, each vowel possesses its unique harmonic configuration of F2. The recording was made as in **(A)**. The audio clip representing each vowel was selected out of a pool of 15 performances by 3 performers on 3 JHs based on averaging the dynamic values for the lowest 11 harmonics and finding the closest match to the average harmonic profile for each of the vowels.

Both singing and speech vowels stress FF (Figure 3). But speech's clarity depends more on frequency **changes** of F1 and F2 (Zsiga, 2013) that are sufficient for distinguishing vowels (Carlson et al., 1970). Singing **freezes** F1 at about the same frequency level for all vowels (Sundberg, 1974).

- Singers intuitively match vowels. Speakers oppose them. So do JH players—but only in relation to the “**residual** tone,” not the “**fundamental** tone.”

The concept of “residual pitch” was introduced by Schouten (1940) and adopted by modern theories of harmonic perception (Goldstein, 1973; Terhardt et al., 1982; Moore et al., 1984, 1985, 1986; Houtsma and Smurzynski, 1990; Moore, 2012; Norman-Haignere et al., 2013). The sum of the unresolved partials that comprise “pitch residue” is perceived as a pitch identical to FF, but harsher in timbre (Schouten et al., 1962).

Unresolvability does not prevent pitch perception, because pitch can be analyzed by an alternative mechanism: through the autocorrelation of nervous impulses, activated by partials, in a phase (Licklider, 1951). Despite its complexity, such hearing is exceedingly common (Moore, 2012, p. 217). However, unlike FF recognition, it requires learning (Terhardt, 1974). Hence, periodicity recognition comprises a cultural phenomenon.

- JH prosody engages monotony to facilitate residual pitch analysis^59^.
- A drone-like FF turns into “background,” while changes in residue comprise “foreground” in an auditory scene analysis of JH music. Its users focus on the foreground and ignore the background.
- As in a PS, the unchangeable JH “background” represents the hidden “self.” The changeable “foreground” represents the “world.”

JH users distinguish one articulation from another most likely by learning and memorizing a number of “harmonic templates,” pairing them with the perceived sounds in a search for the best match (Goldstein, 1973). Such templates are formed by a long-term accumulation of statistical data, and tend to rely on the harmonic series (Shamma and Klein, 2000).

Musical templates might be influenced by phonological templates that are generated by the pitch analysis of the tonal attributes of speech (Schwartz et al., 2003). JHs made of organic materials usually deviate from the harmonic series in the tuning of partials. A JH can also omit a harmonic (usually f2), or generate a non-serial partial or a subharmonic (see Appendix-1 in Supplementary Material).

- JH's “centauric” nature makes JH's prosody **hybrid** (Alekseyev, 1991b).

The **equalized** FF portion of the JH spectrum resembles the singing formant (Sundberg, 1974), while JH's “residue” portion, in contrast, marks every **opposition** of phonemes. Hence, the *JH can “talk” AND “sing”* **sequentially** as well as **simultaneously**. JH prosody engages two different domains, vocal and instrumental, where the vocal controls the dynamics and the instrumental the frequency of the harmonic components (Trias, 2010). Each domain introduces its own formant(s) (Figure 3):

1. F1 is **instrumental**; it homogenizes JH articulations and depends mostly on JH's construction.
2. F2 is **vocal**; it differentiates between JH articulations and depends mostly on the vocal tract configuration. Additional vocal formants are present at least in “high” vowels [i:].

JH vowels retain F1 at the same level (per instrument), but move vocal formants. Each vowel has its unique harmonic signature comprised of:

- the number of harmonics over the noise floor (“active” harmonics),
- their dynamic envelope within the spectrum,
- an odd/even harmonic ratio,
- the harmonic composition of formants.

The border between the “instrumental” and “vocal” ranges lies ≈f6–f8. Therefore, we must introduce a new concept of “**harmonic base**” to encompass f1–f4(f7) in contrast to “**harmonic residue**.”

JH formants differ from speech/singing formants for the same vowels (Leipp, 1967). A JH vowel engages six times more “active” (i.e., salient) harmonics than singing, and almost eight times more than speaking. F1 in the JH is much softer than in speaking and singing. The JH's F2 is much stronger. The JH's harmonics display a much greater odd/even contrast than singing/speaking vowels do. JH harmonics are much narrower in bandwidth and are compromised by less noise.

**Harmonicity** (Plack, 2010), which has no direct parallel to the JH for singing and is absent altogether from speech, is definitely crucial for JH prosody (Nikolsky et al., 2017). Very likely, harmonicity/inharmonicity forms the principal axis in the discrimination of JH articulations (Nikolsky, 2017). Indigenous users must have high sensitivity to the harmonic structure of sounds. Thus, Nanai believe that everything sung will certainly be heard by spirits who do not recognize human speech (Bulgakova, 2000). The harmonicity of singing could be responsible for this.

The hybrid nature of JH prosody poses the question: is it derivative of speech, like a grammelot (Jaffe-Berg, 2001); of singing, like a kazoo; or is it ancestral to both language and music?^60^

## Establishing the Time and Place of Origin of Indigenous Eurasian JH Music Traditions

**Figure 4:**
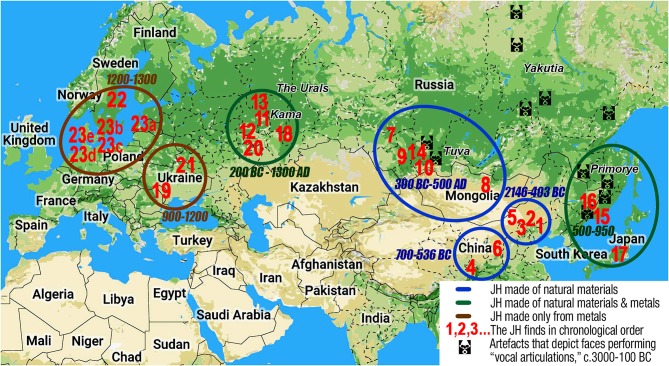
Worldwide geographic distribution of archaeological finds of JH, dated prior to the fourteenth century, and of artifacts that depict faces performing “vocal articulations,” dated c.3000–100 BCE. This map is designed to reflect JH's distribution pattern throughout Eurasia prior to the fourteenth century—the beginning of JH mass production, trade inclusion and, subsequently, export to European colonies. Numbers in red reflect the chronological order of JH finds. Their dating and publication sources are referenced in Table 1. Circles mark the geographic clustering of finds and are accompanied by their dating range. Blue color marks JHs made of natural organic materials (bone, bamboo, wood), brown—of metallic JHs, and green—of mixed (organic and metallic). The most ancient finds cluster within the Inner Mongolia and are all organic. Next in timeline are Southern China and Altai, both of which also house organic JHs. Still next is the cluster covering Ural Mountains and Volga planes. It includes mixed materials. Two subsequent areas to the West, Ukraine and Northern Europe, feature exclusively metallic instruments. Manchuria and Japan form another cluster, concurrent with the Ukrainian, and feature mixed materials. The black face icon marks the archaeological sites containing petroglyphs, sculptures, masks or ceramics that depict human faces with open mouth—“singing masks” (Okladnikov, 1968, 1971; Okladnikov and Zaporozhskaya, 1972; Devlet, 1976, 1980; Okladnikov and Mazin, 1976; Brodianskii, 1978; Leontyev, 1978; Arkhipov, 1989; Kochmar, 1994; Devlet and Devlet, 2011)—exemplified in Figure 5. Most singing masks petroglyphs precede JH finds and concur with the Shuiquan JH in Inner Mongolia. The oldest petroglyphs are located at Sikachi-Alian, Amur, 3rd millennium BCE (Okladnikov, 1971, p. 83–89), and the most recent—the utmost northern location in Yakutia, at Olenek River (Arkhipov, 1989; Kochmar, 1994). Geographically, the locations of “singing masks” coincide with the JH finds in two regions: Tuva and Primorye, but chronologically precede the JHs excavated in those locations, suggesting that JH articulations grew out of timbral singing, probably of PS. Absence of “vocal articulations” in Inner Mongolia could be explained by the lack of attention of Chinese archeologists, not distinguishing such pictures from other images [in the same vein, Chinese archaeologists had difficulties recognizing JHs in the unearthed materials of a number of sites (Shulga, 2015; Kolltveit, 2016)]. On the other hand, absence of JH finds in Central and Southern Yakutia and Kamchatka, where images of “vocal articulations” were found, might be due to the wet climate that makes preservation of organic materials unlikely.

Archaeological evidence points to Inner Mongolia as JH's homeland (Figure 4).

**Figure 5:**
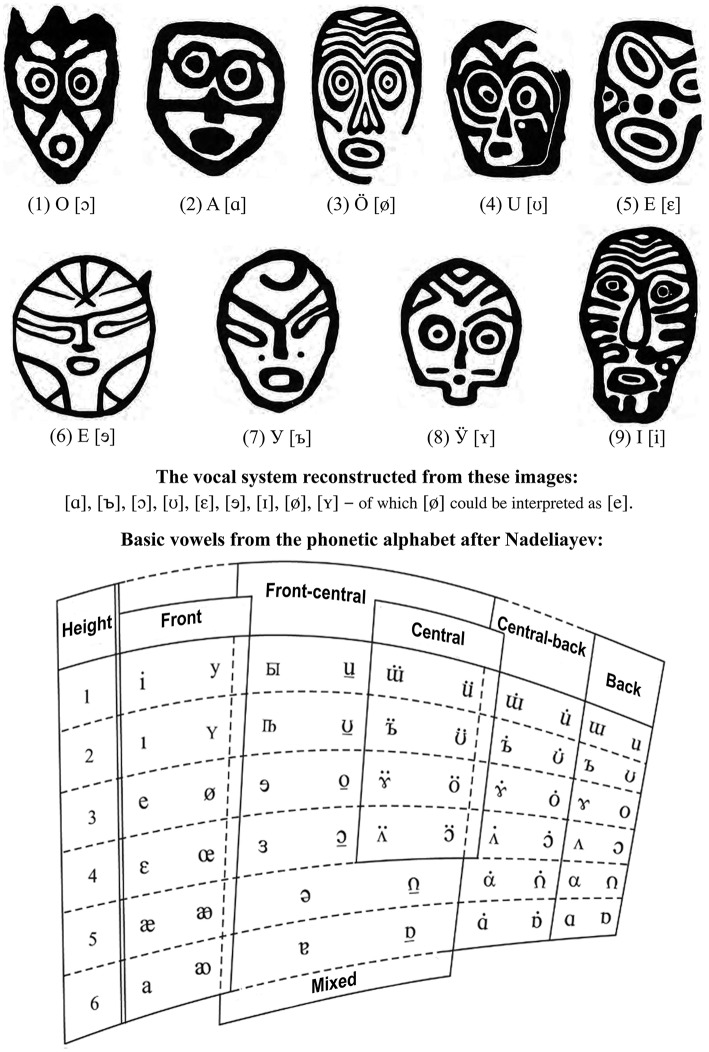
“Singing masks” from Sikachi-Alyan, Amur river, dated c. 3000 BCE (Okladnikov, 1971, p. 83–89) and their phonetic interpretation. These 9 images were selected by Sheikin (2002, p. 259), who believed that they depicted pronunciation of vowels in order to perpetuate a model for articulating sacred names, important for the ancestor cult. Such images are found near Amur river along the border between modern Russia and China, and differ from most East-Northern Siberian petroglyphs by focusing on not animals, but humans. Specifically, these faces emphasize the configuration of open mouth and the emotional expression in an individualized manner where no one image reproduces any other image (Okladnikov, 1971, p. 85). Similar in style, “singing masks” are found in few isolated sites to the West of Primorye, in Tuva, and to its north, in Yakutia and Kamchatka, suggesting the enormous expansion of a Neolithic Far Eastern culture during the Bronze–Iron Ages (Andreyeva, 1987). Each mask could hint at a particular sacred word tabooed for public use—similar to the current practice in the Altai region. The vowel harmony, omnipresent in Uralic and Siberian languages (Kiyekbayev, 1972), especially strong in Turkic languages (Gadzhiyeva, 1997), in effect, encodes the configuration of the mouth for the entire word, since all its syllables are bound to reproduce the same vowel (e.g., Yakut miitary call “*urui*”). Sheikin's set of images is identified as pictorial representation of a vocal system of a hypothetical “Amuric” Paleo-Asiatic language by I. Seliutina, N. Urtegeshev, T. Ryzhykova and A. Dobrinina from the Laboratory of Experimental Phonetic Research at the Institute of Philology of the Siberian Department of the Russian Academy of Science. Based on the methodology of the founder of this institute, Nadeliayev (1960), this lab has collected an extensive database of articulations of basic phonemes of Siberian languages (Seliutina et al., 2012). Although the lack of uniformity in Neolithic images limits the researcher to estimate the gradations in labialization and only a gist of its palatability, however, Nadeliayev's classification of labialization (Nadeliayev, 1980) allows the phonologist to identify vowels based on the ratio of mouth width to height. The “Amuric” vocal system is presented in the phonetic alphabet developed after Nadeliayev by 9 of his pupils: I. Seliutina, A. Urtegeshev, A. Letiagin, A. Shevela, A. Dobrinina, G. Esenbayeva, A. Savelov, M. Rezakova, and Yu. Ganenko (Seliutina et al., 2012). The basic vowels from this alphabet are shown in a table at the bottom of the figure. The “singing masks” are numbered in the order of the ascending JH “articulatory scale” as formulated by Ogotoyev (1988), with the correction by Shishigin (2015) that places “o” rather than “a” at the bottom of the scale. The mask images (Sheikin, 2002) and the phonetic table (Seliutina et al., 2012) are reproduced by permission of the authors.

The oldest JH belongs to the Lower Xiajiadian culture, 2146–1029 BCE (Kolltveit, 2016). Its proximity to the Manchurian sites where the Neolithic “singing masks” were found (Okladnikov, 1971) indicates that JH articulations might have originated from timbral singing (Figure 5). “Singing masks” always stress the configuration of mouth, nose, and eyes in a single face, omitting the body; in contrast, the animalistic art of the neighboring Siberian cultures presents the entire animal, and usually in groups (Okladnikov and Mazin, 1979, p. 86). Each “mask” depicts a specific vocal articulation charged with an emotional expression that may represent an ancestral attribute (Sheikin, 2002, p. 259).

Modern Nanai tell myths about a Skull Horse-rider and paint burial idols' faces to house dead souls, closely resembling skull-like Sikachi-Alyan petroglyphs (Okladnikov, 1979). The Nanai habitat includes Manchuria, and the JH remains their principal instrument. “Singing masks” could capture the symbolic meaning of a particular articulation and assign it to an ancestral figure as its “call”—like an animal call ascribed to a totemic ancestor. The model for this could be military calls, *urany*, used by Turkic peoples to distinguish a kin (e.g., Yakut “*urui*!”) up until the nineteenth century (Sagalayev and Oktiabr'skaya, 1990, p. 21). Such calls were believed to possess supernatural power, making them suitable for the vocoding “protection.”

Vowel harmony characterizes most languages of the Altaic Sprachbund (Ko et al., 2014), especially of the Turkic language family (Gadzhiyeva, 1997). There, the Yakut vocal system (Antonov, 1997) is considered the most ancient, representative of proto-Turkic (Tenishev, 1984) and proto-Altaic languages (Tenishev, 1997). The acknowledged paleolinguistic studies date the break of proto-Altaic by 8000–10000 (Andreyev and Sunik, 1982), 7000 (Starostin, 1991), or 5000 BCE (Robbeets, 2015, p. 506)—earlier than the earliest of the Sikachi-Alyan images (Okladnikov, 1971, p. 83–89). “Singing masks,” then, could capture the articulation representing a totemically important meaningful “harmonic” word, such as “*urui*”^61^.

“Singing masks” spread over Amur/Ussuri, Baikal, Lena, and Aldan, dated 3000–100 BCE (Okladnikov and Zaporozhskaya, 1972; Okladnikov, 1974; Devlet, 1976, 1980; Leontyev, 1978; Arkhipov, 1989). This distribution mostly coincides with the oldest archaeological JH finds and modern areas of greatest JH popularity. The northern Far East became culturally isolated around the Bronze Age and retained Neolithic traits until the seventeenth century Russian colonization (Andreyeva, 1987), effectively conserving the archaic traditions.

“Singing masks” are also depicted on Malyshevo ceramics, 2000 BCE (Okladnikov, 1968, p. 50). Similar Neolithic stone and clay masks were excavated in Primorye (Brodianskii, 1978). In Yenisei burials, near Sayan, “real” gypsum masks were found, manufactured ≈2000 BP, probably following some earlier tradition of mask-making from easily decomposing materials (Devlet and Devlet, 2011, p. 310–12). Nenets, Khanty, Mansi, Evenk, Udege, Kumandin, Shor, and Buryat shamanic masks likely represent the same tradition.

Closely related are *tsam* masks (335–37). *Tsam* is a theatrical ritual dance, performed in Lamaistic monasteries across Buryatia, Tuva, Tibet, Nepal, and Bhutan. To distinguish the tsam protagonists from each other, their masks express different emotions, assisted by melodies/rhythms that are specific for each protagonist. Shamans too accompany mask-wearing with vocal and theatric impersonations of evil or good spirits (Zabolotskaya, 2011). Instead of masks, Tuvan shamans use maskoids—small, mask-like plates, mounted on a hat or knitted, called “human face” (Devlet and Devlet, 2011, p. 313). Maskoids usually reproduce conventional face-masks and represent the ancestral spirits guarding the maskoid's owner (Avdeyev, 1960).

The mask's protective function (Ivanov, 1975) resembles JH's “masking”: both are employed by shamans to repel evil spirits.

“Singing masks” likely belong to the cultural Neolithic/Mesolithic “confederacy,” stretching from the Volga-Ural region to Tibet and China (Leontyev et al., 2006). Its influence may have extended farther to Southeast Asia and Oceania (Okladnikov, 1968, p. 52–64). *In this entire area the bamboo/wooden JH remains the principal musical instrument*.

Archaeological data confirms the vegetative origin of the JH. All BCE finds, except for Perm's, occurred in Asia (Table 1). The JH crossed the Urals into the Volga planes ≈200 BCE (Aleksandrova, 2017) thereafter heading toward Hungary and Ukraine. Finno-Ugric peoples might have carried the JH to Northern Europe.

**Table 1:** The earliest Jaw Harps found by archaeologists.

**#**	**JH type**	**Location**	**Country**	**Dating**	**References**
1	2 bone idioglot	Shuiquan, Jianping, Liaoning	China	2146–1029 BCE	Kolltveit, 2016
2	1 bone idioglot	Xiajiadian, Chifeng	China	1200–600 BCE	Honeychurch, 2015
3	4 bamboo idioglot	Yanqing, Beijing	China	770–403 BCE	Tadagawa, 2016
4	5 bamboo idioglot	Yuhuangmiao, Sichuan	China	700–600 BCE	Shulga, 2015
5	4 bone idioglot	Jundushan, Hebei	China	700–500 BCE	Honeychurch, 2015
6	1 bamboo container	Baojiang, Shaanxi	China	576–536 BCE	Tadagawa, 2017a
7	1 bone idioglot	Dubrovinskii Borok, Kolyvanskii District, Ob'	Russia	300–100 BCE	Borodovskii, 2007
8	1 bone idioglot	Morin Tolgoi, Altanbulag soum, Tuv aimag	Mongolia	300 BCE-100 AD	Honeychurch, 2015
9	4 bone idioglot	Tcheremshanka and Tchultukov, Altai	Russia	300 BCE-600 AD	Borodovskii, 2017
10	1 bone idioglot	Aimyrlyg XXXI, Tuva	Russia	200 BCE	Tadagawa, 2016
11	1 bone idioglot	Makhoninskoye, Chastinskii district, Perm'	Russia	200 BCE-300 AD	Aleksandrova, 2017
12	1 copper idioglot	Ust'-Brykinskii, Laishevsky district, Tatarstan	Russia	300–500	Aleksandrova, 2017
13	7 bone, 22 idioglot bronze	Prikamye burials and settlements, Udmurtia, Bashkortostan, Permskii, and Kirovskii regions	Russia	300–1300	Golubkova and Ivanov, 1997
14	2 bone idioglot	Sakhsar, Khakassia	Russia	400–500	Tadagawa, 2016
15	1 iron idioglot	Adrianov Kliuch, Partizanskii district, Primorye	Russia	500–600	Leshchenko and Prokopets, 2015
16	3 iron idioglot	Nikolayevskoye, Shaiginskoye and Smol'ninskoye, Mikhailovskii district, Primorye	Russia	698–926	Leshchenko and Prokopets, 2015
17	2 iron heteroglot	Hikawa shrine, Omiya, Saitama	Japan	900–950	Tadagawa, 2007
18	1 silver idioglot	Idelbayevckiye burial mounds, Salavatskii district, Bashkortostan	Russia	900–1000	Mazhitov, 1981
19	1 iron heteroglot	Echimauti, Rezina district	Moldova	900–1000	Feodorov, 1954
20	2 bone, 2 bronze idioglot	Tankeyevskii, Bulgur nekropol, Kuibyshevsky district, Tatarstan	Russia	900–1100	Aleksandrova, 2017
21	1 iron heteroglot	Hlukhiv, Sumi district	Ukraine	XIII century	Pashkovskii, 2012
22	1 iron heteroglot	Uppsala	Sweden	XIII century	Kolltveit, 2006
23	5 copper heteroglot	Riga, Skanor, Ribe, Greiswald, Hamburg	Latvia, Sweden, Germany	XIII–XIV centuries	Kolltveit, 2009

*The numbering reflects the chronological order from the oldest dates to the newest. “JH type” shows the number of JHs found in a given location, the material from which they were made and the type of construction they use. “Source” cites the publications that contain descriptions of each find*.

Archaeological finds form 6 clusters: Mongolia->China, Mongolia->Baikal, Baikal->Ural/Volga, Volga->Ukraine, Manchuria->Japan, and Western Europe. Bamboo/bone JHs comprise two of the oldest clusters. Ural/Volga and Manchuria/Japan combine both organic and metallic JHs. European JHs are exclusively metallic. This clustering is exceedingly important, because metallic and organic JHs significantly differ in **spectral texture**.

## Spectral Texture and its Dependence on Technological Evolution

The concept of musical texture (Skrebkova-Filatova, 1985) has not been applied to JH music. However, JH players definitely arrange spectral components in a way similar to parts in polyphonic music. Polyphonic thinking is common in a great many indigenous music cultures (Jordania, 2006), timbral music included. Tuvan vocal “solo-polyphony” is well-known. JH traditions also engage 2- and even 3-part polyphony (Brodsky, 1972). Dobzhanskaya (1991) pioneered the polyphonic notation^62^ of JH field recordings; an earlier attempt had been made by Pugh-Kitingan (1977) for a Huli JH excerpt. Polyphonic analysis must be used to investigate JH textural typology. It is hardly possible to comparatively study JH traditions without accounting for the distribution of thematic material within the spectral texture.

**Figure 6:**
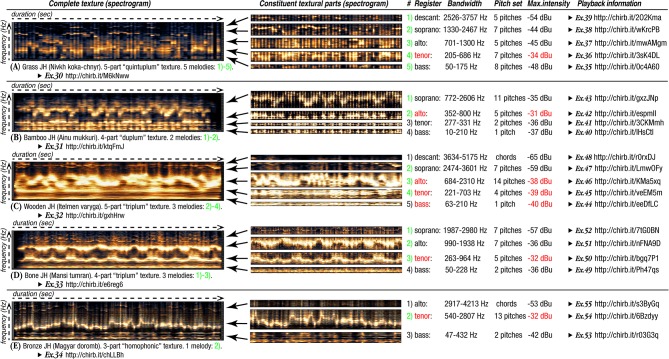
Spectral textures of music performed on JHs made of 5 different materials. On the left, spectrograms show the spectra of JHs made of each of the 5 different materials, most commonly used in Northeastern Eurasia by indigenous players. The horizontal axis indicates time, the vertical axis frequency, and the color-coding amplitude (from black for silence to bright yellow for maximal intensity). In most cases, the texture spreads from about 70 Hz up to 3–5 kHz. On the right, the entire texture is broken in “parts” (bands), each capturing the frequency range within which the “active” harmonics form a continuous (as much as possible) melodic stream. The procedure for defining these parts is described in the Appendix in Supplementary Material. Arrows point to the original position of parts. To the right of the parts are their names, following the standard nomenclature of Western choral music. Each part's bandwidth, amplitude and number of pitch classes (i.e., pitch set) are listed on the right. A pitch class is defined by a dynamic spike repeatedly marking the same frequency level (plus/minus a quartertone) throughout the clip. The dBu value reflects the greatest amplitude for a given part of the texture within the entire clip. The red color marks the most intense part that presumably serves as the principal melodic line for the JH player. Whenever there are a few parts with many pitch classes that reach amplitudes that are close together, they are considered “polyphonic” melodies. The green color marks the “melodic” parts. Each of the complete textures and their constituent parts can be auditioned by downloading the corresponding audio file from the provided web link or looked up in the zip archive in the Supplement. For the description of Audio Examples Nos. 31-55, see the file “List of Audio Examples.docx” in the Supplements. **(A)** Grass JH (Nivkh). 5 Components, each melodic (including the bass). Each part uses similar number of pitches. The leading melody is in tenor. The other 4 melodies are of similar intensity, except the highest one. This texture can be called *scattered “quintuplum” polyphony*, since all of its parts are constantly moving in a similar way, making it hard to track their changes by ear. The only controlled part here is likely to be the tenor. **(B)** Bamboo JH (Ainu). 4 Components, of which the upper 2 are melodic. Bass sustains a pedal tone. Tenor is restricted to an ongoing figuration of 2 adjacent pitches (“principal” belonging to the harmonic series, and “auxiliary” that does not belong to the harmonic series but is a step away from the “principal” pitch). All parts are nearly equal dynamically. Soprano contains the most embellished hemitonic melody which uses twice more pitches than does the anhemitonic alto. This is a *well-differentiated “duplum” polyphony* of soprano and alto, with the drone accompaniment of tenor and bass. **(C)** Wooden JH (Itelmen). 5 Components, of which 3 middle ones are melodic. Bass sustains a pedal tone. Descant alternates between a few ostinato cluster-chords (one principal, others auxiliary). Three lowest parts are nearly equally intense. The polyphonic contrast is tonal (4 anhemitonic pitches for tenor vs. 14 chromatic pitches for alto). This is a *fairly differentiated “triplum” polyphony* of tenor, alto and soprano, accompanied by the pedal bass and pedal-like descant. **(D)** Bone JH (Mansi). 4 Components, of which the upper 3 are melodic. Dynamically, their relation resembles **(C)**, but the stronger tenor is likely to lead. However, tonally, there is little differentiation between 3 melodic parts. This is a poorly differentiated “triplum” polyphony, accompanied by the bass pedal. **(E)** Bronze JH (Magyar). 3 Components with a single melody in tenor. Tenor is the most dynamically and melodically salient part. Its melody is accompanied by the pedal bass and the ostinato “cluster-chords” in alto. This is a *well-differentiated homophony*. Black horizontal lines break 5 textures in 3 groups based on their structural similarity. **(A)** Stands out by its maximal polyphony and the absence of differentiation of parts. **(E)** Stands out by its absent polyphony and maximal differentiation of parts (pedal-melody-ostinato). **(B–D)** Have limited selective polyphony. Of the three, **(B)** features the strongest, whereas **(D)**—the weakest differentiation. **(C)** Features the greatest complexity. The complete spectrograms were calibrated to blur the display of the most intense parts of the spectrum in order to better indicate the melodic continuity across pauses, along the timeline. Blurring was achieved by maximizing the FFT size up to 65,536 samples, with the multiresolution STFT, no frequency overlap and time overlap 4x-16x, depending on the audio material. The window is Gauss 200 dB. Frequency is displayed in mels. The spectrogram of a complete texture zooms in from about 0–100 Hz to 4,000–9,000 Hz, depending on the audio material. Each clip was noise-reduced and dynamically normalized. The clips of textural “parts” have slightly increased frequency zoom and reduced blurriness (greater frequency and time overlap) to display finer detail. The audio of “parts,” uploaded for audition, is normalized in order to make the parts comparable.

The JH's texture (i.e., the structure of the harmonic residue and harmonic base) depends on the instrument's material (Figure 6, see the archive “23 figures for Appendix,” in Supplementary Material, for higher resolution images).

Grass and metallic bow-shaped JHs form two poles of textural arrangement. A grass JH generates **maximal polyphony**: five undifferentiated melodic parts moving simultaneously and only partially controlled^63^. A metallic JH generates **homophony**: a single melody with accompaniment. Bamboo/wood/bone engage **well-differentiated polyphony**: bi-melodic for bamboo and tri-melodic for bone and wood (see Appendix in Supplementary Material).

The order of the increasing discretization of parts in these textures corresponds to the order of the growing complexity of manufacturing a JH from a given material (Table 2).

**Table 2:** The correspondences between the complexity of manufacturing JHs, JH's spectral texture, and JH's sound qualities.

**#**	**Material**	**Availability**	**Manufacturing**	**Texture**	**Complexity**	**Sound**
1	Grass (Leymus)	Readily available in grasslands, albeit seasonal, and disposable	Needs no tools, takes about a minute to make for anyone (Mamcheva, 2012)—minimal expertise required	“Scattered” (haphazard) polyphony of 5 melodic parts	All parts are indistinct and melodically similar, most likely only the dynamically marked tenor is controlled, while the other parts are “aleatoric,” all parts are hemitonic or chromatic, including bass	“Hollow” clicking, very short, dry, yet soft and muffled, clattering at high intensities
2	Bamboo (Bambusa, Phyllo-stachys, Dendro-calamus)	Readily available in a warm and moist climate, evergreen, non-disposable	Needs basic cutting tools, takes days to dry and minutes to make with stone tools (Bar-Yosef et al., 2012)—manufacturing is somewhat complicated by anisotropy—some expertise required	Duplum polyphony of 4 parts: upper pair carries 2 melodies, lower pair accompanies	All parts are clearly defined: alto has the principal diatonic melody, soprano—secondary chromatic melody, tenor—a dyadic or triadic ostinato figure (or a chord), bass—a pedal monotone; alternatively, tenor can carry the 2nd melody while soprano—the pedal chord with slight variations	“Hollow” rattling or rasping, relatively dark, rich, and quite homogenous tone, prolonged decay, introducing tremolo at high intensities
3	Wood (Larix, Betula, Salix, Alnus, Picea, Cedrus, Populus)	Available in accessible lands with temperate mesothermal or continental microthermal climate, all year round, non-disposable	Needs cutting and shaving tools, takes days to dry and about a few hours to make with stone tools (Crabtree and Davis, 1968)—manufacturing is greatly complicated by anisotropy, orthotropy, hygroscopy, variance in quality, knots and spiral grains—extensive expertise needed	Triplum polyphony of 5 parts: middle trio carries 3 melodies, marginal parts accompany (the upper part can be absent)	Melodic “trio” is partially differentiated between secondary anhemitonic melody in tenor and primary chromatic in alto, but not in regards to the tertiary chromatic melody in soprano, while 2 pedal parts—monotone in bass and chordal in descant—frame the melodic “trio” (alternatively, there could be just 4 lower parts—without a descant)	Clattering, dense and rather dry, with a distinct “clang” on the onset, which is more pronounced at high intensities, overall bright tone with medium long decay—great variety in tone for different woods
4	Bone (cortical) and antler	Available from animals practically everywhere, all year round, non-disposable	Needs cutting and scraping tools, takes weeks to soak (Robinson, 1942), hours to cut and about a day to carve with stone tools (Sidéra, 2011)—manufacturing is much complicated by anisotropy, hygroscopy—extensive expertise needed	Triplum polyphony of 4 parts: upper trio carries 3 melodies, while bass—pedal chord or dyad	Melodic “trio” is poorly differentiated except for the bass that sustains a harmonic pedal (triad or dyad), tenor usually carries the principal hemitonic melody, while alto—the secondary chromatic melody (but this can be reversed), and soprano carries the tertiary melody—chromatic like secondary melody	“Hollow” clucking, less definite in pitch than in bamboo and wood, more gentle, well-rolled decay, quite homogenous, medium bright tone, quite long-lasting
5	Metal (copper and its alloys, iron, steel)	Available in hard to access ore deposits, usually in mountains, nearly all year round, the most long-lasting	Needs finding of ore, mining and transporting it to the smelting site, pit-digging, carbon and hermitization to sustain high heat, stationary equipment to extract, smelt and forge metal, takes hours to heat (Coghlan, 1939), days to forge (Bronson, 1996) and tune (Jaago, 2009)—demands professional expertise	Homophony of 3 parts (unavailable on non-metallic JHs)—with the melody in the middle, but possibly in the upper part while chords in tenor (4- and 5-part textures, like those on bamboo or wood are also possible, especially on idioglot JH)	All parts are perfectly differentiated and easy to track: the pedal bass, the rich melody in tenor, and the ostinato “chords” in alto (the upper pair can be reversed); in alternative scenarios an extra part can be added in soprano, which contains a pedal chord, while tenor or alto carries a secondary melody—overall, metallic JHs produce the greatest variety of textures; the principal melody usually has the most degrees of all materials (especially for steel), hemitonic or chromatic, vs. monotone or dyadic bass and triadic-based chord in tenor or chromatic (cluster-like) chord in soprano	Ringing or buzzing, resonant, very full and rich, the brightest, the most well-defined in pitch and the most homogenous in tone of all JH materials, with the longest decay—great variety in tone for different metals and alloys (metal seems to support much greater control of fine tonal detail than non-metallic materials)

*The numbering in the table reflects: the progressive increase in complexity of the manufacturing technology, the decrease in availability of the material to the JH manufacturer, the increase in discretization and variability of the components of the spectral texture, and the increase in timbral richness, fullness and homogeneity of the sound—that characterize each of the materials [for a more complete information see Table 1 in the Appendix and “Textural typology for Appendix.xml” (Supplementary Material)]. The identified pattern of evolution could potentially apply not only to JH, but to other musical instruments made of the same materials. This possibility calls for additional research*.

Materials fall into three classes according to texture, timbre, and playing technique (Table 3).

**Table 3:** Classification of JHs based on similarities and differences in sound quality, spectral texture types and playing techniques between the JHs made of different materials.

**#**	**Raw material for making**	**Traditional JH models of this type**	**Their standard organological description**	**Basic playing motion of a hand**	**Characteristic sound quality**	**Textural typology**
1	Grass leaves, tree bark or tree chips (or splinters), or twig of a shrub or a bush	Nivkh and Ulch *konga chnyr* (Mamcheva, 2005), Yakut *mas khomus* (Dyakonova, 2017), Tuvan *charty-komus* (Suzukei, 1989), *yiash-komus* and Telengit *taia-komus* (Suzukei, 2006)	Idioglot plucking idiophone, frameless lamella (frame-shaped, but open on one side or wishbone shaped)—classified as “proto-JH” by Sheikin (2002)	Touching the leaf, gently tapping on the chip with a finger, or pinching it with two fingers, tapping on the twig	Non-percussive very soft hollow clicking sound, homogenous and simple (the spectral components are poorly isolated from each other, no way of playing melodic modes based on harmonic series, no sustained drone component)—brighter and denser	“Maximal” polyphony of 4–5 indiscrete poorly defined parts, each of which is moving at the same time and sounds similar to the other ones
2	Bamboo culm, wood beam or fragment of fresh cortical bone	Nivkh *tyf-kanga* and *khar-kongon* (Mamcheva, 2005), Ainu *mukkuri* (Nobuhiko, 2008), Evenk *kongiipkavun* (Sheikin, 1986a), *Tumran* of the Ugric peoples (Alekseyenko, 1988)	Idioglot plucking idiophone, framed JH whose plate contains a rope for energetic plucking or pulling as well as a handle (the lamella is framed from all sides, is elongated and bottle-shaped)	Abrupt pulling of a rope by the right hand—JH supports a greater diversity of technical devices (due to greater intensity that exposes more detail) than for the proto-JHs	Loud, low, dry, and harsh sound that lacks in homogeneity (“dirty”) due to either rattling, rasping or bifurcated “clang” and good or superb separation of parts (can stress few parts at the same time, plays the pentatonic harmonic scale and uses the clear bass drone)	“Optimized” polyphony of 4–5 discrete parts, most of which are functionally and/or thematically different (melody, counter-melody, accompanying melodic figure, or pedal chord, dyad or tone)
3	Metal ores	Nivkh *pasla vyt' kanga, zakanga* and *vychranga* (Mamcheva, 2005) and Buryat *aman khuur* made of local “red copper” (Simukhin, 2006)	Idioglot plucking idiophone, bow-shaped JH whose lamella is bent into a hook and attached to the frame (shaped like a horse-shoe); an alternative construction is heteroglot, which most commonly utilizes brass and reproduces the bamboo/wood constructions and the corresponding polyphonic textures	Hitting or strumming the lamella forward, backward, pressing or just holding the lamella with a finger, firm or loose holding of the ring by teeth or lips, right or left shift of the frame (Sheikin, 1986b)—greater diversity than in organic JHs	Prolonged ringing or buzzing sound, harmonically richer and louder than JHs made from organic materials, especially in high range, even greater resolution of detail (displays the content of each part, can play chromatic and microchromatic melodies, place the melody in any part except in the bass, support superb isolation of spectral components)	“Simple” or “complex” (with a few different accompanying parts) homophony with a single melody that is more diverse (use more degrees) than polyphonic melodies of non-metallic JHs—for idioglot JHs; yet heteroglot metallic JHs in addition to homophonic texture use the same polyphonic textures as bamboo/wood JHs

*In general, different hand action in production of sound on a JH corresponds to a different sound quality (Mamcheva, 2012, p. 53–55) and textural typology (see the Appendix and listen to the audio examples in the archive “Audio for Appendix” in Supplementary Material). Their correspondence is somewhat complicated by the cumulative nature of indigenous JH traditions, exemplified in Nivkh JHs (Mamcheva, 2005): newer technologies of manufacturing JH tend to adopt the standards of playing from older previously existing technologies, before the novel constructions allow to forge new standards (see Appendix in Supplementary Material for the discussion of this issue). Therefore, metallic JHs are used to generate textures characteristic for bamboo, wood or bone JHs—as well as proprietary “metallic” textures, unproducible on JHs made of organic materials*.

This order suggests the development toward simpler/clearer typologies where each textural type constitutes a stage that builds on a previous stage.

Obviously, the “metallic stage” comes after the “organic.” Based on a comparative ethnomusicological perspective, Sheikin (2002, p. 132) offers further distinctions. He defines the “bamboo stage”^64^ as representative of Oceanic cultures (exemplified in mukkuri) and preceding the “bone/wood stage” of Siberian peoples. Sheikin's periodization can be enhanced. Wooden JHs likely replaced bamboo as the JH spread north. Bone must have replaced wood in the further expansion to the tundra, where wood is rare. Hence the “bone stage” is likely to have succeeded the “wood stage.”

Geometry/construction was another factor that determined the rise of homophony: only the bow-shaped metallic JHs are homophonic. Nevertheless, both factors nearly always^65^ coincide (Fox, 1994; Sheikin, 2002; Kolltveit, 2016). In contrast, frame-shaped idioglot JHs do not seem to support homophony in indigenous traditions. Therefore, *the adoption of bow-shaped metallic JHs ended up replacing JH polyphony with homophony*.

**Figure 7:**
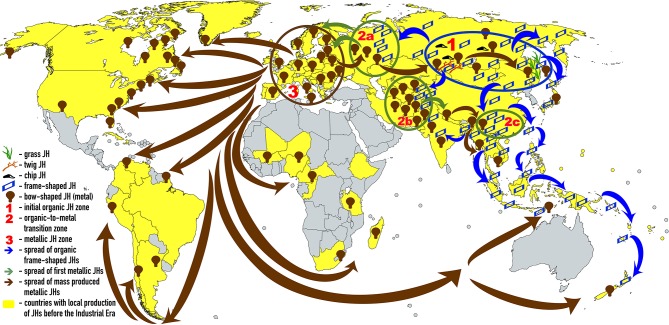
World distribution of JH made of organic and metallic materials prior to the Industrial Period. This map combines archaeological and ethnographic data on local production and consumption of JH across the world, excluding mass international trade from Europe, in order to establish the routes of the spread of metallic and non-metallic JH indigenous traditions. Yellow color marks those countries where JHs were produced and widely consumed by local population. Such countries were selected based on the integration of findings of linguistic research on the presence of native words for JH in local languages (Fox, 1994; Sheikin, 2002, p. 411–503; Bakx and Crane, 2017), of archaeological finds (de Ramón and Rivera, 1982; Beck et al., 1983; Barr, 1994; Kungurov, 1994; Yakovlev, 2001; Pignocchi, 2002, 2004; Crane, 2007; Wright and Impey, 2007; Wright, 2011; Honeychurch, 2015, p. 20; Whitridge, 2015) [plus references from Table 1], and of geo-ethnographic research (Vertkov et al., 1963; Galaiskaya, 1973; Fox, 1988, 1994; Sheikin, 2002; Wright, 2004; Yesipova et al., 2008; Suzukei, 2010; Mamcheva, 2012). Geo-ethnographic data was also used to distinguish between the areas of distribution of bow-shaped metallic and frame-shaped organic JHs. Five icons with corresponding pictures mark the geographic locations of musical cultures that use JHs made of different materials. Red numbers indicate the zones of distribution for different types of JHs—encircled in different colors. (Number 1) Marks the blue oval that shows the area where organic-made JHs have been prevalent (see Figure 4). This area most likely constitutes the cradle of JH music. (Number 2) Marks the green ovals that encircle the areas where metallic JHs have coexisted with organic ones. It is in these areas, that presumably, the transition from organic to metallic instruments took place. Two areas [(2a) and (2b)] correspond to the oldest centers of metallurgy (see Figure 8). The transition probably occurred first in 2a), where the access for import of organic JHs from zone (1) was geographically broader and easier than in (2b), and organic JHs were likely to have been established prior to the wide spread of metallic artifacts. Transition in (2c) area most likely occurred much later because of the later arrival of metallurgy (see Figure 8). (Number 3) Marks the brown oval that encircles the area where exclusively metallic JHs were cultivated. The color-coded arrows show the probable direction for spreading of the organic and metallic JH technologies. Green arrows indicate the distribution of the initial handicraft metallic JHs that might have emulated popular wooden/bone/bamboo types. Brown arrows indicate the distribution of the mass-produced bow-shaped metallic construction, prevalent in Europe. Across the ocean, these arrows connect the European dominions with their offshore colonies. Over the land, brown arrows indicate the chain of cultural contacts that led to the establishment of local production centers of metallic JHs. Evidently, one of the routes of this spread went contrary to the original spread of the organic JHs—from European West to Far East, over the Steppe Belt's borderline with the northern forests via the chain of neighboring Turkic cultures that all favor metallic JHs. This route earned the nickname of “Fur Route” (Rubinson, 1992; Bunker, 1993). Another route went from Central Asia to China via the Silk Road—historic as well as prehistoric, as recent research suggested (Christian, 2000; Kuzmina, 2008; Barinova, 2013).

It is imperative to determine the timeline of the distribution of bow-shaped instruments (Figure 7).

Organic JHs emerged in Atlai/Baikal/Mongolia/Primorye during the pre-Metal Age and spread north, south, and west. Grass and chip probably succeeded twig, followed by chip-in-frame and frame-idioglottic instruments—corresponding to the advance from purely “sonoric” effects to the pitch-oriented manipulation of overtones (Sheikin, 2002, p. 124). An example of an intermediate “chip-in-frame” construction is the Tuvan *charty-komus* (Suzukei, 1989, p. 65–6).

In the Volga/Kama/Ural region the westward expansion of frame met a wave of eastward expansion of metallurgy to introduce metallic JHs (Aleksandrova, 2017). Initially, these seem to have imitated the popular organic constructions (Golubkova and Ivanov, 1997). By the Sarmatians' time, a single standard for frame-shaped instruments (10.3–12.5 × 1.4–1.7 × 0.1–0.2 cm) was established across Central Asia and southern Siberia (Borodovskii, 2017). *This must have corresponded to a single standard of polyphonic texture*.

Farther westward propagation of JHs involved only metallic instruments; wood/bone JHs were used in Europe exceedingly rarely and only before the heteroglot metallic constructions became established (Kolltveit, 2006, p. 83)^66^. The bow-shaped construction was invented somewhere in Ukraine/Balkans/Alps/Karelia, providing a new, simplified texture that co-existed with the older textural standard. However, the exclusive cultivation of bow-shaped JHs must have established a new textural standard that spread all over Europe and its overseas colonies^67^. On land, bow-shaped JHs spread eastward over the same territories where organic JHs had already settled, eventually reaching the Far East^68^. This brought about the co-existence of two autonomous traditions among Nivkhi, Ainu, Evenks, Evens, Kets, Yughs, Selkups, Chukchi, Itelmens, Koryaks, Kereks, Yukagirs, Khanty, Mansi, Tuvans, Yakuts, Dolgans (Sheikin, 2002, p. 125–6), Mongols (Pegg, 2001), and Chinese (Li, 1956). The two traditions differ in playing techniques, sound quality, and texture, to the extent of bearing different names among the same people (Li, 1956; Yakovlev, 2001; Sheikin, 2002; Mamcheva, 2012). The closer to the Far East, the greater the discrepancies. This geographic distinction is supported by the gender/age distinction. Frame-shaped JHs constitute the female/children's sphere of use—bow-shaped the male/adults' (Tadagawa, 2001; Sheikin, 2002; Dyakonova, 2017)^69^.

Significantly, the traditions differ in their mythological status. For Yenisei peoples frame-shaped JHs represent the “voice” of a local deity in charge of successful hunting (126), whereas metallic bow-shaped JHs are an attribute of power and prestige, entitling the owner to protection (131). The ideological divide also involves an aesthetic aspect. Across the entire southeastern end of Russia, all cultures that contain both frame-shaped and bow-shaped JHs consider the former suitable for learning the JH, but not for “serious” music-making (Sheikin, 2002, p. 131)^70^.

Hence, each model follows its own course. Around Kama, bow-shaped JHs supplanted frame-shaped JHs (Yakovlev, 2001). Generally, the archaic polytheistic belief in sacred places yielded to a monotheistic cosmology under the influence of Christianity after Russian colonization (Alekseyev, 1992, p. 215). This weakened the kin-tree/kin-bone correspondences that provided ideological support to the frame-shaped tradition. The strengthening of top-deity cults, the Christian-like dichotomy of good/evil, and divine protection supported the “ownership-based” (not kin-based) protection of bow-shaped JHs.

**Figure 8:**
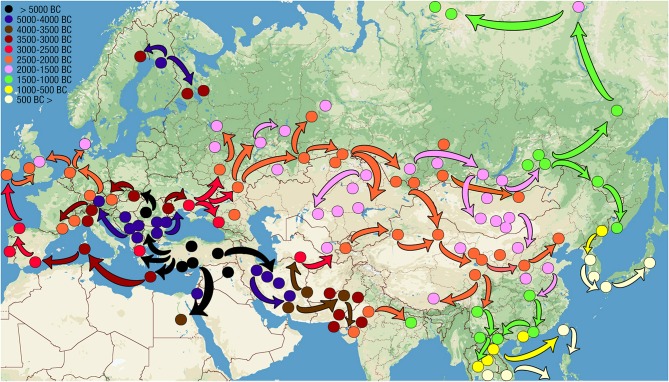
Historic spread of copper metallurgy in Eurasia. This map displays the locations of earliest regional centers of smelting copper ores—according to the available archaeological research on the earliest metallurgy (Chernykh, 1966, 1992, 2012; Sunchugashev, 1975; Zhuravlev, 1977; Sergeyeva, 1981; Prakash and Tripathi, 1986; Rybakov, 1987; Kon'kova, 1989; Thiel, 1989; Mishra, 1994; Reedy, 1997; Kiriushin, 2002; Tylecote, 2002; Nguyen, 1986; Baipakov and Taimagambetov, 2006; Simukhin, 2006; Yanin, 2006; Ciarla, 2007; Hauptmann, 2007; Kaniuth, 2007; Park and Gordon, 2007; Subbotina, 2008; Herva et al., 2009; Roberts et al., 2009; White and Hamilton, 2009; Radivojević et al., 2010; Erb-Satullo, 2011; Higham et al., 2011; Wan, 2011; Potts, 2012; O'Brien, 2014; Garner, 2015; Gelegdorj, 2015; Mei et al., 2015; Hung and Chao, 2016; Huo, 2016; Tripathi, 2018). The routes and timeline of its spread suggests the spread of metallic JHs along with the new homophonic tradition of JH music. The timeline is indicated by color-coding of the circle-icons that mark the location of smelting sites: the darker—the older. The epicenter of metallurgy is in modern Turkey and Iraq, although a concurrent independent center might have existed in Vinca culture, in Balkans, Belovode (Radivojević et al., 2010). Anther independent center emerged in Fennoscandia (Herva et al., 2009) and the neighboring Karelia (Zhuravlev, 1977). Copper metallurgy spread from Balkans to Ukraine, thereon bifurcating, with one branch heading to the northeast forest zones of Russia (up to Ural Mountains), while the other moving southeast through the Steppe Belt all the way to Zabaikalye and Mongolia. There, again the route bifurcated: north, going to Yakutia, and south to Primorye. The other wave from the epicenter reached Iran, branching in 3 directions: toward Indus' valleys, Himalayas, and Middle Asia, through Turkmenistan, Uzbekistan and Kazakhstan to the Uyghur territories via the route that later became known as the Silk Road (Kuzmina, 2008). Around Lop Nor this branch met the outshoot of the Steppe Belt route and proceeded through modern China to its North and South kingdoms. The southern branch eventually hit Indochina, then heading to Indonesia and Pacific islands. This entire area, Manchuria and Japan were the last to develop metallurgy—no earlier than in Middle Ages. Therefore, if for Volga-Ural area frame-shaped JHs co-evolved with the metallic JHs, for cultures at the eastern end of Eurasia bow-shaped JHs came from West and formed its own special niche, different from the already established frame-shaped JH cultures. The bow-shaped tradition must have taken a long time to develop—as metallic artifacts were becoming more affordable to local population. Another zone where frame- and bow-shaped JHs form different cultural traditions is the vast area from southeastern Indochina to Papua New Guinea. The two zones where metallic bow-shaped JHs must have penetrated concurrently with organic frame-shaped JHs where both might have shared the same or similar forms of use, are Himalayas-Pamir-Tian-Shan mountains and Yunnan-Myanmar area.

The autonomy of frame- and bow-shaped traditions is corroborated by the late arrival of metallurgy to the north Pacific coast (Figure 8). The Chinese ideograph for the metallic JH (*tieyehuang*) confirms that the bamboo JH (*huang*) preceded it (Tadagawa, 1991).

The scenario in which the bow shape was the descendant of the frame shape also received organological support (Sachs, 1917; Dournon-Taurelle, 1975; Sheikin, 2002; Suzukei, 2010). Organological analysis of European archaeological finds indicates that European bow-shaped JHs form their own lineage of morphological development from earlier Asiatic samples brought to Europe through trade (Kolltveit, 2006). Currently, the archaeological consensus holds the organic JHs as the prototype for the metallic via westward expansion from northeast Asia (Fox, 1988; Wright, 2004; Kolltveit, 2006; Honeychurch, 2015; Aleksandrova, 2017; Turbat, 2017; Oleszczak et al., 2018). Another expansion is likely to have followed from South China to Austronesia (Blench, 2004).

The time frame for the genesis of organic JHs can be established by dating the migration of the Siberian population to America. Many North Amerindians use the PS (Ojamaa, 2002).

**Example-56**

The topahti—a personal Nootka song of inherited origin, performed by Joe Titian. This topahti was given at the inter-tribal marriage between Nootka and Kwakiutl as a dowry, and permitted for performance only by its owner and her children (Halpern, 1974) (http://chirb.it/NvahDq).

But no JH usage is known before the Western colonization (Wright, 2011)^71^. This is hard to reconcile with the evidence for the common genetic ancestry of Native Americans and south Altaians 23000–18000 BCE (Schurr, 2015). How could an instrument so important for Siberian peoples be totally missing from their American descendants? The Altai-Sayan region is home to the JH. According to genetic and linguistic evidence, Yakuts, whose national symbol is the *khomus*, descend from there (Pakendorf, 2007). Siberia and Alaska remained connected until 8000 BCE, but glaciers blocked Alaska from the rest of the continent until 11000 BCE (Dixon, 2015). Sequencing mitochondrial genomes from pre-Columbian South American skeletons 8,600–500 BP indicates that a small population entered the Americas through a costal route 16,000 BP (Reich et al., 2016). This date comes close to the earliest archaeological evidence of a human presence on the North American continent: 13,000 BP (Anderson and Bissett, 2015).

The most plausible scenario is that the JH tradition did not exist along the northeast Asian coast before 10,000 BP, when access to America was open. Throughout the Quaternary Northeastern Asia enjoyed a remarkably stable climate, and lowlands remained ice-free (Zamoruyev, 2004). The prevailing landscape of the non-glaciated area of Altai was desert-steppe, covered with grass, with patches of woody vegetation at stream valleys—changing into coniferous forest or forest-steppe only in the Holocene (Hais et al., 2015). Similar vegetation covered Mongolia and Tuva. Modern forest-steppe regions, e.g., Ob'-Irtysh Baraba, remained steppe throughout the early Holocene until 5500 BP (Zhilich et al., 2017). In highlands such as the Chuya Alps, the first forestation is dated 7000 BP (Agatova et al., 2012). Forestless landscape stretched toward North America, including Beringia (Hoffecker and Elias, 2007). The tundra belt extended to 57°N, then turned into steppe that covered most of Eurasia (Tarasov et al., 2000). Forestation was prominent only much farther south, at Taiwan's latitude, during the late Pleniglacial (Hope et al., 2004)—too distant from Beringia.

The scarcity of trees would have prevented the formation of the “person/kin/ethnos=tree/species/forest paradigm” that underlies the Siberian timbral music culture. The emergence of “sacred” tree cults depends on trees' importance in sustaining a human population, which requires that they be available in abundance. Otherwise, plants' proprietary “voices” cannot be discovered by population groups large enough to institute a musical tradition.

## Conclusion

Not all known music systems abide by the principle of discrete changes in pitch. In fact, many music cultures rely on timbral changes in their TO. One trait such cultures share is the prevalence of the “personal” over the “collective” use of music, upholding the opposition between “timbre-centered” (definite timbre/indefinite pitch) and “frequency-centered” (definite pitch/indefinite timbre) music systems. Current psychoacoustic research seems to support this opposition.

The large concentration of “timbral” cultures in the former USSR prompted research (often emic^72^) on their TO, promoted by the centralized infrastructure of research institutions, “top-down” funding (official ideology favored “people's music”), and the intense development of ear-training research. The accumulation of vast ethnographic and archaeological data over much of the twentieth century made it possible to create a “historic” perspective on the evolution of folk music. The integration of sciences within Soviet academe supported a multi-disciplinary approach that combined fieldwork with experimental testing. The gathered evidence suggests that “frequency-” and “timbre-centered” musics contrast each other in their TO, patterns of usage, and their social implementation.

Timbral TO relies on PS in vocal, and JH musicking in instrumental domains. Every member of such traditional society possesses at least one PS that serves for personal identification. Musical elements usually indicate family relations and birthplace through similarities with other individuals' PSs. Locals have a keen ear and memory for cross-relating thousands of PSs throughout their life (better than for remembering faces)^73^.

PS contributes to mental health in the traditional Siberian lifestyle. Ongoing singing throughout one's day secures consistency in self-consciousness under environmental stress, common in severe Siberian conditions, thus protecting one from psychological disorders. Pathological singing accompanies multiple personality disorder (*meneryia*) and sleep-singing disorder (*tyyl yryata*), most probably related to the dysfunction of the PS circuit.

In traditional societies, PS lowers the standard of musicality to maximize singing accessibility. Close relatives sing their PS *for* aphoniacs. An indefinite intervallic structure and an absence of the notion of “wrong notes” make a PS performable by almost anyone. Playing JH is even more accessible.

The JH opposes PS by “de-personalizing” one's natural voice via the JH's vocoding mechanism. While camouflaging one's voice, the JH “impersonates” a wealth of environmental sounds. Its exceptional mimicry allows for re-creating the “real world” musically through the timbral abstraction of sounds and their arrangement according to principles of phonetic symbolism.

The JH addresses the objective aspects of reality, PS the subjective. Together, they form a coherent musical worldview, which explains why numerous North Asiatic ethnicities have so few “pitched” musical instruments: They simply did not have much need for them.

Indefinite-in-pitch rhythmo-timbral “themes” effectively represent:

- for PS—one's daily chores (“person”=monotheme, “chores”=variables in PS's variations);
- for JH—one's environment (“person”=low drone, “environment”=mid/high spectrum).

Both rest on the traditional hierarchic paradigm: “person”-“kin”-“ethnos”=“tree”-“tree-species”-“forest.” Such a paradigm must have formed in southeastern Siberia/Manchuria ≈10,000 BP. JHs made of ancestral plants were initially used as talismans. The accumulation of onomatopoeic devices and conventions of phonetic symbolism created “timbral words,” “timbral phrases,” instrumental patterns emulating PSs, and, eventually, proprietary JH TO, where the key role belongs to a set of “harmonic templates.” Each template carries a specific semantic value, enabling linguistic-like semiosis: meaningful elements comprise meaningful components, but convey emotional rather than referential information. This development must have directed the entire evolution of Eastern Eurasian timbre-based music systems in opposition to the frequency-based music of the Eurasian West^74^. *The JH was for the prehistoric East what the bone pipe was for the prehistoric West*.

The personal nature of PS/JH traditions stems from their reliance on timbre, which is fundamentally unsuitable for collective musicking. Collective performance of PSs or JHs would make their message unrecognizable. Only frequency-based music allows the tradition of collective performance to form and continue.

Western frequency tradition, exemplified by Aurignacian pipes (Morley, 2013), might have African roots. Africa underwent two major demographic expansions prior to the Aurignacian, enabled by its tropical ecology (Lahr and Foley, 2001). The second expansion (86,000–61,000 BP) carried haplogroup L3 outside of Africa (Atkinson et al., 2009). Genetic evidence suggests that non-Africans descend primarily from this migration, whose maximum falls on 70,000 BP, coinciding with the improved climate in East/Central Africa (Soares et al., 2012). At that time, the East African effective population size was at least 10,000 people (Relethford, 1998), vs. the census maximum of 3,700 in Gravettian Europe^75^.

Tropical environments generally support greater population densities than those at higher latitudes (Layton and O'Hara, 2012). Environmental conditions impact demographic density the most, and even the tropical desert supports a denser population than the polar biome (Tallavaara et al., 2018). Intergroup connectedness also drops at higher latitudes; the large effective population of connected groups in the African Middle Stone Age contrasts with stochastic variation without linear trajectories in the contemporaneous European Mediterranean region (Malinsky-Buller and Hovers, 2019). Sparse groups' migration leads to frequent losses of gained cultural skills. Steady post-Gravettian demographic growth triggered the cumulative cultural complexity that characterizes behavioral modernity (Shennan, 2001). Part of this was the consistent increase of foragers' group size (Grove, 2012). By 45,000 BP, the median effective population size in Europe equaled that which sub-Saharan Africa had reached 101,000 BP—alongside the markers of modern behavior (Powell et al., 2009).

Population growth promotes group cohesion, territoriality, ethnogenesis, and language formation (Robb, 1993). “Frequency music” likely followed suit. Steady demographic growth accompanied the Neolithic “revolution” and civilizations' rise (Hassan, 1981)—together with “frequency-based” music (Nikolsky, 2016). *Collective music-making was part of the “demographic expansion package” designed to consolidate and empower the “tribe” to grab and hold its territory*.

Radically different is the demography of northeastern Eurasia. Siberia is famous for its immense land (13,100,000 km^2^), sparse population (200,000 before Russian colonization), and the so-called everlasting importance of hunting/gathering for sustenance (Naumov, 2006). Such population density−0.065 person/km^2^–approximates the 0.036 person/km^2^ maximum of Magdalenian Europe (Maier, 2017). Harsh living in the traditional lifestyle makes landholding not a viable strategy. Constant migration by small “packs” requires marking and regulating the sharing of territory between all neighbors, helping each other to survive (Funk, 2006). Therefore, local beliefs assign power not to the “tribe” but to the spirit-masters of landmarks on whose disposition human “tenants” must rely. This has laid the foundation for the PS/JH pan-Siberian framework. And since the climate in Northeastern Asia remained remarkably stable throughout the Quaternary (Zamoruyev, 2004), it is reasonable to believe that the institution of PS/JH characterizes the music culture of local prehistoric people.

It can hardly be a coincidence that the area where JH remains to be the principal musical instrument in scarce instrumentarium is identical with the area of the greatest concentration of Denisovan genomes. The highest levels of Denisovan ancestry is found in Oceanic populations (Vernot et al., 2016). Denisovan genomes are also present in Eastern Eurasians and Native Americans (Qin and Stoneking, 2015). Denisovans may have interbred with early humans over the territory of Northern China (Martinón-Torres et al., 2017), where the oldest JHs were unearthed. Longevity of the JH dominance might constitute a distant remnant of the Denisovan timbral music tradition, preserved in the refugium of isolated Pacific islands, north-Chinese deserts and Altai mountains. Neanderthal heritage could entail the “frequency music,” carried from Africa by Homo Heidelbergensis. The latter adapted to the northern latitudes as opposed to southern Homo erectus—and so is the case with Neanderthals as opposed to Homo Heidelbergensis (Grove et al., 2012). Either of them might have adapted the southern collective “frequency music” to the northern ecosystems, generating a new^76^ personal “timbral music.”

The sparse Neanderthal (French, 2016) and Denisovan (Meyer et al., 2012) populations of the Pleistocene Altai (Buzhilova et al., 2017) might have also subscribed to “timbral music”. Homo's “timbral music” either descended from Neanderthals and Denisovans, or “downgraded” from the European “frequency music” carried by “Ancient North Siberians” from the West ≈38,000 BP (Sikora et al., 2019).

## Author Contributions

EA and AN conceived the presented idea. VD organized the recording of Jaw Harps at the Jaw Harp Museum in Yakutsk, collected all the necessary ethnographic materials and provided the most recent data of the fieldwork research in Siberia and Russian Far East. She verified the information on traditional musical instruments in Siberia. IA performed on different Jaw Harps and had them recorded and acted as a consultant in relation to the indigenous Siberian traditional music and matters of prosody and phonology of Jaw Harp playing and singing. EA provided his archive of the recordings and supervised the application of his methodology of the analysis of tonal organization of indigenous folk music. AN conducted the acoustic and musicological analysis of the provided material, conceived the method of analysis of spectral textures, corroborated all the findings with the research in the former USSR, modern Russia, and Western countries, wrote the manuscript and created the figures and tables for it. EA edited the manuscript. AN translated it into English.

### Conflict of Interest

AN was employed by company, Braavo Enterprises. The remaining authors declare that the research was conducted in the absence of any commercial or financial relationships that could be construed as a potential conflict of interest.

## Footnotes

^1^The emphasis in both quotations is added to the original.

^2^In case if the internet links provided for the audio examples end up being broken, the archive with all of the audio examples (named “Audio for the article”) is available in the Supplementary Material.

^3^Recently, considerable material has emerged, which well fits Teplov's theory. Timbre and pitch perception seem to engage the same brain resources, and therefore require selective attention (Melara and Marks, 1990a). Analytic extraction of pitch affects extraction of timbre properties, and vice versa (Melara and Marks, 1990b). Timbre affects judgements of simple pitch discrimination (Singh and Hirsh, 1992). Melodic motion can be compromised by timbral shift so that non-musicians could perceive a melodic tritone as larger in size than a perfect 5th (Russo and Thompson, 2005). Timbre can interfere with perception of melodic direction (Allen and Oxenham, 2014). Mistakes in melodic direction due to changes in timbre and loudness are especially pronounced in children as opposed to adults (Williams, 1990).

^4^The detrimental influence of usage of Western frequency-based music on timbral perception has been experimentally demonstrated in a number of studies. A tonal reference can reduce timbre's interference with pitch extraction (Warrier and Zatorre, 2002). This interference is much greater for Western non-musicians (less experienced in pitch analysis) than for musically trained listeners (Pitt, 1994). On the other hand, simplification and uniformity of spectral content seems to enhance frequency-interval discrimination in Western music (Zarate et al., 2013).

^5^Recently, this claim was substantiated by the evidence for two alternative pitch encoding mechanisms based on frequency resolution or non-resolution of harmonics of the fundamental tone (Grimault et al., 2002). This difference seems to lie at the heart of the distinction between “timbre-centered” and “frequency-centered” listening modes.

^6^Leontyev borrowed the concept of “functional organ” from Aleksey Ukhtomsky (1978) who held that “organ” did not necessarily have to constitute a morphologically distinct anatomical part, but may have constituted an organization of a specific function with *dynamic* rather than static attributes.

^7^Some support for Leontyev's and Zaporozhets' conclusion of post-natal formation of pitch discrimination skills comes from more recent Western research. Thus, McAdams and Bertoncini (1997) found that at birth infants can discriminate between rising and falling melodic contours only when they are presented with *timbrally and spatially contiguous sounds*. Schwarzer (1997) found that 5–7 year old children generally favor tracking loudness and timbre in analytic listening tasks over tracking melodic contour—unlike adult listeners.

^8^At present, cross-cultural investigation of general patterns of acquisition of music skills is in its virginal state. Only a handful of publications in English discuss the issue of musical development in non-Western societies (Blacking, 1967; Garfias, 1990; Fernald, 1992; Papoušek, 1996; Minks, 2002; Stige, 2002; Nettl, 2005).

^9^This generalization applies only to the indigenous folk traditions of timbre-based music – and not to the styles of Western classical music that are based on sonoristic effects rather than conventional “melodies” or “chords,” such as “sonorism” (e.g., Krzysztof Penderecki) or “spectralism” (e.g., Tristan Murail). Such styles generally tend to follow an “experimental” approach forged by Western modernistic composers through “inventing” their own original means of tonal organization rather than implementing already existing folk timbre-based music systems that have been “naturally” formed over a long period of time within an indigenous cultural tradition.

^10^The term “phono-instrument” was introduced by Sheikin (1996) to supplement the Sachs/Hornbostel structural classification of musical instruments by the classification of functionality of their use, where “phono-instruments” represent the archaic forms of musicking—preceding the invention of instruments designed specifically to generate a particular type of music (Sheikin, 2002).

^11^This lamellophone musical instrument is often called “Jew's harp,” which is somewhat confusing, since it is neither connected to Jews, nor to Judaism (Wright, 2015, XIV). A more appropriate name seems to be “jaw harp” (Crane, 1982), as suggested earlier by Sachs (1940, p. 58)—for the characteristic manner of its sound production. Player plucks the lamella while holding the instrument close to his/her mouth, used as a resonance chamber for amplifying the desired partials of the vibrating lamella (Ledang, 1972; Adkins, 1974; Trias, 2010).

^12^During the second half of the twentieth century, a number of blacksmith masters in the USSR, especially Yakutia, attempted to enhance the construction of JH by maximizing the instrument's dynamic capacities to make JH more suitable for concert stage performance (Dyakonova, 2011). Modernized instruments substantially differ from traditional ones—to such extent that some experts consider them “not simply an augmented khomus, but a new instrument in terms of acoustics and art” (Kunanbayeva, 1987).

^13^Personal communication with N. Mamcheva.

^14^The notable exception are those cases where making of an instrument has to pass through a specific magic ritual, such as divination of the tambourine for shamanic use, which might involve up to 9 specialists and take a few days.

^15^For instance, the choice of *ysyakh* (the summer solstice celebration) as a subject by a khomus-player usually involves performance of the *okhuokhai* rhythmo-formula (festive round dance), the *crescendo* effect (as if gradually approaching the festival), *kuoregei* (imitation of a larch-call, associated with sunrise), *kebe* (cuckoo call, announcing the onset of summer), *tabygyr* (associated with the sound of the horse trot and droplets of melting icicles), and *uyuu* (articulation of words representing shouting of excited people and singing of dance-leaders) (Shishigin, 1995; Alekseev, 1988).

^16^*Syyia* in Yakut literally means “slowly, staidly,” and *tardy*—“to yank, pull,” as to pluck the JH's lamella. *Syyia tardy* signifies a particular playing technique, where silent mouth articulations as though speak out syllables while the plucked lamella keeps vibrating. Its technique is also used in other styles that require increased clarity of articulation (Zhirkova, 1991). This underlines the priority of clear diction, precluding simultaneous engagement of many JHs.

^17^“Khomus” (“Khamys” in archaic spelling) is a Yakut Jaw Harp. The alternative meaning of this word is “Scirpus”—grass-like plant that grows next to rivers and lakes (Pekarsky, 1959, p. 3293). Most probably, the original instrument was manufactured of this grass—similar to the way modern Nivkhi and Ulchi make their grass JHs (Mamcheva, 2012, p. 50).

^18^JH duets occur either during dating or in joined entertainment between two female friends. Thus, in Bashkortostan, leisure sessions might engage up to 3 women playing together (Shchurov, 1995).

^19^One of the first accounts of such practice comes from the time of collectivization campaigns in Middle Asia after the establishment of the Communist rule. Vinogradov (1938, p. 88) describes the performance of a JH orchestra of Kyrghyz young women as a “haphazard juxtaposition” of multiple instruments “without any coordination,” where each performer played according to their own whims.

^20^The brightest features that distinguish this new use of JH from the autochthonous traditions of the past are its presentation from concert stage for a large audience, use of microphones and continuous concurrent playing of more than two instruments. Noteworthy is the globalization tendency of this new format—it attracts artists from many diverse ethnical backgrounds, including those that have not retained their autochthonous JH traditions (e.g., Russians).

^21^The empowering effect of collective frequency-centered performance is most evident in such genres as the work-song, military music and the national anthem—all called forth to increase group cohesion and to strengthen each of the participants.

^22^The propensity for self-communication – uniting self-reflection while performing, to the memory of oneself perceiving the same lyrical expression at some earlier time(s), was identified by Yury Lotman in regard to poetry (Lotman, 2001, p. 25). Musical expression can also follow suit. In fact, this is exceedingly common in traditional cultures of almost all indigenous people of Siberia (Sheikin, 2002, p. 304).

^23^This term originates from writings by Merriam (1964, p. 83). In English language literature, the term has been used in reference to “private songs” of North American Indians, e.g., Sioux (Austin, 1930), who, along with Canadian indigenous population, share the institute of PS with Siberian ethnoses (Ojamaa, 2002). In Russian research literature the most common equivalent term is “*lichnaya pesnia*.”

^24^The PS taboo can be illustrated by the following case: after a newly installed system in the settlement's club-house played back a PS recording of a recently deceased local old woman, the residents of Chukchi settlement in dismay demanded that its public broadcasting be stopped immediately (Novik, 1999).

^25^One often imitates a relative's PS when moving away from home, due to suffering from nostalgia. Reminiscing about a dear relative by singing a similar PS on a daily basis, then, serves as combating nostalgia.

^26^In the past, for many, if not most, Siberian ethnoses, multiple personal songs were common. However, during the communist rule the institute of PS was severely shaken by campaigns directed against “shamanism,” replacement of traditional lifestyles with modern ones, and the growing influence of “metropolitan” Russian culture, empowered through the education system. Thus, traditional taboo on non-personal use of PS was undermined by public broadcasting of those popular songs that are based on “personal motifs” of their creators, such as those by the distinguished Chukchi musician, Gennadii Pananto (Vensten-Tagrina, 2008).

^27^Russian ethnomusicologists often nickname PS a “musical passport.” Passport in USSR differed from Western passports in that it indicated one's permanent residential address (*propiska*)—in addition to identifying a person's face, name, and family status—very much what the PS usually indicates.

^28^The documented evidence often indeed details the description of cases where witnesses who had observed the AH behavior shortly developed the same or very similar symptoms themselves. Sometimes these symptoms did not disappear by time, but gradually exacerbated into regular fits. This was especially common amongst females.

^29^Thus, Yevdokiya Sergina, a professor of the Arctic State Institute of Art and Culture in Yakutsk, in a personal communication, admitted to have deliberately avoided contact with people that suffered from AH disorders in fear of their detrimental influence. To illustrate the potential dangers involved, she reported the suicide of a young researcher who specialized in shamanic mysteries, after having attempted to investigate such disorders.

^30^The modern reduction in severity and incidence of AH could also be explained by the growing acceptance of AH by the local population, who tend to avoid medical assistance—in combination with the lack of qualified scholars capable of establishing rapport with local population to obtain confidential information. Most likely, the exuberance of the anamnesis information in nineteenth to twentieth century Russia became possible because of the custom of condemning political prisoners to settle in Siberia. Most physicians and ethnographers who left accounts of AH were convicted to exile at first by Imperial and later by Bolshevik courts—a long-lasting tradition that was instituted from the times of Russian conquest of Siberia (Hartley, 2014). The length of their sentences secured their bond with the indigenous people. Additionally, political convicts of the nineteenth century (as well as Bolshevik officials appointed to investigate local living conditions) used to be atheistic—unsusceptible to fear of evil spirits.

^31^*Menerik yryata* singing that includes moaning, shouting and occasional clapping with lyrics as though coming in alternation from presumable “evil spirits” and the singer himself/herself, is still encountered not only amongst Yakuts but also their neighbors, who have their own names for such singing: *cármoriel* amongst Yukagirs, *haujan* amongst Evenki (Jochelson, 1910, p. 31).

^32^Shimkevich describes a traditional Nanai wooden idol (*seon*) and an iconographic image drawn on a piece of cloth, paper or wood (*girki*) of a deity (*burkhan*) that has multiple heads, representing the disarrayed and conflicting mentality (p. 49). Nanai call such multi-headed *burkhans* “*segemi*.” A similar tradition was described in neighboring Nivkh culture (Shrenk, 1903, p. 118).

^33^*Tyyn yryata* (“night songs,” in Yakut), or *tyyl yryata* (“sleeping songs”) is unconscious singing in sleep, often sung in foreign tongues that the singer does not speak when in an alerted state. This is still relatively common amongst Northern Siberian ethnoses, especially men. Evenks call this singing *náyani*, and Yukaghirs—*yendójenut yáxtei* (Jochelson, 1910, p. 13:37).

^34^To illustrate this point, in 1957, one of the authors of this paper, Ivan Alekseev, irretrievably damaged his vocal cords by the excessive singing of his PS in nostalgia, after having moved away from his homeland. Nevertheless, his “bad” voice in no way affected the public judgment of his PS singing. This is in stark contrast to “bad” singing of Western classical compositions, which requires sustaining the pitch without fluctuations, retaining pleasant timbre, etc. (Sundberg, 1987).

^35^Although these terms originate from Western musicology, they are applicable to non-Western timbre-based music too. PS users reserve certain attributes of the melody and the timbre to make PS recognizable across all modifications. This is quite similar to what classical composers do in stage works (operas, ballets) and program music (tone poems and overtures). In both cases, the retained “leit-nucleus” marks the features of the “protagonist,” whereas the changeable elements represent the “background” features, characterizing the context of action/happening.

^36^In Russian systematic musicology, the classic definition of “musical theme” is: “Musical idea that is fixed in musical structures, characterized by considerable completeness, salience, and originality—allowing one to recognize these structures upon repeated hearing—and that are often used continuously or reused within the same musical work” (Mazel, 1979, p. 151). Structurally, “thematicism” is the “totality of all themes employed in a work or in a group of works, which characterizes a particular music style (153). “Thematic material” is the complex of musical structures employed toward making a given musical work – disregarding their completeness and salience (152).

^37^The notion of “musical theme” is often described as peculiar to the Western classical music. This is not true. For instance, in South Indian Carnatic tradition, a musical composition is often segmented in 3 sections (*Pallavi, Anupallavi*, and *Caranam*) based on the treatment of the musical theme (Agrawal, 2018). The gamelan tradition not only utilizes the variation form, but also employs various forms of accompaniment to the principal theme (Miller and Williams, 2008).

^38^JH players choose a specific articulation (vowel or syllable), melodic intonation (a succession of pitches), imitation of a natural sound (birdcall) or a special technical device (staccato) for their specific expression. They use it either for repetition, variation, or contrast to some other thematic element – generating a sort of expressive “scenario” for a musical piece.

^39^Although Western listeners do integrate melodic and verbal information in lyrics prior to parsing song's lyrics, they process verbal and musical structures *separately* while listening to *familiar* songs (Sammler et al., 2010). In contrast, for indigenous Siberian listeners, autonomous processing of pitch and text does not seem to occur under *any* circumstances. Of course, final conclusion should be drawn only after experimental research of song's perception by representatives of well-preserved “timbral cultures,” such as the nomadic Nganasan or Evenk.

^40^In Siberia and Russian Far East, the exceptions from strict monophony are scarce: only the practice of collective repetition of the leader's verse by the chorus of dancers in Yakut *osuokhai* and similar dances of the neighboring peoples (Alekseyev, 1967), and, perhaps, the singing of the shaman's sidekicks during his *kamlaniye*—a ritual of reaching spirits necessary to fulfill the client's need (Dobzhanskaya, 2008).

^41^Thus, Khalkha Mongol women teach their children to play JH using imitations of horse and camel gaits. This is closely related to the herding family business in which children become involved at an early age (Pegg, 2001).

^42^Ivan Alekseev emphasizes that this generalization relies on the same synesthetic association of “smallness” with front vowels and “largeness” with back vowels that underlies phonetic symbolism of speech. JH articulations require constant control of all the constituents of the vocal apparatus, and reconfigurations of larynx and pharynx are usually done automatically—based on the reflexes established by speech.

^43^Other than having a peculiar timbre, each instrument is characterized by a specific pattern of sounds convenient for playing on it. Thus, mandolin is characterized by plucking tremolo, guitar by strumming chords, and cimbalom by hammering passages—each of which can be successfully imitated by other instruments (as in orchestrations of Sicilian, Spanish, or Hungarian style music). In musicological literature such characteristic patterns are usually put under the umbrella of “musical texture” (Frayonov, 1981). Most popular musical instruments have developed “glossaries” of such patterns that allow to tell a musical composition that was conceived for one instrument from another (Kholopova, 1979).

^44^For example, maidens of the Bontoc tribe sing inside their houses in response to young men's JH playing outside, trying to guess the identity of the player (Canave-Dioquino et al., 2008, p. 437). Amongst Li people of Hainan, in contrary, girls use JH to camouflage their voices in their confession of love to young men (Hsu, 2001).

^45^The capacity of JH to faithfully reproduce vowels and some consonants of a language is often employed to deliver words and phrases at the closest distance, so that the more remote occasional witnesses would not be able to eavesdrop on an intimate conversation. Thus, in Hmong tradition JH not only imitates linguistic phonemes, but also engages tone intonations (Poss, 2012).

^46^The extension of traditional beliefs in JH talismanic protection can be seen in the modern-day use of JH music for therapeutic purposes by the local Siberian population. Such is the program of “Meditative-imagery Jaw Harp therapy” at the Goriachii Kliuch Sanatorium in Krasnodar administrative district, where about 60% of patients report improvement from such problems as panic attacks, posttraumatic stress, acute depression, sleep disorders and irritable bowel (Kalinichenko and Alekseyev, 2009).

^47^Thus, Yenisei ethnicities consider JH to be the instrument of Kaigus'—a bear-like deity, master of all animals, who uses JH made of birch splinter to imitate voices of all animals and thereby exercises power to dictate to them (Sheikin, 2002, p. 126). According to myths, in order to secure prey, Kaigus' taught human hunters to play JH prior to hunting.

^48^The word “*seok*” in languages of Altaians literally means “kin,” “generation,” “bone,” and has been used in reference to a specific form of societal exogamic patrilineal organization (Verbitsky, 1865). *Seok*'s meaning of “kin” relates to its secondary meaning of “bone” through a more general meaning of “remains” (cemetery), where “bone” is understood as “the quintessence of live matter, capable of securing future births”—a trans-Siberian convention (Sagalayev and Oktiabr'skaya, 1990, p. 39). Hence, “bone” is something that is left from the deceased ancestor and that ties him/her with posterity: native Siberians believe that members of the same kin share the same bone ingredients (40).

^49^A stranger uttering names of a person, his relatives, sacred objects of his kin, including sacred trees, is believed to potentially harm that kin. This taboo has imposed heavy restrictions on wives, who despite bearing their husbands' children, remained estranged from their husbands' kin—thus, until the XX century, an Altaic widow did not receive inheritance after her husband's death (unlike her daughter) (Yenchinov, 2009).

^50^Video recording of M. Angin, the elder of the village Auri, Ulchi district, playing JH with the help of an axe can be seen in the documentary film “Ulchi,” made in 1967 by the Moscow Studio of Documentary Films (Duvan, 2003). This technique requires superimposition of axe's blade on the ring of a metallic JH. The same performance technique was also utilized by Nivkhs in the beginning of the twentieth century (Mamcheva, 2012, p. 54).

^51^Yakut master Piotr Krivoshapkin from the settlement, Khomustaakh (Namskii ulus), used to manufacture the JH-axe hybrid—which was filmed by a group of Czech filmmakers in 1967.

^52^Structurally, Even classification is based on the relation between music and words. *Tiinmei* (“to speak to oneself”) is an improvisatory song, created strictly for oneself, using timbral words mixed with spontaneous prosaic mundane texts. *Ikaan* (“song”) can be performed for others. It uses cliché fragments of poetic speech which, however, do not comprise poetic verses. *Alma* (“recollection”) is a “cover-song”—it reproduces someone else's words and/or musical themes and is retained as a ready-made “plot” in the memory of the following generations. *Alma* consists mostly of poetic speech.

^53^Chukchi classification is based on social grouping. *Chinitkin grep* conveys one's personal identity (“I, who sings my song”), *roiyr'yn grep* reflects one's membership in the family (“We, who share a home, sing as I do), *chygrymngyat grep* presents the entire clan of related families (“We, who share fire, sing songs to ancestors as I do) (Sheikin, 2018, p. 257, 41). The latter type has disappeared in the mid-twentieth century.

^54^JH personal instrumental pattern for dog-riding imitates barking with short tones articulated on syllables “*khai t'ok khai-nak-nak”* or “*khavr-khavr khaf-khaf-khaf* ,” while JH pattern for self-entertainment is based on sustained monotones on “*ko lyo-lyo-lyo*,” with vibrato of the lower lip or tip of the tongue.

^55^For example, all melodies of Ude songs include words “*yava-yava*,” whereas Nivkh songs—“*anga-anga*” (Sheikin, 2002, p. 250–1). Such words differ from “regular” meaningful words by being verbally meaningless and conveying semantic information through phonic symbolism alone. Often, they require a specific rhythm and melodic contour, thereby exercising a formative influence on the melody.

^56^Thus, in his diary from 1926 to 1927, Tchernetsov cites the meaningless sentence “*Gav-ri-ke, gav-ri-ke, vas pyrish, vas pyrish!*” that he heard continuously repeated on the JH (*tumran*) by a Northern Mansi girl in a remote Sos'va settlement.

^57^For example, a *syyia tardy* can be dedicated to such subject as representation of a young man and can present an “aesthetic” contemplation on such a “topic.” JH player envisages a protagonist (here, a young man) and fantasizes a storyline, more or less cohesive and tangible, that is not always describable in words. Such a composition is “about someone else” (“objective”)—as opposed to the typical PS which is “about the singer” himself/herself (“subjective”).

^58^We must underline that the homogeneous nature of singing manifests itself *only* in frequency-based musical traditions. Vocalizations of timbre-based cultures, including PS and vocal “games,” such as Inuit *katajjaq* or Ainu *rekkukara* (Nattiez, 1983), abide by principles of timbral contrast, and are considerably richer than conventional frequency-based vocal music. Such timbrally-rich vocalization styles are used by Chukchi, Nganasan, Koryak, Kerek, Even, Enets, Yukagir, Nanai, Oroch, and Ude (Sheikin, 1996). As Sheikin demonstrates in his analysis of these styles, their TO is based on the binary opposition of two “timbral classes”: inspiratory and expiratory rasping, each of which is varied by employing different syllables and intonations. There can be as many of 20 “timbral classes” (e.g., the *pilche'ingen* vocal system of Chukchi).

^59^That is why multiple attempts to modernize the traditional JH constructions by adding extra lamellas or joining a few JHs on a single “JH farm” did not really catch up with majority of JH players. Western European players of multi-pitched JHs subscribe to the model of Western tonality borrowed from classical music. Chinese players follow the model of traditional Chinese music system based on *yayue*. Like Western tonality, this constitutes a type of **tonality**—frequency-based music (as opposed to timbre-based indigenous JH traditions).

^60^This matter is thoroughly examined in the acoustic and ethnographic study that is scheduled for publication by the state People of the World Khomus Museum and Center in Yakutsk, Russia next year.

^61^In Yakut language exclamation “*urui*,” especially common for shamanic verses, is used in prayers for well-being, repelling evil, expressing a jolly feeling, calling for fighting, or announcing an end to something (Pekarsky, 1959, p. 3070).

^62^She distinguished between 4 textural layers in the repertoire of Chuvan JH music for frame-shaped JHs: (1) the FF; (2) its subharmonic chest resonance an octave below; (3) the middle melodic layer above FF controlled by the player's breathing and tongue position, exhibiting “talking” qualities; and (4) a high melodic layer that changes the sustained phonemic formants which resembles singing.

^63^There is experimental evidence that non-musicians can simultaneously track no more than 3 concurrently running polyphonic parts, and trained musicians—up to 4 (Stoter et al., 2013). Although this was established in relation to frequency-based music, it can also apply to timbral-based music since most known traditions of vocal and instrumental timbral music too use no more than 3–4 parts. It is reasonable to expect the JH players to consistently and reliably control no more than 2–3 melodic parts. This assumption agrees with the anecdotal information from JH players.

^64^Sheikin speaks of the “bamboo period,” but his choice of the term “period” is not the most accurate for designating the timeline of JH's evolution. The word “period” implies a “hard” end—one period replacing another. However, none of the JH materials have been really “discontinued” in the production of JH. Once mastered, a material usually remains in use. Therefore, the term “stage” seems more appropriate here, since developmental stages are cumulative in nature and built on top of one another.

^65^In Taiwan and Indonesia there are very few examples of indigenous musical traditions that use bamboo frame-shaped JHs with metallic lamella (Kolltveit, 2016). This is also the case in Vietnam (Wright, 2001). However, judging by available recordings of such instruments, this hybrid construction does not substantially differ in spectral texture from the frame-shaped instruments that are made just out of bamboo. On the territory of Russia, bow-shaped JHs are always metallic (Sheikin, 2002, p. 129).

^66^There are some vague ethnographical references indicating that wooden JHs have been used in Flanders, Hungary and Ireland—however, such cases seem to be exceptional, leaving no archaeological or historic traces (Kolltveit, 2009). It is likely that wooden instruments such as Slovenian *drumlja* present a relatively modern development, called to reduce costs and thereby increase sales of JH in modern market conditions.

^67^Low quality of mass produced instruments is known to negatively affect the standards of JH music produced on imported JHs—as compared to hand-crafted instruments of indigenous JH traditions (Morgan, 2017). Metallic instruments, less responsive to the player's technique, must have further limited the spectral content of JH texture, increasing the dominance of a single melodic “part” even more.

^68^Such scenario has been confirmed in relation to Yakutia where organic frame-shaped JHs were found to precede the metallic bow-shaped JHs (Dyakonova, 2017). The same applies to the peoples of Volga-Urals (Yakovlev, 2001), Kama (Golubkova and Ivanov, 1997), Sakhalin (Mamcheva, 2012).

^69^Thus, amongst Khanty and Mansi, frame-shaped *tumran* is strictly female, related to wedding rituals of fortune-telling and “avoiding” men (Sheikin, 2002, p. 127). Across Southeastern Siberia bow-shaped JHs are used exclusively by men in funeral lamentations, whereas females use bowed pikolute instead (131).

^70^An interesting case presents the tradition of Ude JH. Despite the fact that Ude hunters consider frame-shaped JHs as being children instruments, nevertheless, they report that hunters must play it after setting their hunting traps to ensure that the prey will be trapped (132). This testifies to a more ancient origin of the frame-shaped construction in hunter-gatherer's societies—in contrast to the bow-shaped construction that did not exist before the Metal Age. The same applies to their respective musical textures.

^71^JHs, rather popular amongst modern Inuits (Nattiez, 1976), have been introduced in Alaska, Canada and Greenland from Western Europe (Whitridge, 2015). The JH-like use of the feather of an arctic bird, common eider (Somateria mollissima), was observed amongst the Inuits of the Belcher Islands and northern Quebec (Oakes, 1991). They hold the feather in their mouths and beat it with one hand. A photograph of this is published in the LP “Inuit Throat And Harp Songs” (Green and Hodge, 1980). Most likely, feather is used as a more affordable material to substitute the imported metallic JH. However, the possibility that the feather use is a local invention that might have preceded JH trade cannot be completely discarded. However, even in this case, such feather use remains totally isolated.

^72^Thus, three of the authors of this paper were raised in the native cultural environment of Yakutia, and therefore combine a first-hand “emic” knowledge about its indigenous music (and language) with an expertise in musicological analysis of such music.

^73^Long-term memory for melodic structures is known for remarkable longevity and capacity (Halpern and Bartlett, 2010). Accurate memorization of music can be life-long (Bartlett and Snelus, 1980). Music seems to provide superior encoding, evident in the phenomenon of earworms, unique to the domain of music (Halpern and Bartlett, 2011).

^74^For more information on the role of flute-like instruments in determining a music system see “Tonal organization in tuning of Paleolithic and Neolithic pipes” (Nikolsky, 2015, Appendix-2).

^75^The median ratio of effective population size to census size remains exceedingly low across 43 mammalian species, constituting on average 0.003 (Nei and Graur, 1984). The method of mismatch analysis, introduced by Rogers and Harpending (1992), allows for estimation of size and timing of ancient demographic based on the genetic data. Population growth at the time of expansion is estimated to be 100-fold or even greater (Rogers, 1997). And the timing of it clusters around 50,000 BP (Sherry et al., 1994). The African expansion of this time suggests a demographic picture with sparse population outside of Africa, while a rapid population explosion inside of Africa (Relethford, 1998).

^76^If the Divje Babe pipe is indeed not a byproduct of the hyena bite (Diedrich, 2015), but rather a Neanderthal musical instrument (Turk, 2014), then the Neanderthal expansion to Altai would have created a Paleolithic border between the frequency- and timbre-based music.

## Supplementary Material

Data Sheet 1 Appendix. Spectral texture.

Data Sheet 2 List of audio examples.

Data Sheet 3 Textural typology for Appendix.

Presentation 1 Audio for the article.

Presentation 2 Audio for Appendix.

Presentation 3 Figures for Appendix.

## Abbreviations

PS: personal song
JH: jaw harp
AH: arctic hysteria
TO: tonal organization
FF: fundamental frequency
f1: first harmonic
F1: first formant
dBFS: decibels relative to full scale
BP: before present.
