# Supplementary material for: The Overlooked Tradition of “Personal Music” and Its Place in the Evolution of Music
Source: Front Psychol. 2020 Feb 18;10:3051. doi: 10.3389/fpsyg.2019.03051 (PMC7040865; doi:10.3389/fpsyg.2019.03051)
Supplement: Data Sheet 1 — Appendix. Spectral texture. [file Data_Sheet_1.pdf]

Appendix to "The overlooked tradition of 'personal music' and its place in the evolution of music."

Spectral texture and the influence different materials have on it on the example of Jaw Harp

Abstract

This paper reveals the connection between the acoustic properties of different materials from which Jaw Harps are made, and the musical properties of Jaw Harp music. To capture and classify these typological differences the paper proposes a new theory of "spectral texture" and "spectral parts" as constituents of "spectral texture." This theory can be applied not only to Jaw Harp music, but to other forms of timbre-oriented music as well. The samples of traditional Jaw Harp music performed on instruments made of 10 materials, most commonly used for manufacturing of Jaw Harps (4 organic, and 6 metallic), were analyzed using the proposed methodology. As a result, 14 types of texture were identified. The simplest of them (3 types) engage 3 parts and are strictly homophonic. They are encountered only on metallic heteroglot instruments. The most complex types (4) engage 5 parts and are mostly polyphonic (3). They are generated mostly on the instruments made of organic materials (3) or idioglot brass (1). All metallic materials form a well-defined and archaeologically supported timeline for when they were introduced, which is trans-cultural (the order of their succession is retained in majority of known cultures). The organic materials lack reliable archaeological evidence that could have established their dating. Nevertheless, the order of mastering of the technology of their manufacturing by humanity can be inferred based on availability of the raw material, the complexity of manufacturing, the necessity to use tools and the time that it typically takes to make a Jaw Harp from a particular material.

Based on the totality of available information, all 10 materials can be ordered in a timeline, which reveals the progression of increasing simplification and functional differentiation of spectral textures, accompanied by the transition from exclusively polyphonic to predominantly homophonic arrangement. Such distinct association of organic materials with polyphony and complexity - versus metallic materials, associated with homophony and simplicity - must be responsible for parallel coexistence of idioglot (mostly organic) and heteroglot (almost exclusively metallic materials) traditions of Jaw Harp music across Siberia and Far East. Introduction of newer "metallic" tradition there most likely caused the already existing more ancient "organic" tradition to retreat into a different cultural niche. Such capacity to conserve an older technology alongside the associated spectral textural type, complicates the entire perspective of textural typology of Jaw Harp music. Nonetheless, the undoubtful link between the mechanical properties of different materials and the musical properties of Jaw Harp musicking must have determined the overall transition from polyphonic to homophonic musical thinking. Moreover, the growing popularity of using metals for manufacturing musical instruments that is evident in most, if not all, musical cultures of the world - since the Bronze Age - suggests that the general transition from polyphony to homophony, known in numerous cultures (especially clear in Western classical music tradition) should be attributed to the increasing dominance of metallic musical instruments over those made of organic materials.

Table of Contents

1. *JH thematic material in light of the general theory of musical texture* 2
2. *Towards the typology of "parts" and their respective tonal "modes" in JH music* 4
3. *Prolegomena of the methodology for textural analysis of JH sound recordings* 6
4. *Grass idioglot JH: 5-part polyphony* 10
5. *Bamboo idioglot JH: 4-part polyphony* 11
6. *Wood idioglot JH: 4/5-voice polyphony* 13
7. *Bone idioglot JH: 4-part polyphony* 14
8. *The comparison of wood, bamboo and bone* 16
9. *Brass idioglot JH: 4/5-part polyphony* 17
10. *Copper heteroglot JH: 4-part polyphony* 18
11. *Bronze heteroglot JH: 4-part polyphony/3-part homophony* 19
12. *Brass heteroglot JH: 4-part homophony* 21
13. *Iron heteroglot JH: 4-part polyphony/3-part homophony* 21
14. *Steel heteroglot JH: 3-part homophony* 23
15. *Stainless steel heteroglot JH: 3-part homophony* 25

16. *The comparison of copper, bronze, brass, iron and steel.* *26*
17. *The summary of historic succession of the introduction of JH materials* *29*
18. *How the mechanical properties of JH made of different materials correspond to their respective textures* *33*

Since most performers of Jaw harp (JH) authentic style music cannot conduct acoustic and musicological analysis of the music they produce, and the "musical ear" of scholars who are capable of such analysis is usually developed on "frequency-based" music, it is necessary to adopt a reliable methodology of comparative analysis of different samples of JH music. To base a study on ethnographic data (collected from JH users) alone precludes a comparative approach, since different JH traditions pay attention to (and/or ignore) certain aspects of musical organization that are objectively present in sound differently from each other. Therefore, emic information can only be used in conjunction with etic data. This data can be derived from 3 sources:

1) spectral analysis based on cross-examining the data, collected by software frequency analysis of audio samples,
2) visual comparative analysis of spectrograms, and
3) comparative audition of the investigated samples.

The already existing framework for comparative musicological analysis of musical texture can be used to capture the characteristic traits of spectral context for every recorded sample of JH music, and to categorize them by their similarities and differences in distribution of the *thematic material* from the lowest to the highest bands within the human hearing range.

1. JH thematic material in light of the general theory of musical texture

The criterion for determining the "thematic material" will be the continuity of the changes in frequency/time/intensity within a specific frequency band (=*register* of the JH tessitura) as manifested in relation to the adjacent higher and lower bands – provided they are salient enough to be distinguished by ear during audition. The shortcoming of this definition is its reliance on the ability to hear a particular component in the spectrum. Such ability substantially varies between different listeners, depending on their acuity of hearing, experience and attention. Nevertheless, this criterion might still present a good starting point for collecting data and identifying the most typical thematicism for JH. As the experimental research unveils the average thresholds of sensitivity to changes in registral components of the JH music (preferably, for JH players and non-players), it will be possible to set hard limits for definition of JH thematic material – disregarding audition – based on acoustic measurements alone.

The term "*musical texture*" is rather vague, significantly varying in its exact meaning within different contexts. Thus, the Grove Dictionary does not even offer a concise definition – only stating that texture "refers to the sound aspects of a musical structure" (Anonymous 2001). Part of the reason for this is the relative novelty of the concept of "musical texture" – it was formed as a specific theoretic notion in Western Europe no earlier than 1930s, introduced and developed only in English, and not German, French or Italian musicology (Dunsby 1989). In English musicology this concept was formulated in 1938 to reflect the historic changes in the typology of compositional techniques of counterpoint in European polyphonic music (Tovey 1941). In Russian musicology the concept of texture was introduced 3 years earlier, in relation to the typology of compositional techniques of harmony in European homophonic music – under the term "tip izlozheniya" (presentation type) (Sposobin, Yevseyev, and Dubovsky 1935). Two years later, this notion was renamed into "texture" by Yurii Tiulin (Tiulin 1937, 31–36), and was generalized as the universal feature of compositional arrangement, present in any form of art music that is based on Western theory of harmony. German musicology, famous for its lead in many areas of music theory, for some reason did not develop its own theory of music texture – only borrowing this term from English as "textur" in the 1960s (Kholopova 1979, 4).

Initially, in both, English and Russian, the meaning of the word texture was understood as the "vertical" aspect of organization of music by its parts and/or voices. However, in Russian literature it quickly attracted the attention of numerous music theorists and was employed in the state system of general elementary music education as part

of its curriculum (Kudriavtsev and Taranuschenko 1956), in specialized elementary music education (Ostrovsky 1976), and in college level course of introductory musical theory (B. Alekseyev and Miasoyedov 1986). As a result, the concept of "texture" became a common descriptor for specificities of musical arrangement – as common a term as music form or harmony. Subsequently, in Russia the domain of application for "musical texture" kept expanding. It incorporated monophonic music, including instruments capable of playing only one pitch at a time (e.g., flute solo) as well as solo vocal – where "texture" was understood as a particular type of patterning a melodic line by means of changes in melodic contour, rhythm and register (Skrebkov 1973, 136). Textural typology was discovered in traditional forms of folk music, causing expansion of musical texture theory to include heterophonic textures (Dmitriyev 1962). Viktor Tzukkerman extended textural typologies to cover orchestration techniques (Tzukkerman 1975). He and Leo Mazel, another renowned Russian music theorist, together, identified the contribution of textural changes within a musical work to the shaping of its music form – making textural analysis a required part of the analysis of music form (Mazel and Tzukkerman 1967, 331–42), which was adopted by graduate schools of musicology across the countries of the former USSR. The semiotic aspect of musical textures was revised by Yevgeny Nazaikinsky (Nazaikinsky 1982, 70–149). The "globalized" historic perspective on the development of musical texture in Western tradition was captured in two monographs: by Valentina Kholopova (Kholopova 1979) and Marina Skrebkova-Filatova (Skrebkova-Filatova 1985).

In English literature, "musical texture" was also expanded into a general theory. Walter Piston identified its orchestral typologies (Piston 1955, 355–414). Its contribution to compositional techniques was investigated in relation to polyphony (Green and Jones 2015), homophony (Levy 1982) and the transitional polyphonic-homophonic styles (Comberiati 1983). Leonard Ratner summarized the theory of music texture for variety of genres and styles of Western classical tradition (Ratner 1980, 108–80), and Wallace Berry outlined its contribution to the typology of music form (Berry 1987, 184–300).

If to follow its extended definition by the Russian Musical Encyclopedia, there is no reason to reject the application of the term "music texture" to JH music. This definition explains music texture as an aspect of music form that relates to a specific arrangement in pitch/time of all sounds engaged in a music work or in its autonomous portion – to put it simply, as being a "type of presentation" of music (Frayonov 1981). Although this definition was formulated in relation to "frequency-based" forms of music, nothing prevents its application to "timbre-based" music, provided the aspects of "pitch/time" are replaced with "spectrum/time." Not only that such substitution is theoretically possible, it is in fact necessary in order to keep the uniformity of JH study with that of vocal forms of timbral music, where this substitution has already been made by ethnomusicologists.

In fact, JH music is closely related to Tuvan (Aksyonov 1964; Vargyas 1968, 71), Bashkir (Garcia 1847, 24–25; Rybakov 1897, 2:270–72; Ikhtisamov 1988), Khakass and Khazkh (Tongeren 2004, 126-7), Mongolian (Hamayon and Helffer 1973; Gunji 1980), as well as Chinese Inner Mongolian (Pegg 1992) traditions of deep throat singing. It is not by accident that all these regions are characterized by great popularity of such singing - as well as JH playing. And in Russian ethnomusicology such form of singing, the drone tone simultaneously with the melodic line comprised of overtones, is commonly referred to by the paradoxical term, "vocal *solo polyphony*." Evidently, the concept of musical texture well suits this technique of singing: one singer as though produces 2 parts of music at once, not unlike the choir performing, for example, of Byzantine chant. Moreover, just as the drone choral singing can engage different textures (e.g., in low vs. high part, or mono-rhythmic vs. bi-rhythmic), different traditions of "solo polyphony" are also distinguished by their textures. Thus, Tuvan *borbangnadyr* and *ezenggileer* differ exactly in their choice of texture (Levin and Suzukei 2006, 66) .

The same applies to JH music – especially, if to take into consideration that in Bashkir traditional music playing kubyz (JH) and singing *uzliau* (solo polyphony) exists side by side with the tradition of simultaneously playing *kurai* (flute) while singing a drone (that occasionally shifts) by the very same performer, which effectively generates 2 discrete parts (Ikhtisamov 1988). And the fact that JH can produce more than two "parts" does not change much, because "solo polyphonic" singing can also produce three components (Ikhtisamov 1988).

Essentially, a "part" of JH texture is comprised of the progressive changes in relative pitch level of a particular vowel **formant** throughout a continuous segment of music. Tracking the same formant between contiguous JH tones effectively creates "formant voicing" - following the model of voice-leading within a chorale musical

texture. The justification for layering the overall spectrum of the Jew's harp phrases into "formant voices" comes from the inherent property of music to inter-relate adjacent tones in pitch, which applies not only to a simple case of discrete tones in a "tune," but to voicing within any complex entity, be it multi-part polyphonic texture or a homophonic progression of chords. Just as a discrete tone can be detected in a chord, a salient partial can be detected in a complex tone. Therefore, it is plausible to expect that "voicing" of two registrally proximal tones in adjacent chords within the same musical phrase finds its analog in registrally proximal formants of neighboring inter-phrasal complex tones. A particular case of such "partial voicing" is well-known in the practice of playing popular tunes on a Jew's harp and singing such tunes in Tuvan and Mongolian styles of throat-singing. And voicing of partials is not limited to the principal melodic line either. The sustained fundamental tone is perceived by Jew's harp players and throat-singers as a discrete "voice" that can be continued, interrupted or varied.

In light of all this, it seems justified to assume that "voicing" constitutes one of the rules in making timbral music and listening to it within its authentic culture. The present paper adopts a "voicing model" for cross-examination of typical musical textures produced on Jew's harps made of different materials, in order to identify the contribution of a specific material to the acoustic properties of the music produced on such musical instrument.

2. Towards the typology of "parts" and their respective tonal "modes" in JH music

Since JH music always contains at least 2 components, it should be considered essentially "polyphonic." Although the concept of musical texture has been traditionally devised for categorization of polyphonic music throughout the course of its development in Western Europe in XII-XVIII centuries, it was later extended to apply to non-Western types of music, including folk traditions. According to the consensus of world's ethnomusicologists, polyphony is "a mode of expression based on simultaneous combination of separate parts, perceived and produced intentionally in their mutual differentiation, in a given formal order" (Agamennone 1996). Clear distinction between the constantly sustained drone and the constantly moving melodic line in all cases of JH's music satisfies the definition above. This is because the opposition of changeability and permanence, simply unavoidable for the performer, constitutes the "formal order," immediately noticeable to the listener's ear. The only complication of timbre-based music that calls for extension of the abovementioned definition is that the "drone" and the "melodic line," in JH music, are not necessarily comprised of "pitches." Being "pointillistic" –a characteristic of modern Western style of playing JH, common in such countries as Austria and Germany – constitutes the exception rather than the rule. More often than not, in European indigenous and non-European traditions, "drone" and "melodic line" are made of not "points" of pitch but rather of "blot-like" spectral component. Drone usually consists of a few harmonics: most commonly, f3, f4, f5 and/or f6, but possibly, including f2 and f7 – plus frequent use of the sub-harmonic an octave below f1, characteristic for many Siberian traditions. Melodic line also can be thick - encompassing a few harmonics and/or noise components or inharmonic (in relation to the FF) partials. Therefore, it would be inaccurate to refer to the changes in JH melodic line as "pitches." It seems more appropriate to call them "degrees" of a "timbral mode."

The term "mode" here is understood in its most general sense, as formulated by Viktor Beliayev: "mode is the generalization of *types of melodic motion* in relation to *intervallic structure* of these types" (Beliayev 1990).[1] The only specification that should be added here is that "interval" in the context of timbre-based music should be understood as "indefinite in pitch" – ekmelic (Nikolsky 2015). Its exact size can fluctuate within certain margins, depending on the extent of the performer's excitement or relaxation. Such indefinite scalable (ekmelic) intervals are not "quantitative" – as those categorized by Western music theory (intervals of 2nd, 3rd, etc.) – but rather "qualitative." They are distinguished by the extent of melodic motion:

- keeping more or less the same position (qualitative "unison" that can be slightly higher/lower)
- stepping close by (qualitative "step" above/below)

---

[1] For a brief summary of the standard Russian approach to the modal analysis of music see the appendix "Taxonomy of modal music" (Nikolsky 2015, Appendix-1).

- leaping aside (qualitative “jump” above/below).

The distinction between a step and a jump here is purely psychophysiological – based on the “temporal coherence boundary” that determines the segregation of audio stream (Noorden 1975). If to express this boundary in a quantitative manner, it would fall on the interval of a minor 3rd (circa 300 cents) for music in slow tempo, and a 4th (400 cents) for fast music (Huron 2001).[2] According to the ethnographic data, users of timbre-based music do distinguish between smooth melodic motion (steps) and abrupt changes (jumps) – perceiving the former as “agreeable” and the latter as “disruptive” (E.Alekseyev 1976).

It is possible that both, jumping and stepping, might include further gradations in quality (e.g., longer leaping vs. shorter hopping for “jumps,” and shorter “mincing” and longer “stamping” for “steps”). The argument for this comes from the necessity to distinguish between two melodic lines. In their JH textures, majority of Siberian and Far Eastern traditions utilize at least one more melodic line that usually moves concurrently with the primary one. Sometimes they are equal in every respect, sometimes they noticeably differ in amplitude, number of “degrees” in their melody, their concision (whether a degree consists of one or more harmonics) and/or their tonal relationship. The latter depends on the registral position of the melodic line in relation to the harmonic series of the FF to which the JH is tuned.

- The melodic motion between the lowest harmonics (f3-f7) comprises “arpeggio” – a scale that contains no 2nds. This could constitute a narrower “hopping” as opposed to wide “leaping” by 5th or octave.
- The motion between the medium low range harmonics (f6-f11) makes an anhemitonic scale (containing no semitones). This could constitute the largest mode of “stepping” – “stamping.”
- The motion in the medium high range (c. f9-f15) constitutes a hemitonic scale (containing whole tones, as well as semitones). This could constitute medium size “stepping.”
- The motion in the high range (f14-f25) generates a chromatic scale (semitones only). This could constitute small size “mincing.”
- And the motion in the highest range (>f25) produces microchromatic scale. Although perception of michrochromatic intervals remains a virgin land – especially in timbre-based music – it would be premature to discard the possibility that JH players can detect microchromatic melodic changes.

Of course, it remains an open question as to how well (if at all) the users of timbre-based music can distinguish between melodies based on each of these 5 types of tonal relationships. For musicians raised on frequency-based music this task presents no difficulty at all – after the completion of the course of ear training, required in Russian colleges, majority of students have little difficulty identifying specific hemitonic scales (e.g., Lydian, Dorian, etc.), which is much harder than to distinguish between whole classes of scales (e.g., anhemitonic vs. hemitonic). Unfortunately, there has been no experimental research dedicated to the ability of non-musicians to distinguish between melodies created in each of these intervallic types – apart from the simplest cases of distinction between major and minor scales. The latter was found too difficult for majority of listeners without any musical training (Leaver and Halpern 2004; Halpern, Martin, and Reed 2008; Vos and Verkaart 1999). However, once again, we have to emphasize that subjects in such experimental studies are overwhelmingly users of “frequency-based” music. “Timbre-based” musicians might possess different sensitivity to the tonal properties of melodic line – which might be significantly higher than that of “passive” users of frequency-based music. After all, most members of traditional Siberian societies actively use timbre-based music, which puts them in the same category as “musicians” in Western cultures – who typically exceed “non-musicians” in acuity of their musical hearing. According to the testimonies of performers of authentic “timbre-based music,” their ear is much more sensitive to the smallest fluctuations of timbral qualities than ear of listeners brought up on “frequency-based music.” In working with them to produce samples of different JH textures, it became obvious that their low range hearing extends much lower than normal – down to 10 Hz. And they seem to quite reliably identify each of the vowel

[2] Of course, the ultimate confirmation for this assumption should come from experimental research, since the temporal coherence boundary was established for the “frequency-based” music.

articulations while listening to the recordings of their own performance. Nonetheless, their ability to distinguish between melodies created in different intervallic types remains to be studied.

3. Prolegomena of the methodology for textural analysis of JH sound recordings

It seems plausible to expect the same principles that are at play for perception of polyphonic textures to remain valid in regards to the perception of JH textures. Current experimental research indicates that for listeners without musical training the maximal number of adequately identified parts is no more than three. This includes timbrally homogenous (Huron 1989), inhomogenous (Stoter et al. 2013) classical music, as well as Western popular music (Schoeffler et al. 2013). Musical training increases the number to 4 parts (Stoter et al. 2013). This limit agrees with the convention of composition, observed by Western composers from Baroque times on. Even when the number of parts in a polyphonic work exceeded 4, composers follow the custom of keeping no more than 4 melodically "active" parts at any given point in time – only shifting them across the texture. Noteworthy, most textbooks on polyphony focus on double and triple counterpoint, so as most instructional polyphonic pieces (e.g., inventions by J.S. Bach). In the ear-training methodological literature, such as dictations polyphonic training is also limited to 2- and 3-part exercises – 4-part exercises do not use polyphonic textures and abide by the strict rules of classical harmony (which allows for writing such dictations not so much by "ear" per se, but by rules of chorale writing), while 5-part exercises are non-existent. Homophonic textures, with all the wealth of their typology, are still easily reduced to 3(4) active parts – with the only principal difference from polyphonic textures where a part might contain chord or double-note progressions, thereby distinguishing not only between parts, but also between voices within a part (Kholopova 2002). The underlying mechanism for this reduction most likely is determined by listener's attention that tends to focus on marginal registral parts, which are usually most salient – then, whatever is going on between the bass and the treble part is treated as a "filler" (Nazaikinsky 1972, 118).

It is not by accident that most instructional treatises on composition starting from the 16th century, when composition became regarded as a "practical" occupation, rationally related to commercial success rather than "religious" practice of adherence to scholastic rules, promote at first the method of drafting a 2-part counterpoint between the primary melodic part and its most salient "opponent", and then "filling up" the rest of the parts with the neutral material according to the "norms" of the music theory. The practice of general bass further promoted the "margin-based" thinking, where bass and treble parts received the greatest prominence – especially the upper melody that became the staple of expressing the composer's point. The rest of the parts/voices were left to the rudiments of harmonic theory. When homophony became established as the prevalent style of music, this approach transformed into the overwhelmingly popular "diagonal" method, where only 3 parts (melody, bass, and one of the accompanying voices in the middle of a texture) would run continuously throughout the entire composition, and the normative position for melody became in the uppermost part. It would not be an exaggeration to state that 3(4)-part polyphonic, 4-voice homophonic, and 3(4)-component polyphonic-homophonic textures do constitute the standard for most art works throughout the 18th - mid-20th century practice of Western composition. Psychoacoustic research indicates that this "contour" framework of the "marginal" melody-bass counterpoint with the "filling" in-between is more than a cultural phenomenon – it might have psycho-physiological roots. Although listeners divide their attention between all the identified parts (Demany and Semal 2013), the higher part receives better encoding amongst musicians as well as non-musicians, and even years of playing a low-range instrument does not reverse this bias (Trainor et al. 2014). JH music is likely to follow the same principle of "contour polyphony" between the uppermost and the lowermost melodies. There is evidence that harmonic expectations make the melodic content of the constituent parts of texture harder to detect, especially for its middle parts (Palmer and Holleran 1994). Then, JH textures, free of any influence of harmonic tonality, would be easier to break into parts than textures of Western multi-part music.

Following this brief outline of music texture, we can now lay out the general procedure for analysis of JH textures.

1) We start by detecting the fundamental tone amongst majority of JH tones in a musical phrase. This can be accomplished through audition along with the examination of the spectrogram in search of the component that is the least changeable over time and frequency. Defining the prevalent component with more or less permanent frequency value throughout the entire music would establish the "bass" part. In majority of

cases this is going to constitute a strict "pedal" – a sustained single "pitch" (FF), a "double-pitch" made of 2 harmonics, adjacent in their position in the harmonic series (e.g., octave of FF/f2, 5th of f2/f3, or 4th of f3/f4) or a "chord" made of 3 or 4 adjacent harmonics (triadic, as in f2/f3/f4/f5, or 7th-chord based in f4/f5/f6/f7). However, we should keep in mind that occasionally JH "bass" can change in frequency (e.g., in JHs made of grass or in JHs with multiple lamellas).

2) The next step would be to define the primary melodic line. Again, this is best achieved by auditioning a sizeable excerpt of music in cross-reference to observing its spectrogram in real time. The latter will identify the most intense frequency band within each of the JH complex tones. Then the identified components should be related to one another between all consecutive JH tones of each musical phrase – in reference to their registral position within the compass of JH. If changes stay within a relatively narrow frequency range (about an octave), its bandwidth marks the register for the melodic part. Typically, the principal melody is designed to contrast the pedal bass – the least changeable component of texture - by featuring the greatest number of changes: in pitch (number of degrees used, of changes in direction, and of alternation of steps and jumps), rhythm (number of pitch changes for each tone) and register range (the principal melodic part usually has the widest range amongst all other part). In essence, the task of finding the main melody here is the same as finding a melody in a Western harmonic chorale, where it can be placed in bass or tenor. This task requires defining the "working tessitura" for each part and comparing the thematic material for each – a choral melody would feature the greatest diversity of pitch changes, placed at metrically strong time, contains numerous jumps, and exhibits melodic patterns. The same would apply to JH texture.

3) Once the principal melody is established, we can find out if the texture contains another melodic part. Visual identification in a spectrogram of other dynamically salient components in each complex tone, different from the principal melody and carried throughout majority of complex tones, especially contiguous, suggests the presence of an additional "melodic" part. It can carry one of the following functions, known from typologies of multi-part musical textures of "frequency-based" music:

   a) An alternative polyphonic melody is characterized by melodic (numerous degrees and patterns of melodic contour), dynamic (intensity) and rhythmic (the rate of change) prominence, similar to the primary melody. For authentic Siberian and Far Eastern traditions, it is common to have the second melody contrast the primary one in some way (not as prominent as the contrast between the principal melody and the pedal bass). The most common form of contrast is in melodic contour (often inverse of the principal melody). Other forms of contrast might include number of degrees (smaller for lower melody and larger for higher), dynamic subordination (second melody softer than that of the primary melody), tonal typology (one melody is anhemitonic, while another – hemitonic or chromatic), and, sometimes, contour clarity ("thicker" contour, presence of gaps, aberrations in tuning from the harmonic series are usually more pronounced in the second melody).

   b) Potentially, there could be more than 2 melodic parts. Then the third melody should contrast the other two. The easiest cases, audible by ear, are those where two secondary melodies "sandwich" the principal melody. The third melody is usually weaker in intensity, sparser in melodic changes and less patterned than the second melody. Its melodic contour is usually closer to a flat linear shape – as compared to the pair of inversely zigzagging principal and secondary melodies.

   c) Much harder to detect is another scenario, where the 3rd melody presents a flattened version of one of the other two melodies – becoming its "satellite" in a sense of stressing and enriching a more important melody with the help of its "orbit." In such case changes in the direction of the 3rd melody are often out of phase with the other two. To detect such arrangement – and in general, to identify multiple melodies - it is handy to copy a portion of the spectrum that contains the suspected alternative melody and paste it into a separate file, then to normalize that file and audition it in comparison with the similarly isolated other textural components. If the alternative melodic line matches a principal melody by contour and in phase, then it should not be regarded as an autonomous melody – it merely

constitutes a "dub" of the principal melody (created by the sustaining a formant over the succession of JH tones).

4) The bass pedal part can also receive its own textural "satellite" – yet another pedal-like component, called to enrich or diversify the bass. Concerns about the monotony of the bass are likely to arise in JH traditions that have passed a long historic way of tonal development and reached the stage of individualized artistic mastery, when aesthetic satisfaction becomes an important part of JH musicking. Then the presence of a "bare" drone in the texture might be viewed as a display of artistic "uninventiveness." In such a case, it is the tenor part that is likely to take over the task of compensating for the bass' monotony, which can be achieved by a few options:
   a) Tenor pedal can contrast the bass by its harmonic richness - by encompassing a relatively homogenous "chord" of harmonics (e.g., f3/f4/f5/f6) as opposed to the stressed "single" harmonic of the bass part (e.g., f2). This is the simplest and the most common method of "enhancing" the bass.
   b) Occasionally, the repetitions of a tenor "chord" can mark a "melodic voicing fragment" – they can bring out a couple of different harmonics (e.g., f5-f6) inside of a "chord" in the manner of dynamically stressing a particular "voice" inside chords. The "voiced pattern" is usually comprised of the alternating adjacent harmonics.
   c) Sometimes, the tenor consists of the repetition of two "chords" as though in "inversion" (in the manner of inverting chords in Western music): e.g., f5/f6/f7 – f4/f5/f6. Here the idea of "pedal" is upgraded to the idea of "ostinato" – a continuously repeated pattern of rhythm and pitch – employed to embellish the accompaniment to the melody.
   d) The tenor part might contrast bass not in relation to harmonic "thickness," but melodic complexity. Then the monotony of the bass is offset by the presence of a purely melodic figuration, without any "chords" - deprived of harmonic thickness. An ongoing alternation of 2 pointillistic pitches, one of which falls always on strong, and another – on weak metric time is quite popular in Japan and Russian Far East. Such tenor makes an impression of a melodic figuration. The most common pattern is f3-f4, but one might encounter the usage of a "non-chordal" auxiliary tone – a pitch that is foreign to the harmonic series of the FF – a "step" above f3 (constituting the interval of a 6th or minor 7th in relation to FF – resembling the standard "boogie-woogie" drone).
   e) The ostinato figure in tenor can be further diversified into a pattern of 3-4 pointillistic pitches, repeated in the manner of the "Alberti's figure of the accompaniment" in classical music – just not as pedantic in repetition.

5) The last functional component in JH texture is comprised of the arrangement of the layer immediately above the highest melody. This is the least defined constituent compared to other parts. Often it is hard even to conclude whether there actually is a discrete extra part topping the highest melody. Even isolating the band under question, amplifying it and auditioning it in comparison with other parts often does not provide a clear answer. Two scenarios are most common:
   a) The highest melody can be offset by the "chords," or rather, chromatic (or even michrochromatic) "clusters," monotonously repeated in a manner similar to the "chordal" tenor. The extent of this monotony can vary from nearly perfect repetition of the same "cluster" to haphazard alternations of 3-4 "inverted" cluster-chords. At any rate, the monotonous or ostinato character of such chordal "finish" of the upper edge of a texture forms a clear contrast to the diversity and flexibility of the melody underneath it – similar to the contrast between the monotonous bass and the melody above it. In such case the melody (or the bundle of melodies) can be "sandwiched" between two pedals at the very bottom and at the very top of the texture. Such arrangement is likely to be intentional by the performer.
   b) Another common situation is when the material above the highest melody "dubs" either it or the melody under the highest melody – reproducing its melodic contour perfectly in phase, while slightly flattening it. This seems to be less likely a product of performer's deliberate effort in arranging the JH

texture and might present a mere byproduct of extra stress placed on the underlying melody. In our analysis we regard such cases as not constituting the textural part.

Processing a texture in the order outlined above allows to identify its principal components – sufficient to support cross-cultural comparison of different JH traditions.

To make the comparison of parts across different textures more thorough – and achieve higher distinction between different textural typologies – we extracted every single part from each studied texture into a separate file (without any modification of its dynamic properties) and ran frequency analysis on it. Selecting the entire file displayed all the "active" harmonics in it, reflecting their average tuning and amplitude. The latter enabled us to estimate the comparative loudness of one track in relation to another track within the same texture. Taking a note of which parts receive dynamic dominance in a texture allowed for more detailed categorization of textures. It turned out that certain types of textures are characterized by the dynamic prevalence of a specific part or by the dynamic equality of certain parts. This issue is perhaps the most important for classification of textures. Since greater loudness amounts to greater perceptual salience, the part that possesses the highest amplitude is very likely to be the "leading" part in the texture – thereby constituting the "backbone" of the arrangement of that texture. The quietest part, on the other hand, is likely to be the byproduct of the arrangement of a more salient part or perhaps even to be left to mere chance.

We also took a note of the frequency ranges for each part. This information makes it possible to compare textures produced within the same musical tradition on JHs made of different materials. It also allows to compare a specific part in one texture to the same part in another texture of the same type (e.g., alto to alto in a 3-part texture) in order to estimate its melodic dominance (bearing in mind that, the principal melody is characterized by wider range).

Closely related to the part's range is the number of degrees, engaged in building a melody. Listening to the isolated part reveals whether or not it bears a "melodic" or "chordal" function. In the first case, it appears as a discernible progression of "different pitches." In the second case, it sounds like repetition of more-or-less the same "chord." If the part is found to contain "melodic" voicing, it is necessary to identify the number of melodic "degrees" and their tonal relationship. The easiest way to identify the "degrees" is to keep pressing down the "forward" key on the computer keyboard while looking at the spectral contour of the frequency analyzer computer program – as the cursor keeps scrolling along the time line, certain partials peak out and then drop down. It is necessary to keep track of the frequency values for each of the peaks. The peaks that hit the same (or nearly same) frequency value at different points in time (especially upon the return to the same value after some other "pitch") indicate the "degree" of the timbral mode. The peaks that come close in frequency but do not coincide (about half-distance of the interval between two adjacent degrees) are likely to constitute some sort of "alterations" of the same degree.[3]

For each part we listed its most dynamically pronounced "pitches," defining them in terms of Western "notes" ("A," "B" etc.) most proximal to the frequency value discovered by the software frequency analysis tool. This "notation-like" denomination was chosen purely for the convenience of cross-relating "pitches" while observing their octave equivalence. This convenience outweighed our concerns for the bias introduced by the reference to Western music system. Had we avoided this bias by nominating degrees by their frequency values, it would have made it difficult to see which of the degrees remained "in tune" with their octave equivalents in other parts of the same texture – which would have prevented comparing different JHs in relation to the accuracy of their adherence to the harmonic series. Also, "note-like" representation facilitates evaluation of intervallic typologies – e.g., which mode is anhemitonic and which is hemitonic.

Once identified, the parts were named, using the choral terminology: bass, tenor, alto, soprano, and descant (in case of a voice above soprano). The names for the textural types were also borrowed from the Western music theory of polyphonic choral composition. The texture that features 2 melodies is called "duplum," 3 melodies – "triplum," and 4 melodies – "quadruplum."

[3] The ultimate answer to the question of how well the model of frequency-based "mode" suits the "timbral mode" and what constitutes a "timbral degree" will have to come from experimental research. But for now, it seems plausible to adopt the above-mentioned procedure for inferring degrees and defining a "mode" for each of the textural parts.

The instruments made of different materials differ by registral distribution of the “melodic motion”: some place it in tenor, others in alto or soprano. They also differ by the number of “melodic” parts (from 1 to 3) and by the general number of parts engaged in texture (from 3 to 5). Yet, additional important aspect of the difference comes from the functionality of voices: e.g., tenor contains either a melodic voice, an ostinato figuration or repetitions of more or less the same “chord.”

Samples of music for textural analysis of JHs made from organic materials are taken from the authentic traditions of Ural, Siberia, Middle Asia, Altai, Mongolia and Far East. The recordings of metallic heteroglot instruments include modern day styles of JH art music. Below, we provide descriptions of the most representative textures for each of the materials in the order of their chronological succession, as hypothesized in the main paper.

4. Grass idioglot JH: 5-part polyphony

Grass Jew’s harp is characterized by strong inharmonicity of its tones. It is impossible to infer a general “tuning” of an instrument – in sharp contrast to conventional Jew’s harps. Another of its characteristic traits is the high mobility of all its parts, constantly running in a non-scalar manner, mainly by 3^{rds}. This melodically “scattered” texture makes a rather accidental, non-premediated impression, probably resulting from “toying” with grass. Possibly, the loudest melody in tenor is the sole object of textural arrangement here – leaving the melodic changes in other parts up to chance. Absence of any differentiation between bass, alto and soprano testifies in favor of this conclusion. Only the highest part – descant – stands out with its softness, absence of gaps and the least number of degrees. The best way of naming this peculiar texture seems to be “**scattered quintuplum**.” Alto and soprano are dynamically second after tenor, separated from it by the significant difference of about 10 dB. However, they are poorly differentiated between each other – only by 1 dB. Perhaps, it would be justified to give a little edge to soprano, since it masks the lower alto. That is why we qualify soprano as the “first secondary” melody, whereas alto as the “second secondary.” Descant and bass form the third dynamic tier in this texture and are separated from the second tier of alto and soprano by 4 dB (for bass). For this pair, bass clearly is more salient due to 6 dB advantage and its position at the very bottom of the texture, which makes its degrees stand farther apart from each other compared to hemitonic soprano degrees. Wider intervals should make the melodic motion easier to track.

The sample is taken from a soundtrack to a documentary film where it illustrates how Nivkh *koka chnyr* is prepared and played by Zoya Angiun (Fig.1). http://chirb.it/M6kNww

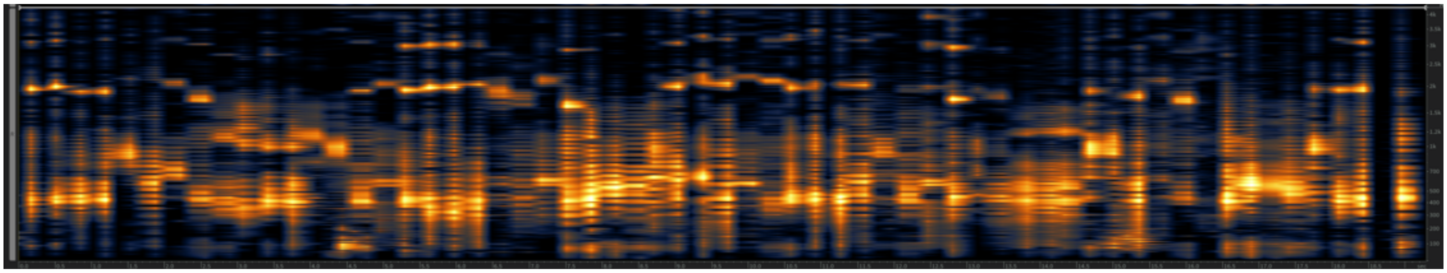

Fig.1: 5-part *“scattered” quintuplum* texture of the Nivkh *konga-chnyr*.

1. Bass (50-175 Hz) – tertiary melody, first (-47.7 dBu peak) melody C2-Eb2-F2-G2-Ab2-C3-D3-F3 (hemitonic hexatonic) http://chirb.it/0c4A60;
2. Tenor (205-686 Hz) – primary melody (-34.4 dBu peak) E4-F4-Ab4-Bb4(A)-Db5-D5-E5 (gapped hemitonic hexatonic) http://chirb.it/3sK4DL;
3. Alto (701-1300 Hz) – secondary melody, second (-45 dBu) melody Gb5(G)-Bb5(A)-B5-C6(C#)-D6 (gapped hemitonic pentatonic) http://chirb.it/mwAMgm;
4. Soprano (1330-2467 Hz) – secondary melody, first (-43.7 dBu) F6(E)-Gb6-A6-Bb6-B6-C7-Db7 (gapped chromatic heptatonic) http://chirb.it/wKrcPB;
5. Descant (2526-3757 Hz) – tertiary melody, second (-54 dBu) F7-F#7-G7-Ab7-A7 (chromatic pentatonic) http://chirb.it/2O2Kma.

Such texture constitutes the only type that is utilized by grass Jew's harps (judging by 3 different recordings that we have). This texture significantly differs from those generated by JHs made from other materials by its freely moving bass, maximal polyphony (all parts are melodic), minimal functional differentiation of parts and multitude of gaps in melodic motion of all parts except the highest one.

5. Bamboo idioglot JH: 4-part polyphony

Bamboo instruments feature 4-component textures with 2 melodies. They present greater variety of textures than grass JHs. The most common are 3 types, distinguished by the content of tenor and soprano. Bamboo tenor is lower and narrower than grass tenor, containing either ostinato figuration, alternative melody or a pedal chord. Alto features the principal melodic part, whereas soprano – the secondary, softer, melody, pedal chord or progressions of quite similar "chords" that emphasize a constituent tone that occasionally alternates a step higher or lower. If alto and tenor "melodies" are mostly "diatonic", soprano "melodies" are always "chromatic," and their "chords" constitute "chromatic clusters." All 3 textures share in common the leading alto part that contrasts soprano and tenor by its melodic shape and/or function (ostinato or pedal for tenor and pedal for soprano).

Type-1 presents a 4-part "**melodic ostinato duplum**" type of texture (Fig.2), typical for Far East. The term "duplum" comes from the Western medieval polyphonic theory and refers to the counterpoint of 2 melodic lines. The term "ostinato" refers to the characteristic melodic pattern present in tenor – it contains the ongoing repetition of a pair of pitches, one of which that receives metric stress is f3. Another pitch forms an auxiliary tone above f3, with a heptatonic (major or minor 2nd) or pentatonic (minor 3rd) step. This second pitch always comes at weak metric time in a dotted or, often, overdotted rhythm (punctured rhythm). Presence of this ostinato motion at the interval of 5th in relation to the FF is well audible in comparison with other JH textures. Both melodies, in alto and soprano, are about equal in intensity. E in tenor is a "non-chordal" tone in relation to the harmonic series of G. The other degrees are perfectly tuned to the natural series.

The sample was recorded from an anonymous Ainu player in Hokkaido in 1965 (Fig.2). http://chirb.it/ktqFmJ

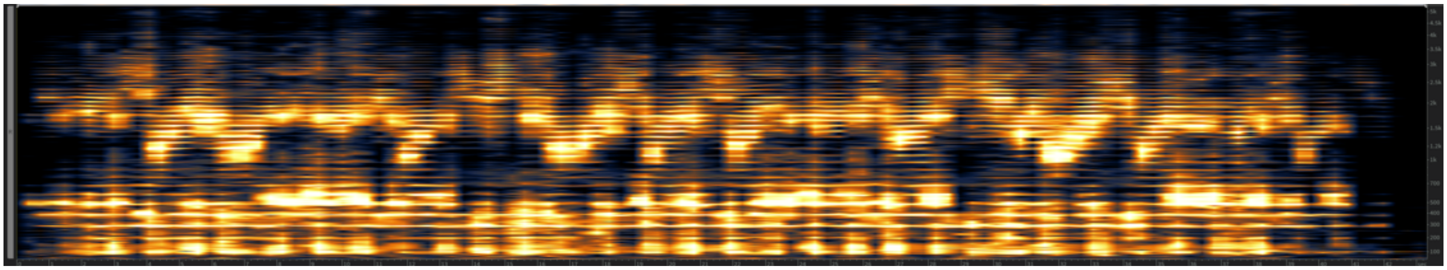

Fig.2: 4-part *melodic ostinato duplum* texture of the Ainu *mukkuri*.

1. Bass (10-210 Hz) – pedal tone ff=G2 (with resonant G1), f1 (G3) is the strongest (-37.3 dBu) (monotonic) http://chirb.it/IHsCtI;
2. Tenor (277-331 Hz) – *ostinato melodic figuration* (-36 dBu peak) based on the auxiliary tone D4-E4 (ditonic 2nd) http://chirb.it/3CKMmh;
3. Alto (352-800 Hz) – primary melody (loudest, -31 dBu) G4-B4-D5-E5-G5 (anhemitonic pentatonic) http://chirb.it/espmII;
4. Soprano (772-2606 Hz) – secondary melody (softer, *-34.7 dBu*) *C6-D6-E6-F#6-G6-A6-(A#6)-B6-C7-D7-E7* (diatonic heptatonic) http://chirb.it/gxzJNp.

Type-2 presents an alternative, 4-part "**chordal pedal duplum**," type, typical for Altai and Mongolia (Fig.3). Here, tenor contains a pedal chord that thickens and colors the bass, providing a harmonic cushion for the melodic motion (this difference from the Type-1 texture is marked by the italic font in the listing of parts). Subsequently, the alto melody is pushed higher in register. Yet another point of difference is a much wider alto's diapason in comparison to Type-1. This further emphasizes alto's salience – in addition to its dynamic dominance. By the same token, soprano here is downgraded in its melodic importance due to its much greater softness and scarcity

of degrees as opposed to Type-1 soprano. All lower tones in tenor and alto melodies are perfectly tuned. http://chirb.it/xzI0AD

1. Bass (42-112 Hz) – pedal tone ff=Eb2, where f1 (Eb3) is the strongest (-56.3 dBu) (monotonic) http://chirb.it/28stMJ;
2. Tenor (200-630 Hz) – *pedal chord* Bb3/G4/Db5 (-47.1 dBu peak) (triadic) http://chirb.it/xkk1cn;
3. Alto (710-2517 Hz) – primary melody (louder, -35 dBu) F5-G5-Bb5-C6-D6-Eb6-F6-G6-A6-Bb6-C7-Db7 (diatonic hemitonic) http://chirb.it/NtOA5K;
4. Soprano (2833-3698 Hz) – secondary melody (soft, *-54 dBu*) *Gb7-G7-Ab7-A7* (chromatic tetratonic) http://chirb.it/mhAvz4.

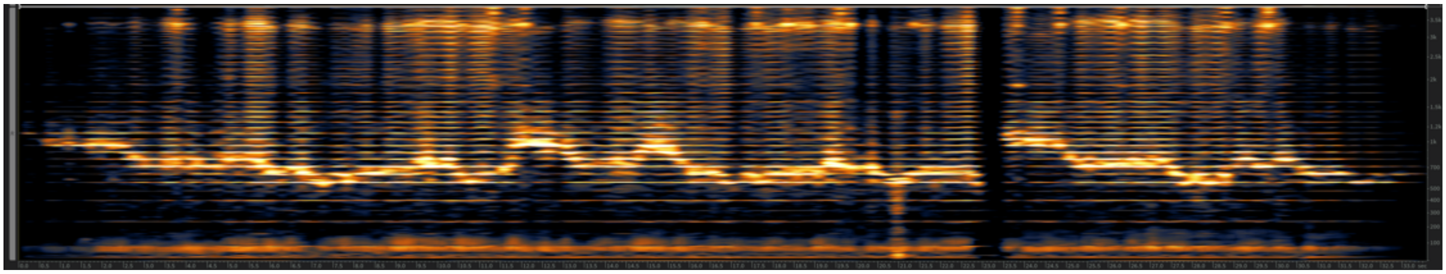

Fig.3: 4-part *chordal pedal duplum* texture of the Altaic *cheler khomus*. Nearly identical textures are used by the Mongolian *khulsan khuur*. Such textures are also found in the Ainu *mukkuri* music.

Type-3 presents yet another textural model (Fig.4). We can call it a 4-part "**framed duplum**" - since its two melodies are "sandwiched" between the two pedals at the very bottom and top of this texture (which might be noticeable upon auditioning). Of the two melodies, the alto retains its dominance – as in Type-1 and Type-2. But the secondary melody here moves to the part *below* the principal part. Soprano rather strictly sustains a pedal "chord," thereby contrasting the melodic parts below it. The reason for considering this upper "chord" for a "part" here, but not in Type-1, is that here it is not the last uppermost component that is *structured* – above it there is the "dub" of the alto part. Therefore, a sustained repetition of the same "chord" constitutes a trait of texture rather than mere absence of textural arrangement, as in Type-1. However, overall, this texture is very similar to Type-1 in its dynamic near-equality of both melodies. All lower tones in tenor and alto melodies match the FF harmonic series. http://chirb.it/vFrL8M

1. Bass (61-330 Hz) – *pedal* tone ff=E2 (82.8 Hz), where f1 (E3) is most pronounced (-33.1 dBu) (monotonic) http://chirb.it/638HN3;
2. Tenor (415-912 Hz) – *secondary melody* (softer, -20.4 dBu) G#4-D5-F#5-A#5 (gapped whole-tone) http://chirb.it/Fwnwqz;
3. Alto (994-1991 Hz) – primary melody (louder, -18.8 dBu) C6-C#6-D#6-F6-G6 (hemitonic pentatonic) http://chirb.it/378BkB;
4. Soprano (2073-3399) – *pedal chord* (-40.6 dBu) (cluster) http://chirb.it/KDvhdy.

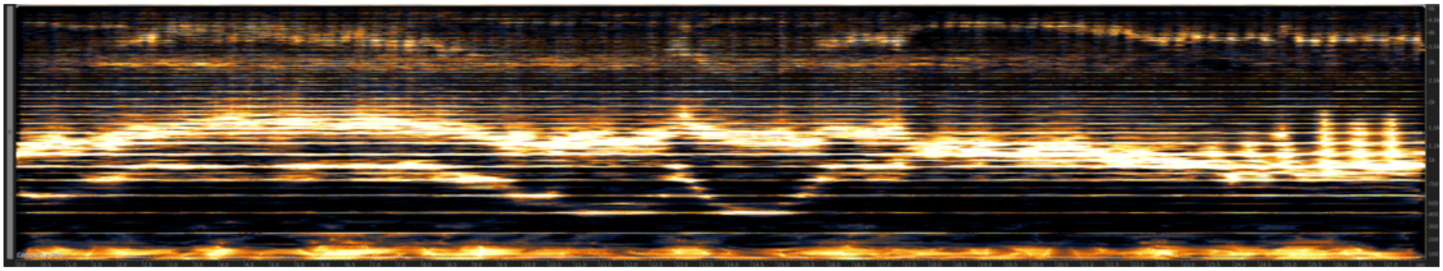

Fig.4: 4-part *framed duplum* texture of the Ainu *mukkuri*.

Out of all materials, bamboo stands out by perhaps the greatest flexibility and diversity of textures – so that it is quite common to encounter modulations from one textural arrangement to another within the same musical work, even in the performance of ordinary "village" players (as opposed to renowned masters).

The example below illustrates the textural change from Type-3 to Type-4 (Fig.5 – on the right side). The latter seems to be a variation of Type-1 – based on the same idea of ornamenting the tenor part in order to diversify the drone (engaging the same "punctured" rhythm). However, in Type-4 the ornamentation involves not melodically isolated "pitches," but the pattern of two "chords" in which the upper "voice" is dynamically brought out. In essence, such tenor constitutes the ostinato figure of 2 chords. We can distinguish this type by calling it "**chordal ostinato duplum**" to reflect its greater complexity and richness in comparison to Type-1. Here, both, tenor and alto, remain dynamically dominant, whereas soprano plays a secondary, supporting, role. Of all bamboo textures, this texture is the least stable in tuning – in its tenor component. http://chirb.it/yF2wJd (the provided audio clip illustrates only the texture of the second portion of Fig.5).

1. Bass (50-255 Hz) – pedal tone ff=E2, where f1 (E3) is most pronounced (-39.9 dBu) (monotonic) http://chirb.it/16hpm0;
2. Tenor (321-1141) - *ostinato figuration* of the auxiliary melodic motion E5-F#5, where both, E5 and F#5, are supported with a "*chordal" complex* E4/B4/E5 – G#4-D5-F#5; this figuration keeps shifting slightly higher in register (-14.2 dBu) (9th-chordal) http://chirb.it/6nAAeq;
3. Alto (1160-2320 Hz) – primary melody (loud, -27.1 dBu) D#6-F6-G6-A6-A#6-(B6)-C7 (diatonic heptatonic) http://chirb.it/PzGerN;
4. Soprano (2324-3483 Hz) – secondary melody (soft, -39.4 dBu) D7-E7-F7-G7 (diatonic tetratonic) http://chirb.it/wByras.

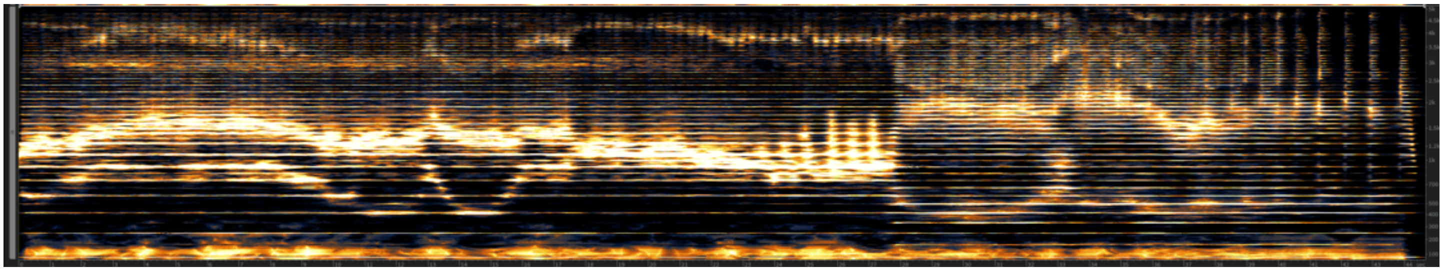

Fig.5: 4-part *chordal ostinato duplum* texture of the Ainu *mukkuri* (here, it succeeds the texture from the previous Fig.4 - 28 seconds after the beginning of the clip – this section of the spectrogram corresponds to the audio clip http://chirb.it/yF2wJd).

6. Wood idioglot JH: 4/5-voice polyphony

Wooden instruments usually employ more complicated polyphony, assigning a melodic function to tenor, which increases the number of melodic voices to 3. Hence, wooden textures differ from "duplum" bamboo textures by constituting a "**triplum**" polyphony. It is characterized by the opposition of changeable and diverse material of the melodic trio to the monotony and simplicity of the bass pedal. Here, the drone component of texture is drastically reduced to a single "pitch" in the lonesome bass part - comparing to the bamboo textures with their intricate ornamentation of the drone component. This makes wooden textures much more linear (in a form of either melodic line or a pedal "tone"). Subsequently, differentiation of melodies becomes the most important aspect in the arrangement of wooden textures. Cultivation of wooden JHs undoubtfully promotes the development of polyphonic musical thinking in order to support simultaneous control of 3 melodic lines. Even greater complexity offers a 5-part texture, where the pedal chordal part caps the soprano melody. Overall, wooden textures tend to exceed bamboo and grass in textural complexity. Tonally, they lean towards chromatic melodic motion – the principal alto "melodies" are usually chromatic, in contrast to bamboo.

Type-1 presents a 5-part "**framed triplum**" texture (Fig.6) of the Itelmen *varyga*. This texture is characterized by two pedals framing 3 melodies from below and above that melodic trio. The idea of the multi-layered mobility in the center of this texture versus the steadiness of its margins seems to govern its arrangement. Two lower melodies are about equal in intensity, while the 3rd, higher, is significantly weaker. However, the alto melody significantly exceeds the tenor melody in its number of degrees and contrasts it tonally (chromatic alto versus anhemitonic tenor). Tonally, soprano does not differ from alto (both are chromatic), but it engages fewer degrees.

In relation to tuning, wood seems to be slightly more finicky than bamboo. The tones A4 and F5 in tenor are sharper than the harmonic series of C2. http://chirb.it/gxhHrw

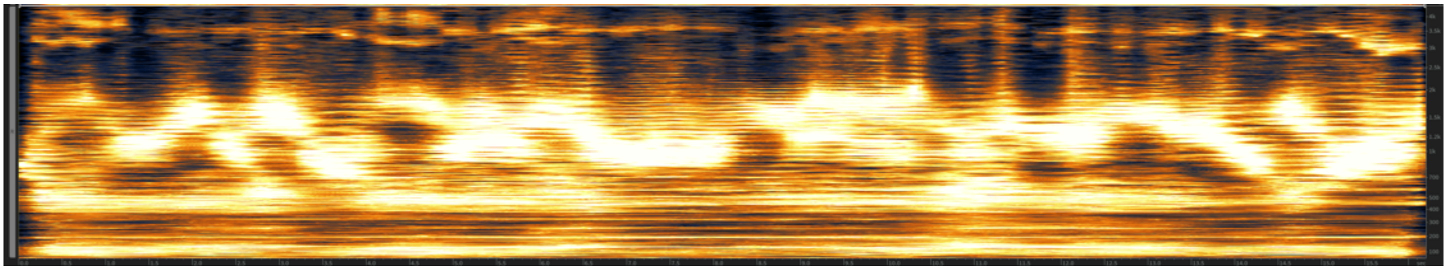

Fig.6: 5-part *framed triplum* texture of the Itelmen *varyga*.

1. Bass (63-210 Hz) – pedal tone ff=C2, where f2 (C2) is the strongest (-40.1 dBu) (monotonic) http://chirb.it/eeDfLC;
2. Tenor (221-703 Hz) – *secondary melody* (loud, -39 dBu) A4-C5-D5-F5 (anhemitonic tetratonic) http://chirb.it/veEM5m;
3. Alto (684-2310 Hz) – primary melody (loud, -38 dBu) F#5-G5-G#5-A5-A#5-B5-C6-C#6-D6-E6-F#6-G6-G#6-A6 (chromatic) http://chirb.it/KMa5xq;
4. Soprano (2470-3601 Hz) – tertiary melody (quieter, -59.3 dBu) D#7-E7-F7-F#7-G7-G#7-A7 (chromatic heptatonic) http://chirb.it/LmwOFy;
5. Descant (3634-5175 Hz) – *pedal chord* with occasional melodic auxiliary motion A#7-B7-C8 (quietest, -64.5 dBu) (cluster) http://chirb.it/r0rxDJ.

Type-2 presents a 4-part "**simple triplum**" texture of Kirghiz *jigatch ooz komus* (Fig.7). It is noticeably simpler than Type-1 while retaining the same principle of differentiation within the melodic trio in contrast to the thin pedal in bass. The principal difference from Type-1 is the replacement of the upper chordal pedal with the dubbing of a highest melodic part. Therefore, this type is more "melodic" than Type-1. Of the 3 melodic parts, the middle part – alto – dominates dynamically and by the number of its degrees. Tonally, 3 melodies form the same relations as in Type-1. The tones are perfectly tuned. http://chirb.it/hvLyp1

1. Bass (62-267 Hz) – pedal tone, ff=B1, where f2 and f4 (octave) are the most prominent (-48.1 dBu) (monotonic) http://chirb.it/fymJc3;
2. Tenor (284-1054 Hz) – secondary melody (quieter, -36 dBu) F#4-B4-C#5-D#5-E#5-B5 (hemitonic pentatonic) http://chirb.it/t47sJP;
3. Alto (1115-2168 Hz) – primary melody (loudest, -27.4 dBu) D6-F6-F#6-G6-G#6-A6-A#6-B6-C7-(C#7) (chromatic) http://chirb.it/2yqwK0;
4. Soprano (2232-3407 Hz) – *tertiary melody* (quietest, -43.7 dBu) D7-D#7-F#7-G7-G#7 (gapped chromatic pentatonic) http://chirb.it/547tKA.

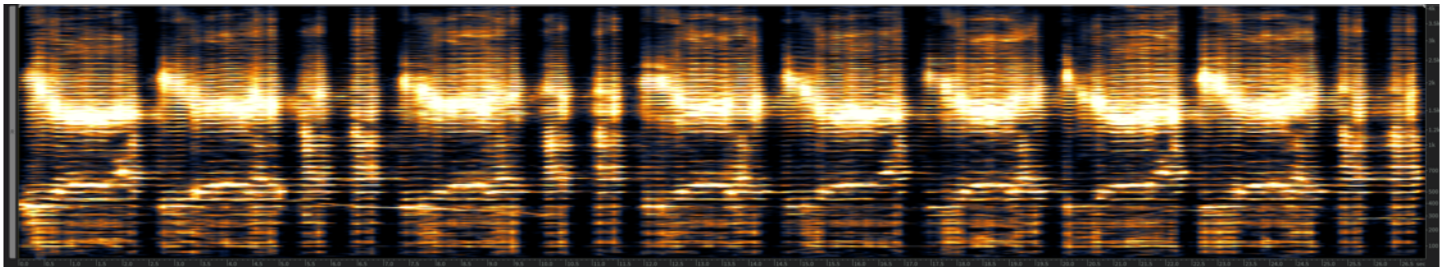

Fig.7: 4-part *simple triplum* texture of the Kirghiz *jigatch ooz komus*.

7. Bone idioglot JH: 4-part polyphony

Bone instruments seems to adopt the wooden triplum types, but with more "chromatic" melodic motion that might even become inharmonic. Melodies of bone instruments resemble those of wooden instruments also in regard to

their relative "thickness" – wider in frequency range than bamboo and metallic instruments. There seem to be very few differences between wooden and bone textures. Bone appears to provide less definition in voicing of parts and the overall tendency to use lower range for its upper parts, especially noticeable in soprano. This must be responsible for poorer differentiation between melodies. If wooden tenor is always weaker than wooden alto, for bone instruments either of them can contain the principal melody. Yet another difference is that the bone bass tends to exhibit greater richness than the wooden bass – sometimes employing a "chord" rather than a single "pitch" as pedal.

Type-1 presents the 4-part **simple triplum** type (Fig.8), very similar to the wood Type-2 – except that here the tenor melody (rather than alto) dynamically prevails. However, all 3 lower parts remain very close in their amplitude. Only soprano is markedly softer. The tuning is not perfect here: the tenor melody contains 3 lowered harmonics - G4, Db and Eb (instead of Ab, D and E). http://chirb.it/e6reg6

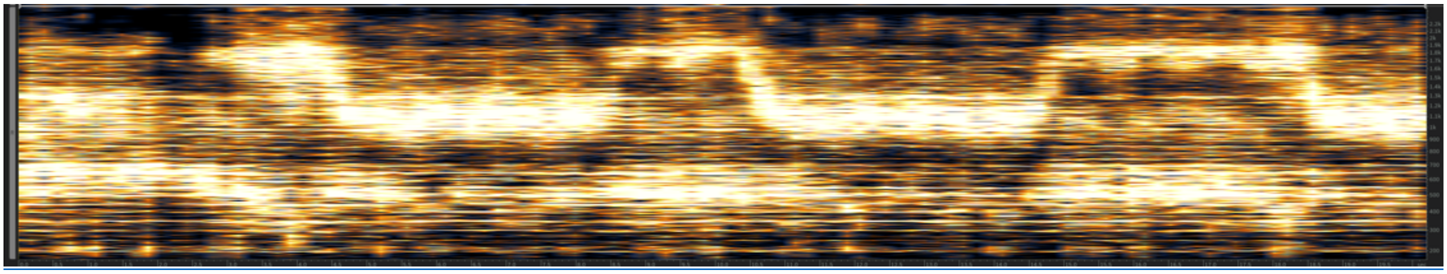

Fig.8: 4-part *simple triplum* texture of the Mansi *tumran*.

1. Bass (50-228 Hz) – the pedal dyad ff=Bb1, f1 and f3 (5th over octave) are the most prominent (-35.6 dBu) (ditonic 5th) http://chirb.it/Ph47qs;
2. Tenor (263-964 Hz) – *primary melody* (louder, -32 dBu) G4-Bb4-C5-Db5-Eb5 (hemitonic pentatonic) http://chirb.it/bgq7P1;
3. Alto (990-1938 Hz) – secondary melody (quieter, -36.3 dBu) E6-F6-Gb6-G6-Ab6-A6-B6 (chromatic heptatonic) http://chirb.it/nFNA9D;
4. Soprano (c Hz) – tertiary melody (quietest, -56.8 dBu) C7-Db7-D7-Eb7-E7-F7-Gb7 (chromatic heptatonic) http://chirb.it/7tG0BN.

Type-2 presents a variation of the same simple triplum style (Fig.9), performed on the same instrument, tumran, but by a Khanty player (rather than Mansi as in Type-1). This texture gives dominance to alto rather than tenor – albeit by a tiny (hardly distinguishable) margin. The 3rd melody is dynamically closer to the other two, unlike in the melodic trio of Type-1. Yet another difference is that the bass part here is harmonically richer, constituting a "chord" (G/C/E). Tonally, the tenor melody exhibits significant deviations from the harmonic series of C of its fundamental C. http://chirb.it/NzF5ED

1. Bass (48-364 Hz) - the pedal tone ff=C2, while f3 and f5 (triad) are the most prominent (-39.2 dBu) (triadic) http://chirb.it/JK8wsK;
2. Tenor (366-728 Hz) – secondary melody (quieter, -35.2 dBu) G4-A4-A#4-B4-C#5-(D#5)-E5-F5(F#5) (hemitonic) http://chirb.it/nbatJI;
3. Alto (730-1513 Hz) – *primary melody* (louder, -34.9 dBu) melodic voice G5-G#5-C#6-D6-D#6-E6-F6-F#6 (chromatic) http://chirb.it/gvF691;
4. Soprano (1528-2378 Hz) – tertiary melody (quietest, -44.4 dBu) G6-A6-A#6-B6-C#7 (chromatic) http://chirb.it/kJCbss.

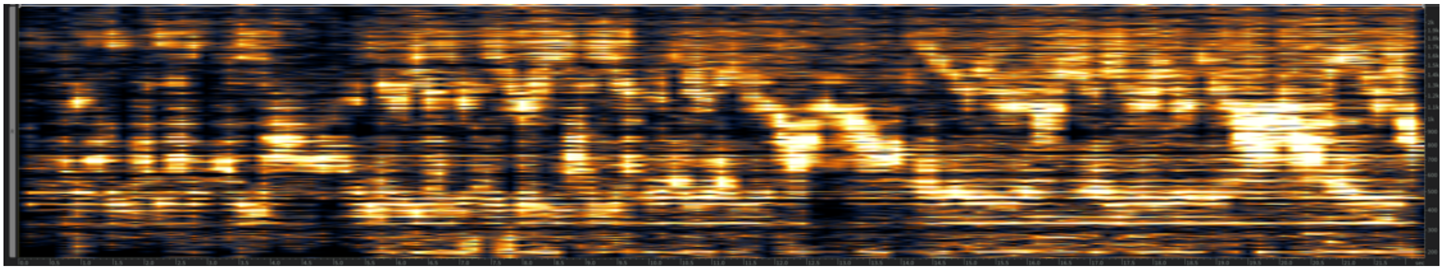

Fig.9: **4-part *simple triplum* texture of the Khanty *tumran*.**

8. The comparison of wood, bamboo and bone

We have seen that bamboo and wood textures are quite different from each other, whereas wood and bone textures are hard to distinguish from each other. Therefore, we asked Erkin Alekseyev to provide demonstrations of the same set of articulations on JHs made from each of these materials, from the Museum's collection.

Yakut bone khomus (Fig.10) possesses a hollow sound, with clucking-like attack. Its component pitches are more indefinite in pitch and not harsh, somewhat gently "rolled" – in comparison to wood and bamboo. http://chirb.it/s3y8PI

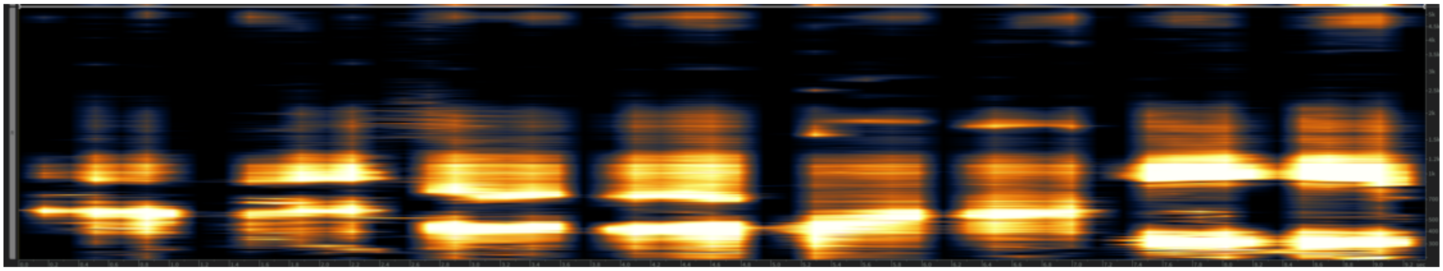

Fig.10: **Yakut bone *khomus*. Comparative demonstration by Erkin Alekseyev.**

Wooden instrument sounds denser, drier and harsher than bone (Fig.11). Its attack generates noticeably higher partials that do not blend with the fundamental, producing a shorter "clang" tone. Continuous articulations have more of a clattering sound. In general, neither bone nor wood in these demos sound that harmonious, and their fundamental tone is hardly identifiable. http://chirb.it/7ryLCd

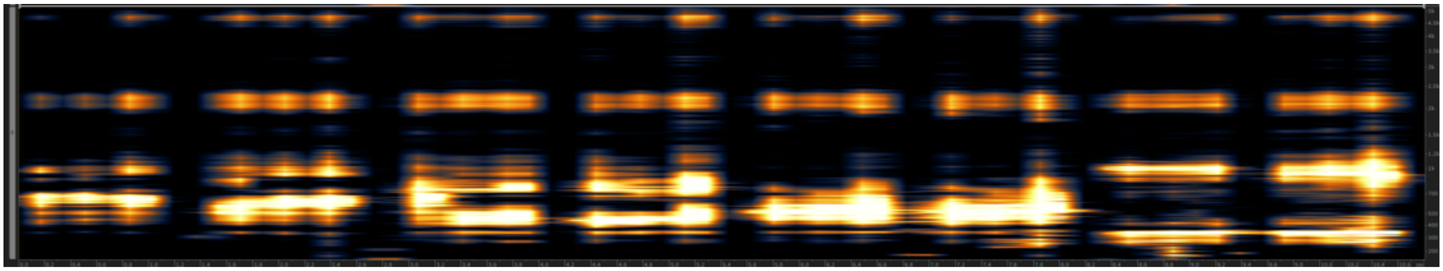

Fig.11: **Evenk wooden *khomus* from Krasnoyarsk Krai. Comparative demonstration by Erkin Alekseyev.**

In contrast, bamboo produces partials mostly belonging to the FF=C2 harmonic series (Fig.12). Its sound is about as hollow as that of bone, but is easily recognized by its characteristic rattling quality. http://chirb.it/95ecD9

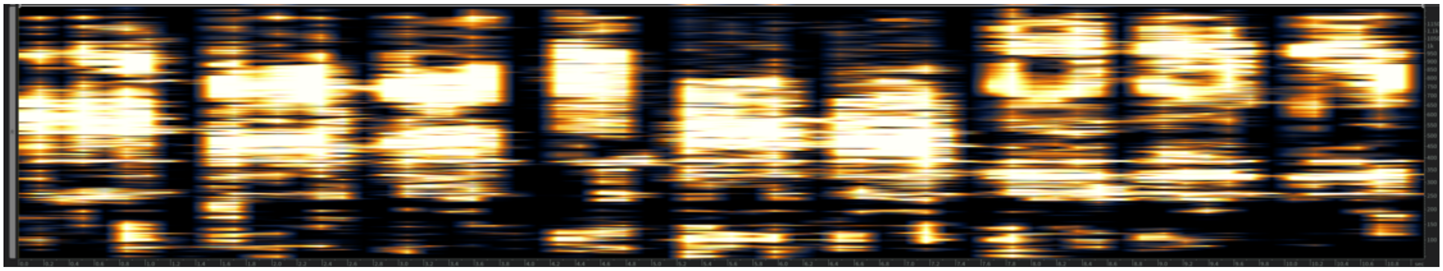

Fig.12: **Tuvan *kuluzun komus*. Comparative demonstration by Erkin Alekseyev.**

Another trait that allows to distinguish all 3 materials is the registral position of the loudest portion of the spectrum for each of these materials. The sound of bamboo is the lowest of the three: its loudest partials (≥ -70 dBu) throughout the entire 10-second clip occupy a range of 50-1201 Hz. The wooden instrument is clearly brighter: its loudest partials (the same ≥ -70 dBu) spread over a much wider range: 332-6190 Hz. And the bone instrument occupies an intermediate position, with the range of 134-4690 Hz.[4]

9. Brass idioglot JH: 4/5-part polyphony

Our review of the metallic JH textures should start with the idioglot construction, since (as we have already explained in the main paper) this construction reproduces the regional constructions traditionally made from organic materials. Along with its construction are reproduced those musical textures that characterize local traditions of bamboo JHs. Metallic idioglot JHs are common around the Far East region and present a much later development comparing to the metallic heteroglot instruments of Eastern Europe and Middle Asia – in the order of 1000 years and even more. Thus, ethnicities of Primorye and Sakhalin adopted idioglot metallic JHs from Russian settlers in the 18th century (Mamcheva 2005).

Of all metals, the most common for idioglot construction is brass. Brass frame-shaped instruments are much easier to play than any other metals, according to the folk players from Sakhalin – even easier than wooden instruments of the same construction type (Mamcheva 2012, 50). As easy can be the manufacturing of such an instrument. Malleability of brass enables virtually anyone to make a Jew's harp from a flattened rifle cartridge case – without resorting to a blacksmith's service. It is not surprising that brass frame-shaped instruments generate basically the same textures as wooden instruments.

Type-1 presents the most common case of **4-part simple triplum** texture (Fig.13), typical for wood (its Type-2). The only noteworthy difference is the dynamic balance between parts. In general, metallic instruments noticeably weaken the bass pedal (20 dB difference between the melody and the bass, as in the example below, is not by any means uncommon) compared to organic instruments (where such difference usually constitutes about 5 dB). As a result, monotony of the JH texture becomes significantly reduced in music that is produced on metallic JHs. The parts under the melody acquire a softer sound, more appropriate for the accompaniment to a principal melody – in line with the idea of homophonic arrangement.

Brass idioglot frame-shaped constructions differ from their wooden counterpart in a few more dynamic respects. For wood, the alto melody usually exceeds other melodic parts in intensity (greatly: >11 dB) and the number of degrees (about twice more than the second closest). For the brass version of this texture all 3 melodic parts are much closer in their intensity – especially between tenor and alto, that are also close in the number of degrees in their melodies. A slight dynamic lead in melodies of the brass frame-shaped construction usually goes to tenor (unlike the alto lead of the wooden JHs). Emphasis on the lower position melodic part coincides with the overall "sagging" of all registers: every single part in the texture becomes lower compared to the same part in wood. Subsequently, idioglot brass instrument sounds closer to a bamboo instrument than to a wooden one. Another common characteristic trait with the bamboo JH is that idioglot brass instrument has a pronounced rattling – even harsher than that of bamboo. http://chirb.it/vH1HNh

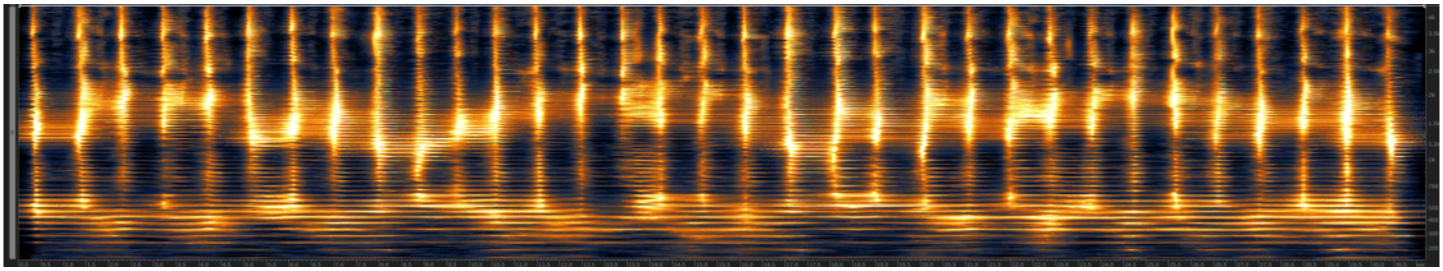

Fig.13: 4-part *simple triplum* texture of the Nivkh brass *kanga*.

[4] All values are taken in the application program RX by iZotop - at FFT size 262144, Hann window, without time overlap, averaged channels and decay of 80 dB/s.

1. Bass (41-189 Hz) – pedal tone ff=Gb1, where f4 (Gb3) is the strongest (-70.8 dBu) (monotonic) http://chirb.it/fHN5Mh;
2. Tenor (218-753 Hz) – *primary melody* (loudest, -49 dBu) Fb4-Gb4-Ab4-Bb4-C5-Db5-Eb5 (diatonic heptatonic) http://chirb.it/A2dygz;
3. Alto (803-2313 Hz) – secondary melody (quieter, -50.9 dBu) Db6-D6-Eb6-E6-F6-Gb6-G6-A6-Bb6-B6-C7 (chromatic) http://chirb.it/OLOvxd;
4. Soprano (2361-3161 Hz) – tertiary melody (quietest, -62.1 dBu) D7-Eb7-E7-F7-Gb7 (chromatic pentatonic) http://chirb.it/nsBHKq.

Type-2 mixes two bamboo textures – **chordal duplum** and **framed duplum** - into a new **5-part chord-framed duplum** (Fig.14). With the bamboo chordal duplum, it shares the "chord-based" tenor that harmonically enriches the bass pedal. With the bamboo framed duplum, there are a number of common traits. First and foremost, both melodic parts are "sandwiched" between the lower and the upper pedals. Second, both melodies are almost equal in intensity. Third, they also come close in their number of degrees. Of the 5 parts, 3 contain pedals, offsetting 2 melodic voices (soprano and alto). One notable difference from bamboo is the bandwidth compression and registral lowering of all melodic parts, so that soprano range ends more than 1 kHz lower. Another difference is tonal: brass alto is chromatic, whereas bamboo alto – diatonic. Overall, it seems that this type originates from the increased control over the harmonic arrangement of parts in comparison to the JHs made of organic materials. The drone is split into monotonic bass and chordal (harmonic) tenor, and the upper finish of this texture features a chordal layer over the soprano melody. http://chirb.it/AMvA4G

1. Bass (40-169 Hz) – *pedal* tone ff=E1, where f2 (E2) is the strongest (-49.7 dBu) (monotonic) http://chirb.it/xPf654;
2. Tenor (173-307 Hz) – *pedal chord* G-Bb-Db (triad) (-47.7 dBu) (triadic) http://chirb.it/HEahvC;
3. Alto (318-735 Hz) – primary melody (louder, -37.6 dBu) F#4-G4-A4-B4-C5-C#5-D5 (chromatic heptatonic) http://chirb.it/GK7kmm;
4. Soprano (758-2194 Hz) – secondary melody (quieter, -40.1 dBu) A5-C#6-D6-D#6-E6-F6-F#6-G6-G#6 (chromatic) http://chirb.it/e8fn69;
5. Descant (2201-3673 Hz) – *pedal* chord (-60.8 dBu) (cluster) http://chirb.it/PkHOHO.

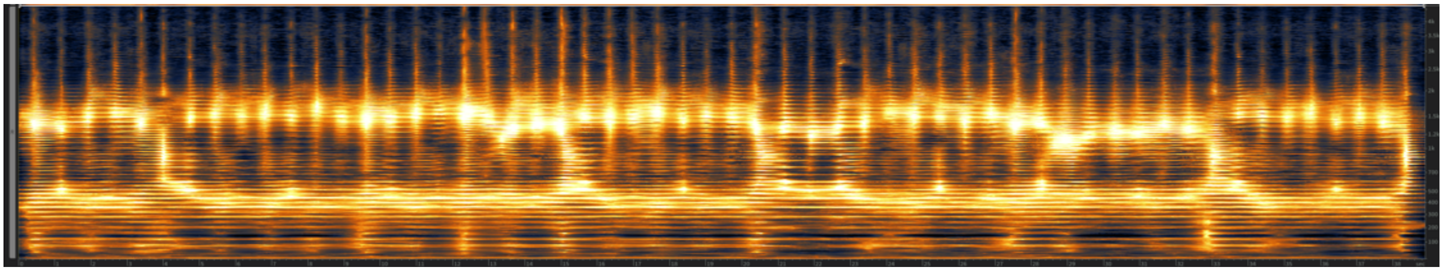

Fig.14: 5-part *chord-framed duplum* texture of the Nivkh brass *kanga*.

10. Copper heteroglot JH: 4-part polyphony

Textures produced on heteroglot metallic constructions substantially differ from the idioglot metallic construction – as we could clearly observe by comparing the spectrograms of brass heteroglot and brass idioglot JHs. Heteroglot textures typically feature homophonic arrangement versus idioglot textures that always (in our experience) engage polyphonic melodies – as we shall show below. But for now let us start our review of heteroglot metallic JHs from copper – chronologically the first metal whose technology was mastered across Eurasia. Subsequently, the textures could set the standard for heteroglot metallic JHs to come.

Heteroglot copper usually employs the "**framed duplum**" texture (Fig.15) – quite simpler in its design than 5-part chordal framed duplum (one part less) and 4-part simple triplum (one melody less) of the idioglot brass instruments.

The framed duplum type is very typical for bamboo. Perhaps, the only important difference between bamboo and copper is that bamboo provides superior isolation of voices and “pointillistic” shape of melodic motion of the prevailing “diatonic” style. Copper melodies are broader in bandwidth. Also copper noticeably amplifies frequency bands above the alto melody, which enriches higher registers, giving them “chordal” sound. Subsequently, copper instruments sound “harmonic” rather than “melodic” compared to bamboo instruments. Yet another difference belongs to the tonal domain: unlike bamboo, copper generates chromatic rather than diatonic melody in alto part – this is because of a higher registral position of alto (hence, engaging higher partials that are separated by tighter intervals). In general, bamboo tends to activate the ranges lower than other organic materials.

The most common heteroglot copper texture is a 4-part “**framed duplum**”, typical for many metallic heteroglot constructions (as well as for idioglot bamboo constructions). For copper, this texture is characterized by the contrast between a couple of dynamically more or less equal melodic voices “sandwiched” between the softer pair of pedal-based parts, also approximately equal in intensity. All lower tones in both, tenor and alto melodies, are perfectly tuned. http://chirb.it/FmD3Pk

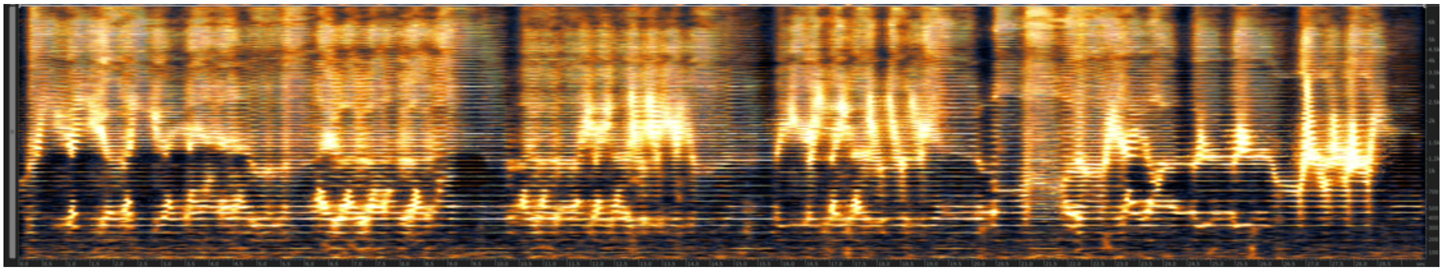

Fig.15: 4-part *framed duplum* texture of the modern Russian copper *vargan*.

1. Bass (64-264 Hz) – pedal tone ff=C2, where f4 (C4) is the most prominent (-57.3 dBu)[5] (monotonic) http://chirb.it/ENEEDg;
2. Tenor (326-783 Hz) – secondary melody (quieter, -43.2 dBu) E4-G4-Bb4-C5-D5-F5-G5 (anhemitonic) http://chirb.it/FLtOvA;
3. Alto (848-3524 Hz) – primary melody (louder, -40.1 dBu) G#5-B5-C6-C#6-D6-D#6-E6-F#6-G6-Ab7-Bb6-B6-C7-C#7-D7-D#7-E7-F7 (*chromatic*) http://chirb.it/wJrGp3;
4. Soprano (3590-6332 Hz) - pedal “chords” are repeated over with occasional fluctuation higher or lower (-50 dBu) (cluster) http://chirb.it/yfg871.

11. Bronze heteroglot JH: 4-part polyphony/3-part homophony

Like copper, bronze JHs often employ the same “framed duplum” texture. Many performers who play string and wind instruments consider bronze to provide a warmer and darker sound than the “shiny” copper. JH spectrograms illustrate this difference. The entire range above the alto melody is considerably more intense in copper JHs (Fig.15) than it is in bronze (Fig.17). This overall excitement, however, reduces the salience of the melodic material – spectrograms of bronze JHs reveal a much better differentiation between the harmonics engaged in the melodic line and the surrounding audio material in the spectrum. In bronze, as compared to copper, there are more vibration modes that become excited, which results in textures where the principal melodic line receives many more registral “dubbings” – each of which is quite well marked (a “dubbing” of a melody visually represents a single vibration mode). Subsequently, the spectrogram of the bronze JH resembles the spectrogram of a melody performed in unison by the orchestral “tutti.”

For this reason, harder metals promote dominance of a single “melody” in the texture - which causes considerable simplification of JH textures comparing to bamboo and bone, not to speak of wood. Subsequently, bronze, being harder than copper, enables a simpler **3-part texture** that is based on a **single melody**.

[5] G2 seems to be introduced by the electric humming in a room.

Type-1 exemplifies such texture (Fig.16). This can be called **3-part** "**framed homophonic**" because it features a single melodic line, accompanied by a bourdon pedal below and pedal chords above the melody. The full spectrogram of such texture usually displays a well-defined "ripples" of multiple "dubbings" of the melody - elevating across the higher registers (Fig.17 captures only the lowest of these "ripples" – because the spectrogram was trimmed at 9 kHz). Each of these 3 parts is well differentiated from the others. The bass part differs from the alto by its thinner composition (2-"pitch" drone versus thicker "cluster-chords" of the alto part) as well as by a greater intensity. Single melody receives a much wider available space – so, its ambitus often exceeds that of melodies in duplum and triplum textures. The tones F#5, D6 and F6 are slightly flatter than the harmonic series. http://chirb.it/chLLBh

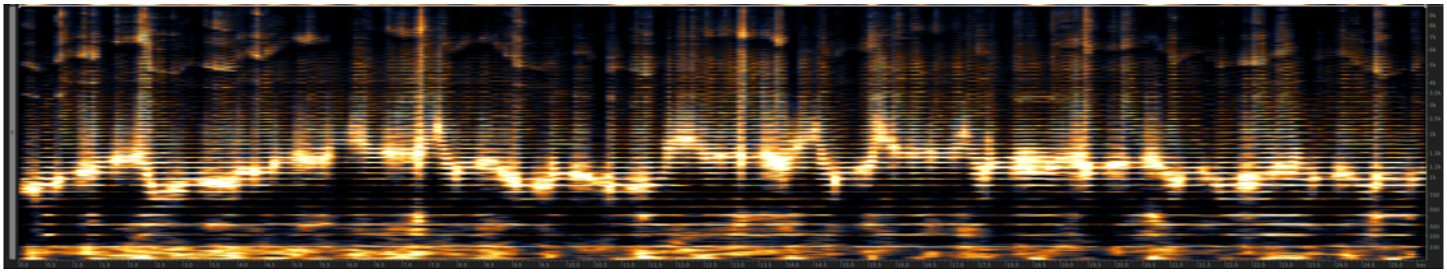

Fig.16: 3-part *framed homophonic* texture of the bronze Magyar *doromb*.

1. Bass (47-432 Hz) – *pedal* tone ff=A2, where f1 and f3 (5th) are the most prominent (-42.2 dBu) (ditonic 5th) http://chirb.it/r03G3q;
2. Tenor (540-2807 Hz) – melody (-32 dBu) F#5-A5-B5-C#6-D6-E6-F6-Gb6-Ab6-A6-Bb6-C7-Db7 (hemitonic) http://chirb.it/6Bzdyy;
3. Alto (2917-4213 Hz) – *pedal* "chords" (-52.6 dBu) are repeated over and over with little variation, marking a little "sub-voice" that consists of a few "pitches." (cluster) http://chirb.it/s3ByGq.

Type-2 provides an example of copper-like polyphonic **4-part** "**framed duplum**" texture (Fig.17), with exactly the same dynamic balance. However, its upper registers are not as saturated as those in the copper clip. The principal melody in bronze JHs is significantly richer than in copper: in regards to the number of degrees and their tonal relations. It seems that bronze is a less harmonious material than copper. The tones A4, C4 and D#5 are slightly flatter than the harmonic series. As a result, both, tenor and alto melodies are neither diatonic nor contain "false relations" between their degrees. http://chirb.it/4N8fMg

1. Bass (37-220 Hz) – *pedal* tone ff=F#2, where f1 and f2 (octave) are the most prominent (-48.4 dBu) (monotonic) http://chirb.it/4JOCsP;
2. Tenor (270-631 Hz) – secondary melody (quieter, -31.8 dBu) C#4-F#4-A4-C5-D#5 (diminished 7th-chord *arpeggio*) http://chirb.it/n2EeA4;
3. Alto (727-3520 Hz) – primary melody (loudest, -28.4 dBu) F#5-G#5-A5-B5-C#6-D6-E6-G6-A6-D#7-F7 (*gapped hemitonic*) http://chirb.it/ArhHOh;
4. Soprano (3256-5236 Hz) - *pedal* "chords" (-63.4 dBu) with a marked tone that infrequently fluctuates (cluster) http://chirb.it/s8yPt2.

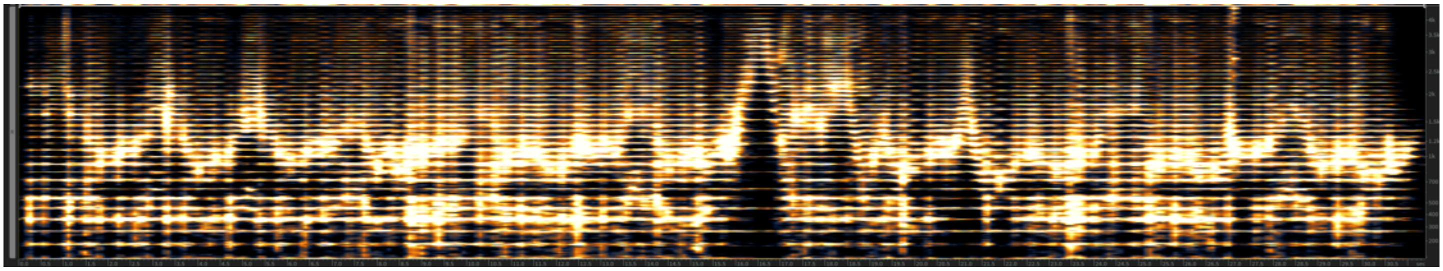

Fig.17: 4-part *framed duplum* texture of the bronze Nepalese *murchunga*.

12. Brass heteroglot JH: 4-part homophony

First of all, we should point out that brass heteroglot JHs produce completely different textural types (Fig.18) than brass idioglot JHs (Fig.13). For some reason (which most likely has to do with the geometry of the construction), heteroglot brass textures resemble copper in emphasizing the spectral content above the highest melody (>2.5 kHz). The entire upper portion of the diapason of heteroglot brass JHs is characterized by the increase in brightness and the reduction in differentiation of its spectral components – so that on spectrograms its entirety looks like a single homogenous "block" of an excited spectral material. Idioglot brass textures do not show such "massive" excitement of the upper portion of JH's diapason. A thinner and more flexible idioglot construction must be responsible for faster decay that contributes to the "staccato" look of Fig.13 comparing to Fig.18.

Brass heteroglot constructions generate textures similar to bronze. Mongolian *aman huur* provides an example of a texture that combines features of bronze Type-1 and Type-2 into **4-part framed figurative homophony** (Fig.18). What distinguishes this texture from bronze Type-2 is the subordinate function of its tenor part. If in bronze JH tenor's melodic shape opposed that of the primary melody of alto, here tenor and alto both share the same melodic contour – it is just that tenor's version is noticeably flattened and not always changes in phase. Along with its softness (in comparison with the alto melody), this suggests that tenor's melodic figuration is designed to accompany the main melody in alto. Melodic content of tenor is limited to a sheer pattern of 4 tones that resembles a "broken chord" – quite common figure of accompaniment in Western homophonic music (e.g., the so-called, Alberti's bass). Such patterning probably originates in the idea of embellishing the monotony of the drone. Melodic motion of soprano is also embellished with a stressed voice that includes occasional auxiliary tones, brought out from the otherwise homogenous chordal repetitions. Registers above soprano contain no discernible melodic motion. Each part receives dynamic prominence according to its textural function: the principal melody leads by a margin of 12 dB ahead of the second in importance figurative tenor, which in turn, exceeds both pedal parts (bass and soprano) by about 5 dB. Both pedals are dynamically about equal and stand out from the rest of the undifferentiated texture by approximately 11 dB. The tuning of all degrees remains within the harmonic series of F2. http://chirb.it/c9xzsG

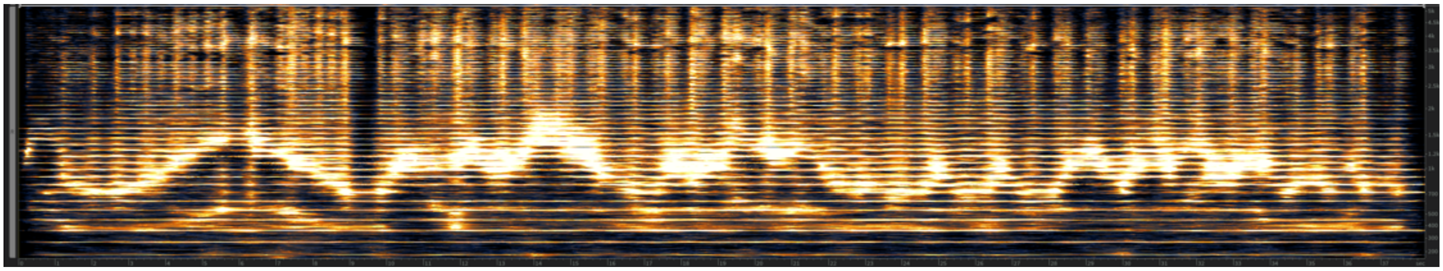

Fig.18: 4-part *framed figurative homophonic* texture of the brass heteroglot Mongolian *aman huur*.

1. Bass (73-291 Hz) – *pedal* tone ff=F2, where f3 (5th) is the strongest component (-46.3 dBu) (ditonic 5th) http://chirb.it/yh5xPr;
2. Tenor (304-628 Hz) – *melodic figuration* F4-A4-C5-Eb5 (-39.8 dBu) (7th-chord arpeggio) http://chirb.it/tOB3D5;
3. Alto (668-1976 Hz) – principal melody (-27.9 dBu) F5-G5-A5-B5-C#6-D6-E6-F6-Ab6 (diatonic heptatonic) http://chirb.it/Jvcb02;
4. Soprano (2064-4307 Hz) – *pedal* chord (-44.8 dBu) with occasional melodic auxiliary motion Ab7-Bb7-C7 (cluster) http://chirb.it/2h5k24.

13. Iron heteroglot JH: 4-part polyphony/3-part homophony

Iron and steel instruments' upper registers are as bright as those in copper, quite evident in spectrograms. But iron appears to push the melodic motion to a slightly higher register comparing to copper-based metals. As a result, tenor receives a wider space, which promotes filling it up with either its own melody or some form of

embellishment. Subsequently, iron textures can be quite diverse, and iron JHs often engage changes in texture within the same music work (like bamboo textures). Polyphonic textures of iron JHs are based on the same **4-part framed duplum type** that is common for copper alloys and bamboo. Homophonic textures of iron JHs resemble those of brass and bronze.

Type-1 heteroglot iron texture combines the features of both, Type-1 heteroglot bronze texture and heteroglot brass texture. Like bronze homophony, it features 3 parts, and like brass homophony it commits its tenor to the figurative melodic pattern. As a result, the leading melody finds its place at the top of this texture – according to the standard of multi-part homophony. This is quite remarkable, since many indigenous musical traditions that make use of this texture do not use multi-part homophonic textures in their traditional music at all. The example below (Fig.19) shows the transition between 2 textures: from **4-part framed duplum** texture (described below as Type-2) to **3-part figurative homophonic**. The latter should be considered homophonic since its alto greatly exceeds the tenor by its diapason, melodic diversity and tonal complexity. Also the arpeggio structure of tenor prevents it from forming easily trackable melody – usually melodies include at least a few "steps." The specifications below describe the homophonic texture. http://chirb.it/3gfImB (the provided audio clip illustrates the second portion of Fig.14). The tuning follows the harmonic series of the FF.

1. Bass (89-299 Hz) - pedal dyad ff=G2, where f1-f3 (5th) are the strongest (-39.4 dBu) (ditonic 5th) http://chirb.it/8p45Ox;
2. Tenor (293-814 Hz) – *melodic figuration* (-31 dBu) G4-B4-D5-F5-G5 (7th-chord arpeggio) http://chirb.it/HnrCv0;
3. Alto (831-2236 Hz) – *principal melody* (-32.2 dBu) A5-B5-C6-D6-Eb6-F6-F#6-G6-G#6-A6-A#6-B6-C7 (mostly chromatic) http://chirb.it/4mHNJF.

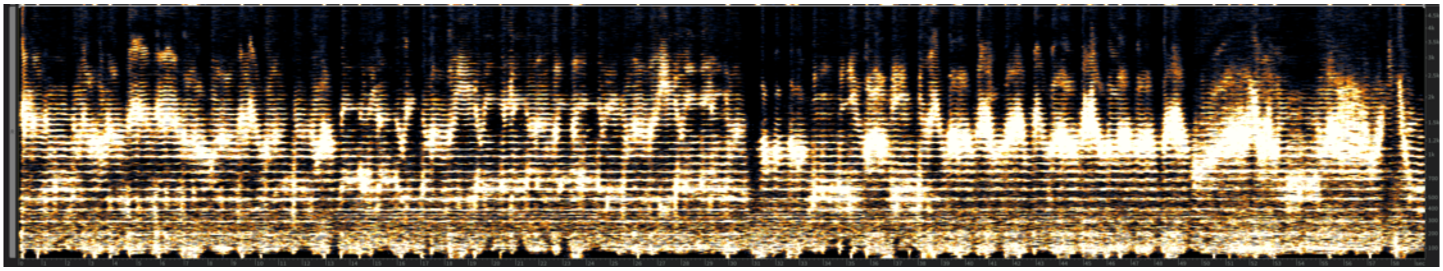

Fig.19: 3-part *homophonic* texture of the iron heteroglot Udege *kunkai* replaces 4-part *framed duplum* texture – 31 seconds after the beginning of the clip.

Type-2 iron texture (Fig.20) is equivalent to Type-2 bronze texture – based on the **4-part framed duplum** type, typical for bamboo. Its iron version retains the same diapasons and dynamic balance of near-equal strength of both melodies, giving a slight lead to the alto melody. Tonally, both melodies feature gaps and non-diatonic modes unusual for frequency-based music systems. The alto melody contains "false relations" between its degrees. http://chirb.it/xK26JM

1. Bass (67-332 Hz) – pedal dyad ff=Eb2, where f3 and f4 (4th) are the strongest (-60 dBu) (ditonic 4th) http://chirb.it/wwkmIw;
2. Tenor (385-1004 Hz) – secondary (little quieter) melody (-34.7 dBu max) G4-Bb4-Db5-Eb5-F5-G5-A5-Bb5-Cb5 (*gapped heptatonic*) http://chirb.it/sGzdwx;
3. Alto (1558-2318 Hz) – primary (little louder) melody (-33.8 dBu) D6-E6-F#6-G#6-A6-B6-C7-Db7 (*octatonic*) http://chirb.it/K9vr7O;
4. Soprano (2347-3602 Hz) – pedal chord (-51.7 dBu) with occasional melodic auxiliary motion D7-E7-F7 (cluster) http://chirb.it/K4mtm9.

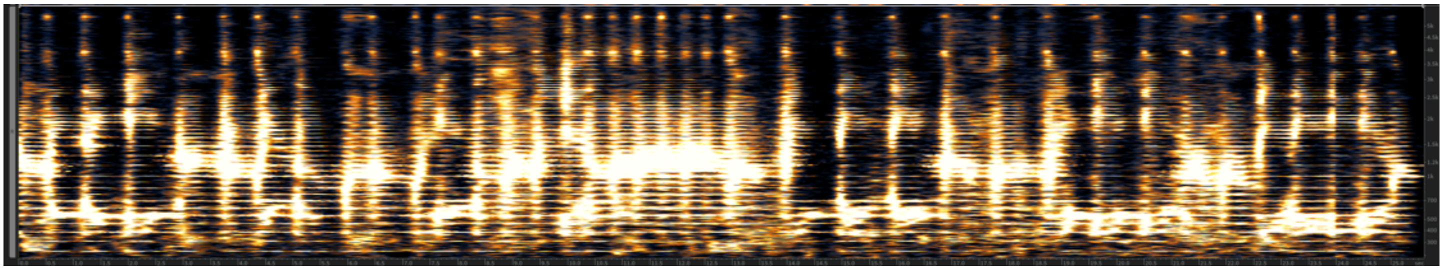

Fig.20: 4-part *framed duplum* texture of the iron heteroglot Nivkh *zakanga*. Its construction is nearly identical to the Udege *kunkai* as well as to the Tuvan *temir komus*.

14. Steel heteroglot JH: 3-part homophony

Textures of steel heteroglot JHs resemble textures of heteroglot copper and brass instruments in making the entire spectrum above the upper melody more intense, as compared to the bronze and iron heteroglot JHs, as well as the idioglot brass instruments. This increased brightness of the upper portion of their JH diapason makes the melody at the top of the texture much more salient, while reducing the differentiation between the textural components above the upper melody – thereby restricting the addition of polyphonic parts and simplifying the textures. As a result, heteroglot copper, brass and steel JH textures are characterized by homophonic arrangement of a single expressive melody. It seems that steel instruments are in particular well suited for emphasizing the melody at the top of the homophonic texture – judging by the commonality of such textures in JH music produced on steel heteroglot instruments. However, this impression might be due to a wider sampling pool, since steel is by far the most common material for recently manufactured JHs.

We call this most common form of homophonic arrangement **3-part superius chordal homophony** (Fig.21) and regard it as Type-1 of steel heteroglot textures. The term "superius" comes from the practice of music publishing in the 16th century, where it was reserved for designation of the highest part of a multi-part work. Hence, "superius homophony" refers to the "classic" model of homophony, where a single melody is placed "on top" of the accompaniment. Within the context of the JH texture, the term "chordal" specifies that the part that accompanies the melody is made of "chord-like" components of harmonics. An increased homogeneity of lower harmonics seems to characterize steel JHs as opposed to other alloys – and even more so as compared to the organic materials. As a result, the "chords" of tenor provide the most homogenous accompaniment – without stressing any voice inside the "chords" – quite similar to the standard chordal accompaniment in multi-part music (e.g., in marches). Steel instruments in general appear to have superior harmonicity, as a rule providing well-tuned harmonics. http://chirb.it/s1DBz3

1. Bass (51-304 Hz) – pedal tone ff=Db2, where f1 the strongest (-36.4 dBu) (monotonic) http://chirb.it/L94rKC;
2. Tenor (322-654 Hz) – pedal *chord* (-30.1 dBu) F4/Ab4/Cb5/Db5/Eb5 (9th-chordal anhemitonic) http://chirb.it/BBCLF9;
3. Alto (705-2329 Hz) - principal melody (-30.9 dBu) F5-G5-Ab5-Bb5-B5-C6-Db6-D6-Eb6-E6-F6-Gb6-G6-A6-B6-C7-Db7-D7 (chromatic) http://chirb.it/rrke6p.

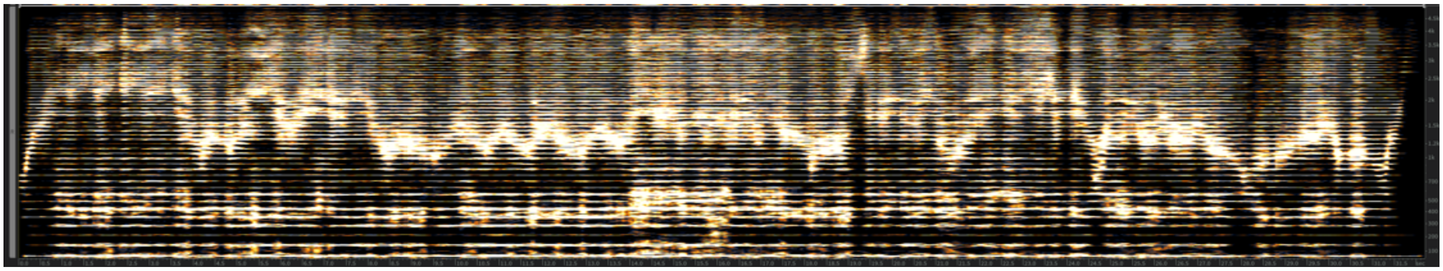

Fig.21: 3-part *superius homophonic* texture of the modern steel heteroglot Russian *vargan* (Glazyrin).

Type-1 is not the only textural type encountered in steel JH music. It seems to challenge, and perhaps exceed the variety of bamboo textures. The tenor accompaniment can be "melodic" rather than "harmonic" and contain a

melodic figuration. Such is Type-3 steel texture - **4-part framed ostinato homophonic** (Fig.22). In the example below this texture replaces (25 seconds after the beginning of the clip) Type-2: a very complex texture featuring **5-part tri-pedal homophony with a supporting voice**.

Switching from one texture to another is quite common for steel JH music. But in our experience all steel textures are usually homophonic, and their contrast is limited to registration and accompaniment choices – as in the example of Fig.22. It illustrates a special device - *khos yrya* (which means “2-part singing”) - of traditional Yakut khomus music, characterized by the ongoing opposition of melodic parts in a multi-part texture (I. Y. Alekseyev 1988). However, a brief look at Fig.22 shows that despite its name, *khos yrya* texture is not polyphonic but *homophonic*. It projects the impression of melodic opposition not by employing a genuine duplum polyphony, but by engaging a responsorial style consecutive opposition of two melodies, each of which is placed in a different register. Subsequently, their succession creates the impression of a conversation between “high” and “low” voices. Each voice receives a different form of accompaniment. The impression of switching to a lower level comes as a result of a greater dynamic emphasis placed on bass in section-2 (25-48 sec) and its deactivation of the soprano melody dub that was so pronounced in section-1 (0-25 sec).

It is worth noting that this deactivation demonstrates that JH player indeed exercises control over registral dubbing in a manner comparable to an orchestral arranger in Western classical music. In both cases dubs are engaged wherever a renewal of color is needed. The primary reason for using non-simultaneous textural contrasts seems to be that steel instruments trade greater sonority and precision of articulatory control for polyphonic capacities, making it hard for the performer to simultaneously track the bottom voice along with the upper voice. Presumably, the device *khos yrya* was invented earlier, before metallurgy was introduced in Yakutia, faceted on bone or wooden instruments that allowed for genuine polyphony. Then, reproduction of this device on a steel instrument presents the adaptation of an old traditional technique to the new medium – not unlike music compositions under the title “dialog,” quite typical in Western classical piano music (e.g., Tchaikovsky – Dialogue op.72 No.8), usually employ a homophonic emulation of the “polyphonic” idea of featuring two characters.

Both textures in the *khos yrya* example (Fig.22) retain the principal melody in alto, arranging it with the help of homophonic accompaniment. However, each texture does it in a different way – demonstrating impressive diversity due to the great differentiation of parts, available to the player on a high quality steel JH (especially if a player is masterful - as in the audio demonstration, below, by I.Alekseyev). The homophonic arrangement in Type-2 is quite intricate: there are 3 pedal parts that are registrally and tonally differentiated from each other. The bass pedal is “pointillistic” (a single pitch G#3), whereas the tenor pedal is a coloristic 7th-chord based on an augmented triad, and the soprano pedal is a chromatic cluster. The principal melody receives melodic support from the “satellite” descant part – above the soprano pedal. Despite its relative softness, this part is clearly marked – and its disappearance from the texture is immediately obvious at the point of 25 seconds of the audio clip https://chirb.it/OGn8Kr (Fig.22). This texture can be auditioned in separate from Type-3: http://chirb.it/KIAneB.

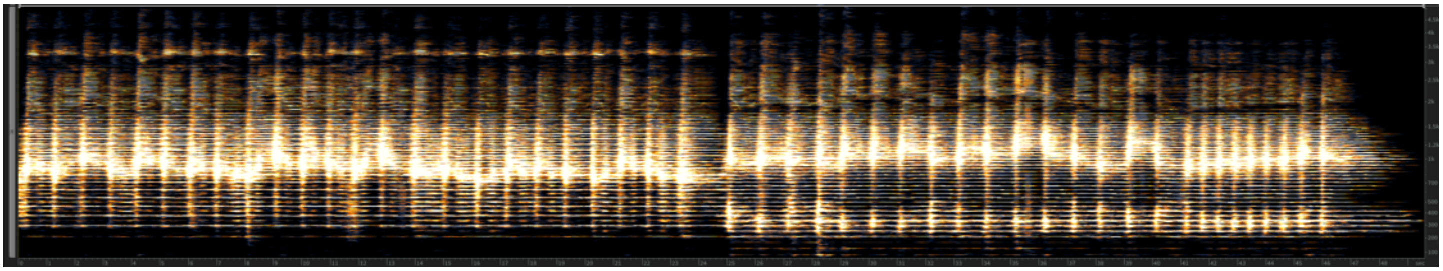

Fig.22: 4-part *framed ostinato homophonic* texture of the steel heteroglot Yakut *khomus* replaces 5-part *tri-pedal homophony with a supporting voice* – 25 seconds after the beginning of the clip.

1. Bass (30-210 Hz) – pedal tone, G#3 (f5 of the FF=E1), is amplified (-61.9 dBu) and isolated from the lower harmonics (16 dB difference from the second loudest lower harmonic) which are inaudible and barely detected by the frequency analyzer (monotonic) http://chirb.it/50yg6s;
2. Tenor (250-585 Hz) – *pedal* chord D4/F#4/A#4/C#5 (-36.9 dBu) (augmented 7th-chord) http://chirb.it/MI6FP6;

3. Alto (544-1800 Hz) – principal melody (-24.3 dBu) F5-G5-A5-B5(Bb5)-C6 (hemitonic pentatonic) http://chirb.it/2LDyew;
4. Soprano (1800-2927 Hz) – pedal chords (-58 dBu) (cluster) http://chirb.it/mpM5wP;
5. Descant (2960-3807 Hz) – marked supporting melodic voice G7-G#7-A7 (-59.2 dBu) http://chirb.it/csxLyf.

Type-3 switches on a completely different arrangement of the accompaniment. Bass receives the triadic pedal chord by activating f2, f3 and f4 (which were muted in Type-2). Tenor suddenly turns from harmonic into melodic part by featuring a melodic figuration made of 2 adjacent harmonics, f8 (E4) and f9 (F#4). The principal melody also changes by becoming more chromatic. Only the soprano pedal retains the same sound of a chromatic pedal cluster. http://chirb.it/sFC2M9

1. Bass (30-210 Hz) – pedal chord made of f2/f3/f4/f5, with marked f5 (G#3) (-53.8 dBu) which is 10 dB louder than the second loudest f3 (triadic) http://chirb.it/b0N72E;
2. Tenor (250-585 Hz) – *auxiliary melodic* figuration E4-F#4 (-31.5 dBu) (ditonic 2nd) http://chirb.it/xxnCpI;
3. Alto (544-1800 Hz) – principal melody (-27 dBu) Bb5-B5-C6-Db6-D6-Eb6-E6 (chromatic heptatonic) http://chirb.it/IJs8H9;
4. Soprano (1800-2927 Hz) – pedal chords (-56.5 dBu) (cluster) http://chirb.it/MAhMcf.

15. Stainless steel heteroglot JH: 3-part homophony

Although stainless steel does not substantially differ in its chemical composition from regular steel, it seems to carry its own “sonic signature,” judging from the comparison between the same model (“Black” D. Glazyrin) manufactured from regular steel (Fig.21) and stainless steel (Fig.23). Although for the stainless instrument the most common texture remains the **3-part superius homophonic** - the same as that of the regular steel, there are a few important points of difference. Stainless instrument places a greater emphasis on the melody – both, by comparative dynamics (the melody is 11 dB louder than the bass as opposed to 4 dB for the steel construction) and by wider diapason that houses more degrees. The frequency analysis reveals that the stainless instrument also widens the range of tenor, generating much richer “chord” that is dynamically more “mellow” than steel tenor (Fig.21). Subsequently, stainless melody is less challenged by the articulations underneath. That is why it can afford much richer “chords” – 7-tone chord in our example (Fig.23) – compared to 5- and 4-tone chord pads in tenor of the steel JH, which comes second in the use of the rich 9th-chord structures.

Therefore, we may conclude that introduction of stainless-steel Jaw harps in the XX century and its growing popularity, promoted even greater dominance of a single melody in JH texture than did steel instruments – focusing JH music on solo melodic expression. Noteworthy, the most common steel texture, 3-part superius homophony, features 13 degrees in our steel JH melody example (Fig.21) – greater than any other material. And the stainless-steel example below (Fig.23) beats it with even greater number of 17 degrees! No doubt, the growing popularity of stainless-steel JHs must have contributed to the penetration of Western style frequency-based homophonic thinking into JH traditions across the world. http://chirb.it/qqF6vz

1. Bass (38-231 Hz) – pedal tone ff=Db2, where f1 is the strongest (-47.3 dBu) (monotonic) http://chirb.it/1P6sqy;
2. Tenor (207-757 Hz) – pedal chord (-37.9 dBu) Db4/F4/Ab4/Cb5/Db5/Eb5/F5 (9th-chordal anhemitonic) http://chirb.it/etAN1C;
3. Alto (759-3585 Hz) – principal melody (-36 dBu) Ab5-Cb6-Db6-D6-Eb6-E6-F6-Gb6-G6-A6-B6-C7-Db7-D7-Eb7-E7-F7 (gapped chromatic) http://chirb.it/wG9hIL.

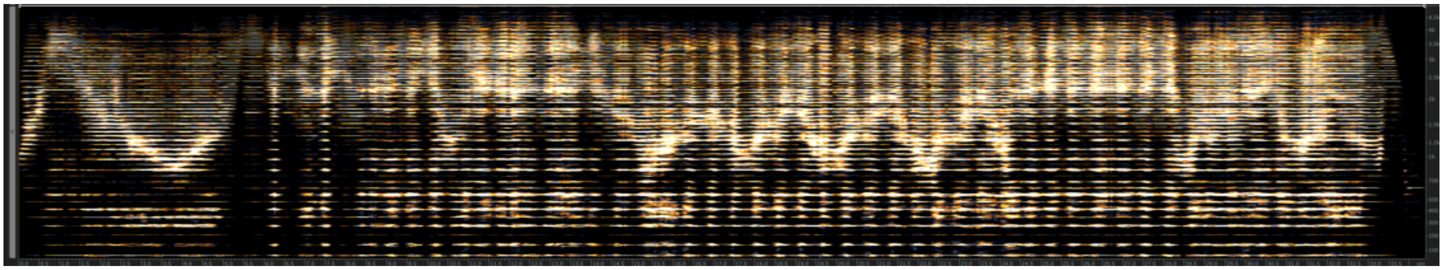

Fig.23: 3-part chordal homophonic texture of the modern stainless heteroglot Russian JH – the same construction (Glazyrin) as in Fig.21, only the steel is stainless rather than ordinary.

16. The comparison of copper, bronze, brass, iron and steel.

Industrial revolution opened doors to mass production of metallic artefacts. As a result of growing trade, many musical instruments became available to the general public, in multiple materials. Market economy incentivized manufacturers to explore the potentials of using different materials in order to achieve optimal balance between the acoustic quality of an instrument and its price. The incentive to increase profits pushed manufacturers towards making their products more affordable for target groups of population to increase the number of sales and win the competition with other manufacturers. This strategy typically coexisted with the opposite trend of maximizing the quality of an instrument to make it attractive to top players and their connoisseur audience – following the principle to sell less but for a higher price. Subsequently, the possibility of using new materials became explored in both directions – towards establishing the lowest margin beyond which the musical instrument sounded "bad" to its users, as well as the highest margin beyond which the enhancement in sound quality became insignificant compared to the increase in price. Through the interplay of demand and supply over a long period of time, manufacturers defined their product-lines by establishing an assortment of different versions of the same musical instrument, manufactured from different materials, to satisfy those market niches where the demand for a particular version stayed particularly high. Subsequently, those materials that earned high reputation within a particular market's niche adopted the role of a referential model for a particular instrument – as the discourse between that instrument's users reached some consensus about the connection between musical properties of an instrument and the material of its making. Once formed, such consensus often became internationalized due to the increasing globalization of trade.

Of course, not all "values" that have been established amongst consumers in relation to a specific product have necessarily a rational "materialistic" foundation (e.g., the belief that an instrument is the "brightest" does not necessarily mean that its tone indeed features greater intensity of the upper portion of that instrument's spectrum). Often "consumer values" reflect irrational beliefs that originate from sociological and ideological connotations of various musical instruments and materials. Especially pre-industrial societies are known to cultivate many "mythological" values related to musical instruments. Industrialization tends to increase the pragmatic aspect of consumption via its connection to marketing – mass produced products are usually advertised to satisfy a particular need by a specific feature. And market economy generally puts people's beliefs under scrutiny of commercial evaluation. Thus, people believe that silver and gold are "noble" metals. But do musical instruments made of such "noble" metals acquire a better tone compared to non-precious metals, to justify higher price? Market interaction between consumers and producers helps answer such questions. Commercial "probing" of the real value of this or that feature has led to debunk quite a number of "legendary" values of the pre-industrial past after the XVIII century – testifying to the power of consumer discourse in well-organized wide markets to verify and rationalize the pre-existing reputation of a product.

Evidently, some of traditional beliefs still survive even in modern times (Bacon 2004). However, all in all, the consensus between the end-users of a particular product definitely constitutes an important source of information for evaluating that product and its features. Amongst all musical instruments, the greatest variety of materials is used in manufacturing of percussive, brass and string instruments. The consensus amongst their users holds that copper is superior to other metals in "having better projection" of sound - which in acoustic terms means greater

radiation factor and smaller structural loss factor. High-tin bronzes and brass have the greatest acclaim for low damping.

String players believe that adding high-tin bronze winding to a steel string brightens and clears its tone, increasing its ringing – the greater the copper content, the warmer and darker the tone. Brass winding is believed to further increase brightness and jangliness of the string compared to bronze. And phosphor bronze, with its highest copper content (90%) is credited with mellower and darker tone than 80% copper bronze (Johnston, Simmons, and Ford 2005, 61–66).

Brass players believe that higher copper content in red brass (about 90%) contributes to warmer and mellower tone which is achieved by the cost of compromising the sound projection as compared to yellow brass (70% copper), known for its brightness and "cutting through." And gold brass (85% copper) occupies the intermediate position between red and yellow (Pyle 1998). Although the contribution of the material to acoustic properties of brass instruments has been questioned by experimental studies, which indicate that their connection is based on belief rather than facts (Bacon 2004), other studies suggested that the consensus of brass players, after all, might have rational ground. Thus, yellow brass was found to generate "crisp" transient attacks in contrast to "rounded" attacks of red brass, which explains the commonly made claim of greater projection power of high-tin copper alloys (Pyle 2009). Another experimental study has revealed that the sustained portion of the envelop of horn's sound indeed contains spectral variations depending on the material of horn's making – possibly, via blowing a lip-reed instrument that excites vibrations in the walls of the instrument due to the motion of the oscillating lips against the mouthpiece (Whitehouse and Sharp 2008). It is possible that the multi-faceted nature of timbre makes it hard to see how exactly the choice of this or that material for a particular musical instrument affects its acoustic properties – which specific aspect of spectrogram to look at? The spectral shape of the attack or sustain portions of a signal might be just one of a number of aspects to look into in this regard.

Perhaps, the best studied and most established is the contribution of the choice of material to acoustic properties in musical bells. Increased tin content increases hardness in alloy while decreasing tensile and impact strengths and reducing internal porosity that interferes with vibration (Nadolski 2017). Addition of tin to copper was experimentally found to improve the resonance of the bell, while addition of zinc reduced the sound's sustainability – which must explain the steady increase of the share of the tin in bell bronzes from Middle Ages to current time throughout Australasia and Europe (Audy and Audy 2008).

Cast iron has earned the reputation of high damping and low harmonicity when struck, producing a thump or clang – however, this did not prevent the development of the technology for using iron bells in China, starting from at least the XI century (Rostoker, Bronson, and Dvorak 1984). This is in sharp contrast to Europe, where for a long time ferrous materials were considered unmusical, so that their use for bell manufacturing started about 1857 (Strafford et al. 1996).

In relation to JH, the choice of materials has also passed the test of time – only those materials have been retained in use that proved to be capable of supporting the desired acoustic qualities. The overall shift from organic materials to metals is quite obvious in preferences of JH players throughout the modern world – even in the areas where JH traditions were bound to bamboo, bone or wooden instruments. Amongst all metals available for production only a handful has won acclaim: steel, iron, brass, bronze and copper (in the order of descending popularity). Gold, silver, zinc, tin, lead, aluminum, chromium, nickel have not earned recognition of JH makers and players despite their commonality for manufacturing of other objects. Evidently, the musical qualities of a metal must play an important role in JH users' preferences. Almost all heteroglot JHs are made of metal, usually forged out of steel or cast out of copper alloys, and geographically centered in Europe (Suits 2007). Idioglottic metallic constructions, on the other hand, are used primarily in Asia – which should be explained by the process of spreading the metallurgy technology from West to East of Eurasia, where idioglottic frame-shaped construction came as an emulation of the older tradition of making JHs from organic materials. Hence, the acoustic properties of metals used in production of heteroglot JHs in Europe must have influenced the preferential choice of specific metals. And judging by the archaeological finds of European JHs, 69% of the entire material constitute forged iron and 20% cast copper alloys (Kolltveit 2004). Clearly, ferrous and copper-based alloys are the favorable materials for the metallic JHs.

Unlike the organic materials, metallic materials form a reliable timeline in regards to the historic succession of their discovery and usage. Ronald Tylecote outlines the principal stages in mastering the technology of metal extraction and manufacturing across the globe in his monograph “A history of metallurgy” (Tylecote 2002). The first metal whose craft was mastered was pure copper (7). The earliest signs of its extraction were found in Catal Huyuk in Anatolia, dated by 7000-6000 BC (7), as well as in Rudna Glava and Ai Bunar, in the Balkans (14). The technology of extracting copper in a pottery kiln spread over the modern territories of Turkey, Iran, Iraq, Egypt, Bulgaria, Romania and Serbia Southern prior to the rise of first urban cultures – circa 4000-3800 BC (8-12).

This circumstance is most important for the transition from making JHs from organic materials to metallic ones, since JH belongs to the sphere of personal use by hunters/gatherers and nomadic pastoralists who keep migrating in small groups over extensive territory. The capacity of JH to mark specific territory by virtue of being made of a “kin tree” native to that territory and provide a way to integrate a JH player into a particular environment by imitating its sounds would be most valuable for those whose sustenance depends on finding provision at many different locations. Urban society precludes such lifestyle. Therefore, the chances of JH wooden or bone tradition meeting the wave of first copper-extracting technology are greatest at the periphery of the area of the earliest use of smelted copper – to the East of the Balkans and in the Northern vicinities of Iran, where JHs are still popular today (in contrast to the epicenter of metallurgy in the Near East, where no JH traditions exist).

The Bronze Age followed the Copper Age, quickly spreading from the same cradle area as for copper metallurgy, starting from circa 3000-2500 BC (Tylecote 2002, 25). Territories to the east of India (e.g., China) seem to have adopted tin bronze technologies during the Shang dynasty (1700-1027 BC), bypassing the Copper age (31). Therefore, it is likely that there the first musical textures of metallic JHs were introduced in the Far East region by bronze rather than copper instruments.

The Iron Age was next to follow. The knowledge of ironworking was incubated during the period 1500-1000 BC still in the same area of Anatolia and Iran (47). In the next 500 years it was spread over Europe and Asia, opening the Iron Age. The use of iron was first restricted to manufacturing weapons, while most objects were made from bronze. But gradually iron became more common – especially in China, where first cast iron cauldrons have been dated as early as 512 BC (44).

The first samples of steel were probably produced by accident as a byproduct of casting iron, where carbon could have been introduced either from ore or from charcoal. Since about 1100 BC steel blades have been occasionally made in the Middle East (51). Between the XI and VII centuries BC such blades became more common in the northern and western parts of Iran (52). Wootz steel was the first internationally renowned steel alloy invented in Southern India in the VI century BC and exported to the West – probably synonymous with “Damascus steel,” since Damascus was a famous trading post between East and West (56). In China the first steel sword was made during the Han period (206 BC - 24 AD) (57).

The first use of brass in the West occurred no earlier than about 30 BC, introduced in Egypt and quickly adopted by the Romans (57). Mastering of zinc technology could have occurred independently of western influences in China, where zinc is present in Han bronzes, produced after 220 BC. However, the copper-zinc alloys became common only after Han, after 220 AD (44).

Finally, the timeline of the metals employed for production of JHs comes to conclusion with the most recent invention of stainless steel. It was patented by Harry Brearley of Sheffield in 1914 as the medium-carbon steel with 12-14% of chromium (168). It is this alloy that has quickly earned international acclaim after Brearley co-founded the American Stainless Steel Corporation.

As evident from this brief overview, the history of metallurgy presents a clear-cut succession of the following materials that are commonly used for production of JHs (listed in chronological order):

1. copper,
2. bronze,
3. iron,
4. steel,
5. brass,

6. stainless steel.

In regards to acoustic quality of these materials, this transition reflects the search of an optimal balance between the most important acoustic qualities, durability and the manufacturing cost.

17. The summary of historic succession of the introduction of JH materials

At this point, we can summarize the order in which one material for making JHs succeeded another - following the model of Sheikin (2002, 132): by distinguishing between specific "stages" in mastering the technology of making a JH from a specific material. Mastering of the technology must have resulted in popularization of that material. We can now join the outline of historic development of metallurgy with the hypothetical outline of the succession of "stages" of mastering the organic materials, which we presented in the main paper:

1. grass (bark, chips),
2. bamboo,
3. wood,
4. bone.

Each of these materials constitutes a particular stage in the development of JH music – characterized by the discovery and exploration of new sonic attributes of JH sound, leading to standardization of a specific construction of JH and consolidation of the corresponding musical spectral texture(s). The progression of stages is cumulative – the onset of a new stage does not put an end to the previous stage, but "builds on it" by incorporating its technologies. Thus, "bamboo stage" follows "grass stage" by borrowing the method of picking a short plant and quickly preparing it for sound generation in JH manner. However, the spread of bamboo JHs does not terminate the use of grass JHs – in fact, Sakhalin JH traditions provide an excellent example of coexistence of "grass," "bamboo," "wood," "bone," "copper," "iron," "steel" and "brass" (Mamcheva 2012, 50–55, 156). This is quite similar to the evolutionary model of distinguishing between Copper, Bronze and Iron Ages. Iron Age does not terminate the use of bronze objects, which are kept in use in parallel to the newly introduced iron objects. For some objects iron becomes preferred to bronze in certain applications (e.g., swords), but in others bronze is preferred to iron (e.g., bells).

JH stages interact in a similar way, where JH textures that characterize certain materials might end up being adopted by a new stage, resulting in a modification of its texture, or compete with a new texture. Then, a new texture might drive the old one into oblivion (e.g., Mari metallic JHs supplanted JHs made of organic materials), or demarcate new boundaries between the parallel use of both textures, old and new, each within its own cultural niche (e.g., Mansi and Khanty parallel use of bone JH by women, and metallic JH by men).

The table below summarizes the typology of JH textures in the hypothetical order of their succession (Tab.1):

Table-1. The summary of textural typology of JHs made from different materials, in hypothetical chronological order of introduction of these materials. Altogether, there are 14 types of texture: 3 polyphonic 5-part, 1 homophonic 5-part, 5 polyphonic 4-part and 2 homophonic 4-part, and 3 homophonic 3-part types. Noteworthy, 3-part textures are all homophonic and are found only in metallic JHs, whereas 5-part textures are mostly polyphonic and mostly found in JHs made of organic materials.

Order of time	Material of making	Spectral texture type	Textural parts	Musical functions of parts	Bandwidth of parts (Hz)	Max. intensity (dBFS)	# of degrees in a part	Tonal genera of a part
1)	Grass	5-part 'scattered' quintuplum	bass	IIIa melody	50-175	-47.7	8	hemitonic
			tenor	I melody	205-686	-34.4	7	gapped hemitonic
			alto	IIb melody	701-1300	-45.0	5	gapped hemitonic
			soprano	IIa melody	1330-2467	-43.7	7	gapped chromatic
			descant	IIIb melody	2526-3757	-54.0	5	chromatic
2)	Bamboo	4-part melodic ostinato duplum	bass	pedal tone	10-210	-37.3	1	monotonic
			tenor	melodic figure	277-331	-36.0	2	ditonic 2nd
			alto	I melody	352-800	-31.0	5	anhemitonic
			soprano	II melody	772-2606	-34.7	11	diatonic
		4-part	bass	pedal tone	42-112	-56.3	1	monotonic

		chordal pedal duplum	tenor	pedal chord	200-630	-47.1	3	triadic
			alto	I melody	710-2517	-35.0	12	diatonic
			soprano	II melody	2833-3698	-54.0	4	chromatic
		4-part framed duplum	bass	pedal tone	61-330	-33.1	1	monotonic
			tenor	II melody	415-912	-20.4	4	gapped whole-tone
			alto	I melody	994-1991	-18.8	5	hemitonic
			soprano	pedal chord	2073-3399	-40.6	cluster	chromatic
		4-part chordal ostinato duplum	bass	pedal tone	50-255	-39.9	1	monotonic
			tenor	chordal figure	321-1141	-14.2	3 + 3	9th-chordal
			alto	I melody	1160-2320	-27.1	6 (7)	diatonic
			soprano	II melody	2324-3483	-39.4	4	diatonic
3)	Wood	5-part framed triplum	bass	pedal tone	63-210	-40.1	1	monotonic
			tenor	II melody	221-703	-39.0	4	anhemitonic
			alto	I melody	684-2310	-38.0	14	chromatic
			soprano	III melody	2470-3601	-59.3	7	chromatic
			descant	pedal chord	3634-5175	-64.5	cluster	chromatic
		4-part simple triplum	bass	pedal tone	62-267	-48.1	1	monotonic
			tenor	II melody	284-1054	-36.0	6	hemitonic
			alto	I melody	1115-2168	-27.4	9 (10)	chromatic
			soprano	III melody	2232-3407	-43.7	5	gapped chromatic
4)	Bone	4-part simple triplum	bass	pedal dyad	50-228	-35.6	2	dyadic 5th
			tenor	I melody	263-964	-32.0	5	hemitonic
			alto	II melody	990-1938	-36.3	7	chromatic
			soprano	III melody	1987-2980	-56.8	7	chromatic
		4-part simple triplum	bass	pedal chord	48-364	-39.2	3	triadic
			tenor	II melody	366-728	-35.2	7 (8)	hemitonic
			alto	I melody	730-1513	-34.9	8	diatonic
			soprano	III melody	1528-2378	-44.4	5	chromatic
5)	Copper	4-part framed duplum	bass	pedal tone	64-264	-57.3	1	monotonic
			tenor	II melody	326-783	-43.2	4	anhemitonic
			alto	I melody	848-3524	-40.1	5	chromatic
			soprano	pedal chord	3590-6332	-50.0	cluster	chromatic
6)	Bronze	3-part framed homophony	bass	pedal dyad	47-432	42.2	2	dyadic 5th
			tenor	melody	540-2807	-32.0	13	anhemitonic
			alto	pedal chord	2917-4213	-52.6	cluster	chromatic
		4-part framed duplum	bass	pedal tone	37-220	-48.4	1	monotonic
			tenor	II melody	270-631	-31.8	5	diminished 7th-chord arpeggio
			alto	I melody	727-3520	-28.4	11	"false" hemitonic
			soprano	pedal chord	3256-5236	-63.4	cluster	chromatic
7)	Iron	3-part figurative homophony	bass	pedal dyad	89-299	39.4	2	dyadic 5th
			tenor	melodic figure	293-814	-31.0	5	7th-chord arpeggio
			alto	melody	831-2236	-32.2	13	chromatic
		4-part framed duplum	bass	pedal dyad	67-332	-60.0	2	dyadic 4th
			tenor	II melody	385-1004	-34.7	9	gapped heptatonic
			alto	I melody	1558-2318	-33.8	8	"false" octatonic
			soprano	pedal chord	2347-3602	-51.7	cluster	chromatic
8)	Steel	3-part superius homophony	bass	pedal tone	51-304	-36.4	1	monotonic
			tenor	pedal chord	322-654	-30.1	5	9th-chordal
			alto	melody	705-2329	-30.9	18	chromatic
		5-part tri-pedal homophony with a 2nd voice	bass	pedal tone	30-210	-61.9	1	monotonic
			tenor	pedal chord	250-585	-36.9	4	aug. 7th-chordal
			alto	melody	544-1800	-24.3	5 (6)	hemitonic
			soprano	pedal chord	1800-2927	-58.0	cluster	chromatic
			descant	supporting voice	2960-3807	-59.2	3	chromatic
		4-part framed ostinato homophony	bass	pedal chord	30-210	-53.8	3	triadic
			tenor	melodic figure	250-585	-31.5	2	ditonic 2nd
			alto	melody	544-1800	-27.0	7	chromatic
			soprano	pedal chord	1800-2927	-56.5	cluster	chromatic
9)	Brass	4-part	bass	pedal tone	41-189	-70.8	1	monotonic

	(idioglot)	simple triplum	tenor	I melody	218-753	-49.0	7	diatonic
			alto	II melody	803-2313	-50.9	11	chromatic
			soprano	III melody	2361-3161	-62.1	5	chromatic
		5-part chordal framed duplum	bass	pedal tone	40-169	-49.7	1	monotonic
			tenor	pedal chord	173-307	-47.7	3	triadic
			alto	I melody	318-735	-37.6	7	chromatic
			soprano	II melody	758-2194	-40.1	9	chromatic
			descant	pedal chord	2201-3673	-60.8	cluster	chromatic
10)	Brass (heteroglot)	4-part framed figurative homophony	bass	pedal dyad	73-291	-46.3	2	dyadic 5th
			tenor	melodic figure	304-628	-39.8	4	7th-chord arpeggio
			alto	melody	668-1976	-27.9	9	chromatic
			soprano	pedal chord	2064-4307	-44.8	cluster	chromatic
11)	Stainless steel	3-part superius homophony	bass	pedal tone	38-231	47.3	1	monotonic
			tenor	pedal chord	207-757	-37.9	7	9th-chordal
			alto	melody	759-3585	-36.0	17	chromatic

This table reveals the general direction of the development of textural typology from polyphony to homophony through the process of increasing differentiation between parts and elaboration of different textural functions for each of the parts. The starting point was set by the "**grass stage**" texture, characterized by maximal polyphony and poor differentiation between parts - all melodic, and all moving simultaneously. Most likely, this texture was essentially "aleatoric," where only the loudest part, tenor, was consciously arranged by the player, leaving the other parts up to chance.

The "**bamboo stage**" introduced the differentiation of parts, related to the opposition of the monotonic bass and the ever-changing melodies of the upper parts. This development had to do with the physical properties of bamboo that featured much greater elasticity than grass leaves, and therefore supported a fixed pedal tone in the lowest part of the texture. All bamboo textures are based on the idea of varying the content of the part that is right above the bass pedal tone: turning it either into an autonomous melody, a triadic chord, a ditonic melodic figure or a pattern of alternating chords. Diversification of tenor is a logical strategy to reduce the monotony of the bass pedal which most likely appeared as a mistake to those JH players who were used to the "moveable" bass of grass JHs. The discovery of a "pedal effect" must have prompted the exploration of placing it not only in the lower portion of the texture, but also the top of it (as in "framed" textures). On the other hand, the overall low range and dark rattling sound of bamboo must have limited the number of parts to just 4, since the upper part of bamboo textures is never the loudest and has difficulty reaching the high range of JH, where the melody could be chromatic, providing contrast to lower diatonic melodies. Only one bamboo texture captures 4 chromatic degrees (chordal pedal duplum). Subsequently, bamboo textures are bound to duplum polyphony, where 2 parts diversify melody, and 2 other parts - pedal.

Wood overall tends to exceed bamboo in density and elasticity. As a result, wooden JHs engage more vibrational modes than bamboo, and activate higher registers. Greater brightness allows players to place important musical material in the highest parts. Therefore, they can fit 3 melodies into an available space. It seems that triplum polyphony became the centerpiece of arrangement of textures of the "**wooden stage**." Players must have gotten used to the monotony of the bass pedal and focused their attention on differentiating melodies by restricting them to different registers and thereby controlling their number of degrees and tonal genera. Contrasts in number of degrees and their anhemitonic, hemitonic diatonic and chromatic tonal relations allowed for the extension of the melodic polyphony up to 3 parts – the upper limit for musically untrained individuals.

Bone textures seem to adhere to the same principles of arrangement as wooden textures, introducing nothing principally new. The only noteworthy development is "thickening" of the bass pedal: if wooden textures all use a "pointillistic" pedal, bone textures engage dyadic and triadic pedal. Other than this sign of concern for tonal richness, bone JH players most likely kept using the same textures as wooden JH players.

"**Copper stage**" marks the return to the "bamboo stage" duplum style. Taking in consideration the fact that bamboo was totally absent in the continental Europe, we have to rule out the possible emulation of bamboo JH textures by the first proponents of copper JHs in the vicinities of Balkans. It could be that such interaction took place in the vicinities of Iran. Otherwise, the similarity of copper and bamboo textures might be a pure

coincidence. At any rate, the special importance of copper as a first metal makes its JH textures into likely models for early development of metallic JHs – especially for the Central Asian area, where the Copper Age was very distinct.

“**Bronze stage**” retains the framed duplum texture from the “copper stage” with the only difference that for some reason bronze melodies contain “false relations” – which might constitute an exception rather than a rule that characterizes all bronze JHs. However, bronze JHs feature another texture that marks the beginning of the new “Age of Homophony” – the simplest case of “framed 3-part homophony.” This texture is extremely easy to discern, since the melody is sandwiched between two pedals, one of which is dyadic (5th), and another – chordal (chromatic cluster). The registral and tonal contrasts in conjunction with the transparency of having only 3 parts make this texture much easier to handle for both, the performer and the listener, compared to any texture of the earlier stages.

“**Iron stage**” continues the legacy of the “bronze stage” by maintaining the same 2 types of texture: polyphonic and homophonic. The 4-part framed duplum of iron is indistinguishable from bronze – even the inharmonicity of some degrees is retained. However, the homophonic texture has a few important transformations. The melody is moved to the top part, whereas tenor houses a melodic figuration that consists of 4 “pitches” that comprise a 7th-chordal relation. This arrangement might reflect an attempt to reconcile both texture types: melodic figure in homophonic texture resembles the second melody in polyphonic texture – what sets them apart is the complexity of the polyphonic melody (9 degrees). Otherwise all lower 3 parts in both iron textures “behave” in a very similar way. The increase in their similarity marks an important tendency brought in by the “iron stage” – JH performers must have become aware of the difference between homophonic and polyphonic arrangement styles, whereas during the “bronze stage” they probably kept using the polyphonic texture by “cultural inertia” – just carrying on its tradition from earlier “copper stage” without relating it to the novel bronze homophonic texture.

“**Steel stage**” seems to have dropped polyphonic textures altogether, focusing exclusively on elaborating the variety of homophonic textures. Its most typical variety coins the archetype of “superius” homophony – the melody placed at the top of the texture, accompanied by chords underneath it. This texture is much simpler than the figurative homophony of “iron stage,” with which “superius” shares the same uppermost position of melody. The steel melody remains unchallenged by any alternative melodic motion. And as compared to the bronze “sandwiched” melody, the “superius” melody demonstrates the tendency to further simplification: it effectively merges both pedals, bass and tenor, together, so that only two entities in this texture are the de facto – very expressive, changeable and rich melody (18 degrees) set against very constant and rich chordal padding (9th-chord). Noteworthy, both textural entities here are rich. This signifies the tendency to embellish the musical expression – very possibly as a result of growing concerns for aesthetic value of musicking. The homophonic model of concentrating the musical expression in a single melody that becomes the carrier of the principal compositional idea of the entire music work reorganizes all steel textures. Its 4- and 5-part textures still observe only one melody and use the other parts to “dress up” this melody in the fanciest “clothing”. All inventiveness in steel textures aims at the differentiation of the accompanying parts.

The fundamental opposition between the JH textures of organic and metallic materials becomes completely clear only during the “steel stage.” All organic materials generate polyphonic JH textures. Copper, bronze and iron generate both, homophony and polyphony. Only steel specializes exclusively on homophony. It reserves enormous space for a single melody, enabling composition of more expressive melodies. Moreover, a wide diapason in a harmonic series allows a melody to engage anhemitonic, hemitonic, diatonic and chromatic intervallic typologies, thereby contributing to its diversity. In case of building rich textures, steel instruments rely on precision of registral control – this appears to be a feature that sets steel JHs apart from the earlier stages. By placing a particular textural device (e.g., a triadic “chord”) in a particular register, the player obtains power to tonally color it in such a way that secures the greatest differentiation between all textural components and thereby generates the maximal richness and diversity within the texture. This is how I. Alekseyev colors the pedal 7th-chord with a strange “augmented” tint – he places the “chordal” component on such harmonic in the harmonic series that generates the refreshing sound of D/F#/A#/C#. It looks like organic materials are simply incapable of supporting such concision of placement an array of textural elements (“chords,” “double-notes,” “melodic motifs” as well as their patterns) in a specific spot within the harmonic series.

Organic materials are prone to having polyphonic textures because their FF is relatively louder in relation to the other parts than in the metallic JHs. Basses in JHs made of organic materials are about 15 dB louder than basses in metallic instruments. This increases the emphasis on the bottom of the texture and directs the performer towards trying to overcome monotony of the bass pedal. And placing the melody right above the bass is the most contrasting design possible. However, having a low melody makes it inevitable for JHs made of organic materials to include yet another melody further above. The option of using "chords" in the upper registers exists only for the soprano range circa 3 kHz and is limited to chromatic clusters. Muting all the parts above the low melody does not seem to be possible on JHs made of organic materials.

On the other hand, as a rule, steel JHs feature significantly higher concision in tuning than JHs made of organic materials – their harmonics are tuned much closer to the natural values of the harmonic series and their bandwidth is noticeably narrower than that of bamboo, wooden and bone JHs. At the same time steel instruments are much more sensitive to the slightest variations in striking and breathing – much more so than non-metallic instruments. In our experiments, all 3 JH players who cooperated in our investigation could produce sound without engaging their hands – only through breathing, and only on metallic instruments, which exactly reflects their increased sensitivity. And out of all metals, steel seems to contribute to JHs the greatest homogeneity of lower harmonics that allows to produce very balanced "chords," comparable to chords of the organ or the mouth organ. Perhaps, it is this chordal uniformity that has prompted players of steel JHs to explore homophonic textures.

As we have already explained, brass JHs occupy a special position compared to other metallic JHs, because of the great malleability of brass that enables easy production of frame-shaped idioglot instruments without resorting to a blacksmith. Therefore, such JHs are often used as a replacement of bamboo or wooden instrument within the old indigenous tradition. According to the testimony of JH players, brass is easier to play and provides better control than the organic materials (Mamcheva 2012, 50). Hence, brass idioglot textures merely reproduce bamboo (duplum) and wooden (triplum) textures. In contrast to polyphonic idioglot brass JH, brass heteroglot variety seems to be homophonic – like other metals. Its 4-part framed homophonic texture with a melodic figuration in tenor closely resembles 3-part iron homophony, differing from the latter only by addition of an extra chordal part over the melody.

And stainless steel features exactly the same 3-part superius homophony as steel JH. So, "**brass stage**" and "**stainless steel stage**" neither introduce any new textures, nor make principal modifications to the pre-existing textures. "Steel stage" provides the last important contribution to the evolution of JH textural typology by breaking away from the traditional forms of JH polyphony.

18. How the mechanical properties of JH made of different materials correspond to their respective textures

The entire evolution of JH music seems to be determined by the succession of "stages" in emergence of different materials of which "bamboo," "wood," "copper," "bronze, "iron" and "steel" stages were the most important in the development of JH's textural typology.

These "stages," in turn, clearly form groups based on the general similarity of their corresponding textures and the similarity of mechanical properties between the materials introduced by each stage. The correspondence between both groupings is remarkable. Metals generally contrast organic materials with their physical properties – so does homophonic predisposition of metallic spectral textures versus polyphonic predisposition of the textures of organic materials. Bamboo, bone and wood form a group of materials that remain quite close in their physical properties – in contrast to grass leaves (Tab.2). And, accordingly, the corresponding textures also break into 2 classes: grass textures present "aleatoric" polyphony that is maximized above the perceptual limit and is undifferentiated, while bamboo/wood/bone textures are "rational" in their polyphonic design: they assign specific textural functions to specific parts, which make parts differ from one another by featuring different structural properties. As a result, bamboo/wood/bone JH music becomes free of "aleatoric" flavor – which is most obvious if to listen to a long piece of bamboo JH music versus grass JH music. Despite the two plants, Bambusa (e.g., in

mukkuri) and Leymus (in *koka chnyr*) belonging to the same botanical family of Poaceae, their physical properties drastically differ due to the exceptional tensile strength of bamboo.

For sound applications, the most important acoustical properties of materials have been established by generations of makers of musical instruments in their attempts to enhance their construction. These acoustic properties have to do with radiation of sound – duration (time), brightness (frequency), fullness (timbre), projection (directionality) and loudness (amplitude). In turn, these properties are determined by the mechanical properties of the materials used to generate musical sound: density, elasticity and damping coefficients (Norton and Karczub 2003, 717). The entire evolution of percussive organology demonstrates that there are objective boundaries for the ideal "warm" sound – musical cultures usually define the approximate ranges for the model sound – e.g., a density of 0.80 to 0.95 g/cm3 and a Young's modulus (elasticity) of 15 to 20 GPa (Holz 1996). JH sound is bound to the same "modelling" upon some culturally accepted reference of a "good" tone that can be defined by density (***ρ***), tensile elasticity (***E***), specific acoustic impedance (*z*), sound radiation coefficient (***R)*** and loss coefficient (**η**).

Young's modulus (***E***) describes tensile elasticity (MPa) - the tendency of an object to deform contrary to the axis of the applied force:

$\boldsymbol{E} = \sigma / \varepsilon$ [the ratio of the uniaxial force per unit surface to the proportional deformation]

Density modulus (***ρ***) describes the mass of a substance per volume (kg/m2):

$\boldsymbol{\rho} = m / v$ [the ratio of the mass to the volume]

The heavier a material, the higher its density and the more energy it takes to move its molecules. The lower the density, and the higher the elasticity – the faster the speed and the shorter the duration of the sound. Elastic properties have a greater influence on the wave speed than on density properties. The higher the elasticity, the higher the natural frequency (eigenfrequency) of the material, so that the sound is shorter but brighter. On the other hand, the higher the density, the lower the natural frequency (the longer the sound but darker).

Specific acoustic impedance (*z*) describes the capacity of a particular material to transmit sound which determines the efficacy of the sound system (Pa*(m/s)):

$z = \rho / v$ [the ratio of the sound pressure to the particle velocity]

The higher the impedance, the lower the speed, making the eigenfrequency lower. Impedance depends on elasticity: the higher the elasticity and the density, the higher the *z*. Unlike elasticity, the *z* depends on the phase and is usually complex (there are multiple impedances in different locations at the sounding body). Unlike elasticity, impedance also depends on frequency. The interaction between phase and frequency sets the resonance frequency at eigenfrequency. The proximity to eigenfrequency amplifies the partials – whereas those partials that fall in between the eigenmodes are not amplified (Angster and Miklós 2018). At eigen-resonance points there is a high input impedance. Yet another important aspect of the *z* is that standing waves occur exclusively at resonance frequencies. The smaller the impedance, the less force is required to stimulate a certain velocity. Magnitude and phase of the impedance that curve at resonance frequencies influences timbre (Kausel 2018).

Sound radiation coefficient (***R)*** or radiation factor (***σ***) describes how much of the vibration energy of a body is lost by radiation to the surroundings (radiation efficiency):

$\boldsymbol{R} = v / \rho$ [the ratio of the particle velocity to the sound pressure]

The higher the radiation factor, the more of the vibration energy is transmitted to the air, and the faster the sound decays. High radiation coefficient in conjunction with low impedance benefit the sound transmission from the sounding body to the air_(Niklas and Spatz 2012, 319). The higher the speed, and the lower the density – the higher the ***R***. ***R*** depends on the frequency and the dimensions of the vibrating body. The higher the frequency, the higher the speed and the ***R***. Metals feature an increase in radiation above 2 kHz, whereas woods – a decrease (Waltham and Yoshikawa 2018), which must be responsible for greater sonority and brightness of metallic materials combined with their significant decay in high frequency range.

Of special importance is the critical frequency (or, "coincidence frequency") – at which the phase velocity of vibrations in the sounding body of a musical instrument matches the speed of sound in the air (343 m/s). Below the critical frequency, standing waves in the sounding body remain inefficient radiators of sound. However, once the phase velocity surpasses the speed of sound in the air, the corresponding standing wave starts radiating rather

efficiently, generating the energy flow from regions of positive to negative vertical displacements and vice versa (Gough 2014). High radiation is particularly important for JH – responsible for transmitting a greater number of vibration modes to the mouth cavity, thereby increasing the number of active harmonics and expanding the diapason of the spectral texture.

Loss factor (η) expresses the degree to which a material dissipates vibrational energy by internal friction. This determines for how long the structure sustains vibration. This is strictly a material property that does not depend on variation of physical attributes of the sound. The higher the **η**, the faster the sound decays – and the contribution of it is more important than radiation losses. Studies of time decay in wooden xylophone bars without resonators have shown that the decay process is mainly determined by internal losses (Fletcher and Rossing 1998, 637).

The table below reflects the mechanical properties of metals, wood, bone, bamboo and grass leaves in reference to a typical nylon string (Tab.2).

Tab.2. The mechanical properties of the materials used to make JHs in reference to the nylon string. Since the mechanical properties of Leymus are unknown, the values for canegrass leaves (of the same botanical family) are used instead. For orthotropic materials (wood and bone) E refers to E_L, G to G_{LR}, and c to c_L. E_P and c_P mean accordingly, elasticity and speed of the vibrations "perpendicular" to the direction of the sound wave (E_T – tangential - for wood). Spruce was selected to represent softwood, and oak – hardwood. Moso bamboo was selected to represent the Far East area, where JHs are very common. Mean values are enclosed in square brackets.

Material	Density ρ (g/cm^3)	Young's modulus E_L (GPa)	Young's modulus E_p (GPa)	Shear modulus G (GPa)	Sound speed c_L (m/s)	Sound speed c_P (m/s)	Structural loss factor η_s	Characte-ristic z (Pa*s/m)	Radiation coefficient $R=c_L/\rho$
Nylon string	*1.1* [a k]	*0.5* [a]			*2620* [a j]	1070 [j]		0.3 [aa]	2.4 [aa]
Stainless steel 304	7.9 [c j] 8 [k]	193 [c k] 195 [j] [194]		75 [c]	5000 [j] 5050 [h] 5790 [m] [5395]	3100 [j m]	0.001 [L]	43.1 [aa]	0.7 [aa]
Carbon steel A36	7.7 [a b] 7.9 [k] [7.8]	195 [b] 200 [c] 207 [k]		83 [b] 75 [c]	5050 [b h] 5100 [i] 5180 [j] [5115]	3100 [L] 3220 [j]	0.0001–0.0006 [e] 0.00002–0.0003 [i] 0.0009-0.014 [L]	39.0 [b]	0.7 [aa]
Cast Gray iron 1800	7-7.4 [j k] 7.7 [b] [7.4]	105 [b] 66-97 [k]		44 [b]	3700 [b] 4500 [e] 4480 [j] [4100]	2809 [j]	0.001 [e] 0.0001–0.0004 [i] 0.0019-0.016 [L]	28.5 [b]	0.6 [aa]
Brass C3600 (yellow)	8.5 [b d k] 8.4-8.8 [j] [8.6]	104 [b] 100 [j] 97 [d k]		38 [b] 37 [d]	3500 [b h j] 3200 [i] [3350]	2100 [i] 2110 [j]	0.0002-0.001 [i] 0.003-0.006 [L]	29.8 [b]	0.4 [aa]
Bronze C2400	8.7 [d] 8.7-8.9 [j] [8.8]	110 [d]		40 [d]	3300 [e] 3530 [m] [3415]	2230 [m]	0.00015-0.01 [L]	30.0 [aa]	0.4 [aa]
Copper C11000	8.9 [b d k]	122 [b] 115 [d k]		44 [b d]	3700 [b h l j] 4700 [m] [4200]	2300 [i] 2270 [j] 2260 [m]	0.002 [e l] 0.0001-0.003 [L]	33 [b]	0.5 [aa]
Bone, cortical human (de vitro)	1.7-2 [q s] [1.8]	6.3-18 [q s] [12.2]		2.2-7.2 [s] [4.7]	3009-3330 [q] [3170]	1452-1946 [1699]		7.7 [t]	1.8 [aa]
Oak (Quercus)	0.61-1 [b j l k w] [0.8]	5.3-14.1 [k] [9.7]	0.55-0.97 [k x] [0.8]	0.75-1.18w [1]	1500-4000 [b i] [2750]	1109-1387 [i] [1248]	0.01 [i]	2.9 [b]	3.4 [aa]
Spruce (Picea)	0.44-0.7 [c g h j] [0.6]	9.7-15.9 [c h n] [12.8]	1.3 [h] 3.9 [x] [2.6]	1.1 [g]	5100 [g] -5200 [h] [5150]	1700 [h]	0.01<2kHz [h]	3.1 [aa]	8.6 [aa]
Bamboo Bambusa vulgaris	0.59-1.38 [a g o] [1]	3.6-20 [n o] [11.8]		1.3 [g]	4600 [g]		0.01-0.02 [o]	4.6 [aa]	4.6 [aa]
Bamboo, Moso (Phyllostachys)	0.6-0.9 [p r] [0.8]	7.7-15.2 [p r] [11.5]	0.3-0.6 [p] [0.45]	1.6 [r]	3791			3.0 [aa]	2.6 [aa]
Canegrass, leaf (Eragrostis)	0.0045 [v]	1.05 [u]			15275			0.07 [aa]	3513 [aa]

a The values are given by Wegst (Wegst 2008).
aa The values are calculated according to Wegst (Wegst 2008): $z = c_L \times \rho$ and $R = c_L / \rho$ – using the mean values.
b The values are given by Kinsler (Kinsler 2004).
c (Hibbeler 2014).
d (ASM International Handbook Committee. 1990).
e (Norton and Karczub 2003).
f The values are given by Li (Li 2004).

g (Waltham and Yoshikawa 2018)
h (Fletcher and Rossing 1998)
i (Cremer, Heckl, and Petersson 2005)
j The values are given by Haynes, and the densities of irons, brasses and bronzes were provided in ranges (Haynes 2014).
k (Callister and Rethwisch 2014)
l (Zhang, Perez, and Lavernia 1993)
m ("Standard Practice for Measuring Ultrasonic Velocity in Materials" 2002)
n (Awalluddin et al. 2017)
o (Janssen 1991)
p (Li 2004)
q (Eneh et al. 2016)
r (Kubojima et al. 2010)
s (Natali and Meroi 1989)
t (Weiss et al. 1998)
u (Balsamo et al. 2006)
v (O'reagain 1993)
w (Sliker and Yu 1993)
x (Bucur 1995)

The table above demonstrates that in relation to **density** all materials break into 3 groups (in increasing order):

1) grass (0.005),
2) wood/bamboo/bone (0.4-2), and
3) ferrous/copper-based metals (7.7-8.9).

In relation to **elasticity,** all materials form 4 groups:

1) grass (1.1),
2) bone/bamboo/wood (3.6-20),
3) all metals except steel (66-122), and 4) steel (193-207).

In relation to the vibration **velocity,** all materials form 3 groups (in decreasing order):

1) grass (15275),
2) steel (5000-5790) and spruce (5100-5200),
3) other metals and organic materials (1500-4700).

In relation to the **radiation factor,** all materials form 4 groups:

1) grass (3513.0),
2) wood/bamboo (2.6-8.6),
3) bone (1.8) and
4) metals (0.4-0.7).

In relation to the **characteristic impedance,** all materials form 5 groups (in increasing order):

1) grass (0.1),
2) bamboo/wood (2.9-4.6),
3) bone (7.7),
4) metals except steel (28.5-33.0),
5) steel (39.0-43.1).

In relation to the **structural loss factor,** we only have the data for metals, bamboo and wood. Metals seem to have considerably lower loss factor than bamboo and wood. This corresponds with their greater capacity to sustain sound and therefore provide better "resolution" for the details in the arrangement of textural parts in the upper range of JH's diapason where decay is the strongest.

The other mechanical properties also show correspondences with the characteristic features of textural arrangements marked above. The grouping of the materials in relation to their density, elasticity and impedance all generally coincides - and matches the historic development of the textural typology, outlined above. The increase in elasticity provides a general increase in bandwidth (fullness), amplitude (loudness) and the number of vibration modes (timbral richness and brightness) in the sound of JHs made of more elastic materials. The increase

in density reflects the longer lasting sound of JH. The higher radiation factor contributes to faster dissipation of energy and greater decay. These regularities totally agree with the textural typology. On one pole we have grass JHs whose sound is the shortest, bearing strong staccato quality and providing the poorest differentiation between parts. On the other pole we have metals that sustain the sound much longer, provide superior “resolution” of details in textural arrangement and support higher harmonicity.

Velocity is a bit trickier. It is the highest for grass, reflecting its nearly absent capacity to sustain sound. Higher velocity of metals (especially steel) is combined with low radiation factor, thereby enabling the greater portion of JH spectrum to exceed the speed of sound in the air and sustain longer – contributing to brighter sound and allowing the melody to be placed lower in texture, as well as supporting a higher position of chords in the JH diapason.

The higher impedance reflects the resonance properties of the material: together with its dimensions it determines the eigenfrequency – the higher the impedance, the lower the eigenfrequency, and subsequently, the stronger the harmonics proximal to that eigenfrequency. This factor must be responsible for the capacity of steel JHs to emphasize the textural parts in the lower mid portion of the JH diapason and bring out their detail – especially the “fullness” and “evenness” of chords.

In relation to gradations in harmonicity and detailing of the textural material, the dividing line separates metals from non-metals: metallic textures favor homophonic arrangement with its increased importance of harmonic relations between the textural components; whereas non-metallic textures favor polyphony that relies on melodic rather than harmonic aspect of tonal organization. From mechanic perspective, this opposition corresponds to the opposition of isotropy to anisotropy. Wood’s elastic and strength properties are dependent on the direction of the applied force with respect to the grain in wood’s fibers. Elastic properties of the wood substantially differ across and along the grain – wood is a strongly anisotropic material.

Anisotropy constitutes the biggest factor in the acoustic properties of JH materials. The differences in elasticity, velocity, impedance and radiation all fall into two clusters: organic and non-organic metallic materials – along the axis of anisotropy. The transverse Young's modulus of bamboo and wood is between only 1/20 to 1/10 of the longitudinal Young's modulus, and consequentially the speed of sound across the grain is only approximately 20% to 30% of the longitudinal value (Wegst 2008). Similar extent of anisotropy is observed in shear modulus and in the Poisson’s ratio (Fletcher and Rossing 1998, 721). Axial strength of wood may be up to 50 times greater than its transverse strength (Tsoumis 1991). Bone is about as anisotropic as wood: the ratio of Young's modulus to shear modulus is of the order of 20:1 for cortical bones of mammals and birds (Spatz, O’Leary, and Vincent 1996).

On the other hand, metals are regarded **isotropic**. In reality, they are not perfectly isotropic: typically, their longitudinal elasticity is about twice greater than transverse. What this means is that the vibrational modes of different axes in the material do not form perfect harmonic relations, and the longitudinal vibrations exceed the transverse in their frequency by the factor of 2 for metals, and 10-20 for wood and bone. The factor of 2 acoustically transpires in the ratio of an octave: the frequency of longitudinal vibrations stays about an octave higher than that of transverse vibrations – equal to the frequency interval between f1 and f2 of the harmonic series. Although the anisotropic factor in metal is most often inharmonic, in registral aspect, partial vibrations in both domains, longitudinal and transverse, merge together much better than those of wood/bone materials, where their intervallic relation approximates the interval between f1 and f10 and, possibly even greater - up to f20. Huge registral distance is likely to segregate frequencies of the same vibrational mode between both domains, breaking them in two perceptual entities. That is why we usually hear a distinct “clang” pitch upon hitting a wooden bar, which is distinctly different from the sustained pitch in the aftermath of the initial “clang.” In contrary, the near-octave relation between the partial vibrations of both domains in metallic beam enables merging of both frequencies into a single complex tone. This difference is even more pronounced for JHs since the primary mode of its vibration is transverse – along the direction of the strike. Hence, longitudinal and torsional vibratory modes are likely to constitute a smaller share in the spectral content of a tone of metallic JH, compared to bone and wooden ones. Three important ramifications of this are:

1. Tonal qualities of different samples of the same construction in metallic JHs have greater uniformity than in bone/wooden instruments, which are prone to significant discrepancies due to even the slight changes in orientation of the instrument's planar surface towards the grain structure. Musically, this transpires into the likelihood of formation of conventions of tonal organization of music produced on metallic instruments. That is, adoption of metallic instruments by a culture is likely to push it in the direction of establishing the preferences for certain musical structures and rules of their usage based on their harmonicity. The archeological date of mastering a metallurgical technology for that culture then marks the onset for standardization of musical texture for JH music.
2. The tone of a metallic instrument bears a closer adherence to a single harmonic series model than the tone of bone/wooden instrument. Better merging of transverse and longitudinal frequencies is likely to direct the attention of JH users to the harmonic structure of a tone. Switching from bone/wooden instrument to a metallic one is likely to reveal the novel and peculiar aspect of a more harmonious "wholeness" in the sound of JH, encouraging some kind of "chordal" musical thinking. Extended use of metallic JHs is likely to promote homophonic principles of constructing musical texture based on "just intonation," where the JH player perceives the tone as a complex of harmonic elements estimated by means of "harmonic" increments within a single harmonic series.
3. The tone of a JH made of strongly anisotropic material is likely to promote not "vertical" but "horizontal" thinking that would explore and highlight the differences between the longitudinal, transverse and torsional vibratory modes. Inferior homogeneity of harmonic properties for each of these domains transpires into a trend that is opposite to metallic JH texture-making. Anisotropic textures possess greater timbral discrepancies between different registers, which allows for greater contrasts in unveiling a specific textural material in one register as opposed to another. This encourages JH players to come up with polyphonic textures that employ tonally contrasting parts.

Most of forms of biomass are anisotropic, like wood – moreover, most plants also have tissues or structures that have orthotropic mechanical properties – properties that differ along 3 mutually orthogonal axes of rotational symmetry (Dahlquist 2013, 348). This is because plants typically grow vertically upward (orthotropic growth), towards sun, while their lateral sides grow at more horizontal angle (plagiotropic growth). Orthotropy greatly influences propagation and polarization of shear waves, increasing their difference from that of longitudinal and quasi-longitudinal waves and generating also fast and slow shear waves (Bucur 2016, 114–22). Bamboo is also orthotropic: its longitudinal axis is defined as the axis parallel to the fibers (similar to grain of wood) and thus along the length of the bamboo culm or tree stem; its radial axis is the axis perpendicular to the culm's (stem) circumference; and the tangential axis is the axis perpendicular to fibers and culm or stem, while being tangential to the circumference of the tube (or stem) (Wegst 2008). Bone also shares orthotropic features with wood, showing the remarkable similarity of the micro- and ultra-structural aspects between both (Lakes 1993). This has to do with the vertical direction of growth of many vertebrates, designed to overcome the force of gravity. Therefore, metallic JHs constitute a class of musical instruments radically different in their pattern of propagation and polarization of sound waves inside the sounding body, as compared with JHs made of organic materials – despite the superficial resemblance of their constructions.

As we see, the principles that we have inferred from textural analysis of JH music are rooted in the mechanic properties of the materials used to make them. Moreover, these properties are likely to influence the cultural status of a JH manufactured from a particular material. Thus, the ease of making a grass JH is combined with a lack of control over the texture of its sound and great variability between different samples of grass JH. Such combination is not likely to set a high ideological status for grass JH. It is rather more likely for it to remain within the everyday (mundane) sphere of use. So, musical instruments made of disposable and fragile organic materials are likely to be the most ancient (Wegst 2006). They were used as means of entertainment.

The wooden musical instruments present a much higher consistency in elastic properties from one piece to another of the same tree species, combined with quite long lasting sound – which is likely to rise the ideological status of the wooden instrument (Fletcher 2012). This increase in mechanical uniformity agrees with the ethnographic data that we provide in the main paper: the cult of "ancestor trees" accompanies the use of wooden musical instruments.

Hence, a wooden JH is likely to possess a much higher status than a grass JH. Even greater would be the importance of metallic JH, since metals provide a much greater uniformity than wood. This is exactly what we know from the informants in modern day indigenous JH traditions in Siberia. Thus, Khanty and Mansi consider bone or wooden *tumran* female and childish instrument, unworthy of "serious" use – in contrary to the metallic khomus that is worthy of shamanic use. What separates this opposition is exactly the solidity and permanence of metal as opposed to the fragility and finicky quality of wood and bone.

Wooden and bone JHs not only have a higher wear and tear, but they are also a lot more volatile than metals in their interaction with the environment. Their mechanical properties are easily affected by the smallest change in humidity and temperature. Water absorption and drying up immediately affect the sonic properties of wooden, bamboo and bone JHs. Additional source of variability in musical qualities between different specimen of the same musical instrument made of organic materials, is their inconsistency. Presence of knots and spiral grains in wood, and of nodes in bamboo greatly reduces the axial strength of these materials (Tsoumis 1991). Wood is also highly susceptible to deterioration from exposure to high moisture and heat, the action of which is cumulative and often irreversible (Esteves and Pereira 2009).

In this light, stability of musical features of the metallic JHs should be regarded the primary reason for their shamanic use. The transition from organic materials to metallic in the practice of making musical instruments must constitute one of the factors to support the transition from early animistic and totemic beliefs to shamanism as a peculiar form of Weltanschauung (N.Alekseyev 1992). If wooden JH suited the "ancestor tree" cult, and the bone JH continued to support the ancestor cult by relating "bone" to an ancestral figure as its "remnant," the permanence and uniformity of metallic JH allowed JH to break away from its role of the kin's talisman, and turned JH into a "universal" *obereg* – means of securing the magic protection of its owner disregarding the issue of his kin and ancestry.

Moreover, the mechanical properties of anisotropic versus isotropic materials seem to set the general direction for the development of musical textures and tonal organization. Not only anisotropy makes organic materials finicky in dependence of their acoustic properties on the pattern of orientation of fibers, grains, rings, nodes, culms and stems in relation to the vector of the action that excites the sounding body (e.g., strike), anisotropy is closely related to **hygroscopy** - the phenomenon of attracting and holding water molecules from the surrounding environment. Hygroscopy causes substantial changes in acoustic properties of the hygroscopic material. Moisture increases damping in orthotropic materials: thus, in wood it attenuates the resonance frequencies 5-10 kHz, reduces Young's modulus, and increases internal friction [loss $\boldsymbol{\eta}$] (Fukada 1951). Wood and bone are highly dependent on moisture, which is very high in vivo – therefore, for a prehistoric man to discover the musical properties of organic materials, these materials had to be isolated, dried and processed. Otherwise, all musically important acoustic parameters would have been greatly downplayed, producing dull, short and low-pitch sound, poorly differentiated between different specimens of musical instruments. It is only after the discovery of material-processing techniques that the sound could have been brightened and lengthened. Hence, variability of the mechanical properties of anisotropic and hygroscopic materials is likely to promote the invention and institutionalization of uniform standardized technologies of building musical instruments, called to overcome the inherent instability of musical properties. This, in turn, causes the standardization of musical textures and of tonal organization of musical sounds.

Each type of wood displays considerable differences in its acoustic properties from other types of wood (i.e. broad-leaf and needle-leaf trees). This corresponds well to the totem system of kinship as in the one identified in Altaic societies in the main paper. However, different specimen of the same wood can substantially vary in their qualities due to the difference mainly in moisture and also such aspects as density and age. This makes each specimen somewhat individualized and well suited to the practice of "individual" use, peculiar to the individual power of *obereg* for a given person. However, preparation of wood can be standardized, which would increase uniformity in its specimens – according to cultural convention. Such standardization also finds a sociological equivalent in setting norms of social interactions within larger social group, such as a tribe or a tribal confederation.

And once music users get used to the longer duration of musical tone and its specific musical attributes, they are likely to appreciate the stability of musical properties of metallic instruments. Comparing to wood, metal produces a greater number of partial vibrations of higher amplitude. For example, loss factor of a "resonance wood" becomes constant at 0.01 < 2 kHz, and rises to 0.03 at 10 kHz (Dunlop 1978). Once metallic musical instruments become attractive to music users, their attention is directed to the harmonic aspect of musical organization due to the inherent isotropy of metallic materials. At this point the development of musical texture and tonal organization falls "on the rails" of the evolution of metallurgy, which seems to follow a quite universal path across the world.

It would be most interesting to find out if the opposition between polyphonic disposition of musical instruments made of organic materials versus homophonic disposition of metallic musical instruments goes beyond JH and constitutes a universal trait across musical cultures. We know that the overall evolution of tonal organization that has taken place globally has occurred exactly from polyphonic to homophonic principles of arrangement of music. At the same time, clearly, ever since the Bronze Age, more and more musical instruments are made of metals. In the modern world absolute majority of musical instruments that can be used for solo music making are either entirely made of metal or use metallic parts for the generation of sound (e.g., piano, guitar, violin). It could be that the rise of homophonic musical thinking owes its rise to the long-term dominance of metallic musical instruments in practice of musicking.

Bibliography}

Agamennone, Maurizio. 1996. *Polifonie. Procedimenti, Tassonomie e Forme: Una Riflessione "a Più Voci."* Venice: Edizioni Il Cardo.

Aksyonov, Aleksey N. 1964. *Tuvan Folk Music [Тувинская Народная Музыка]*. Moscow: Muzyka [Музыка].

Alekseyev, B., and A. Miasoyedov. 1986. *Elementary Music Theory [Элементарная Теория Музыки]*. Moscow: Muzyka.

Alekseyev, Eduard Ye. 1976. *Problems in Genesis of Mode [Проблемы Формирования Лада]*. Moscow: Muzyka.

Alekseyev, Ivan Ye. 1988. *The Art of Playing Yakut Khomus [Искусство Игры На Якутском Хомусе]*. Yakutsk: Preprint.

Alekseyev, Nikolai A. 1992. *Traditional Religious Beliefs of Turkic-Speaking Peoples of Siberia [Традиционные Религиозные Верования Тюркоязычных Народов Сибири]*. Edited by I. S. Gurvich. Novosibirsk, Russia: Nauka.

Angster, Judit, and András Miklós. 2018. *Properties of the Sound of Flue Organ Pipes*. Edited by Rolf Bader. *Springer Handbook of Systematic Musicology*. Berlin: Springer-Verlag.

Anonymous. 2001. "Texture." Edited by Stanley Sadie and John Tyrrell. *The New Grove Dictionary of Music and Musicians*. London, UK: Macmillan Publishers. doi:10.1093/gmo/9781561592630.article.27758.

ASM International Handbook Committee. 1990. *ASM Handbook. Properties and Selection: Nonferrous Alloys and Special-Purpose Materials*. Vol. 2. Materials Park, OH: ASM International.

Audy, Jaromir, and Katarina Audy. 2008. "Analysis of Bell Materials: Tin Bronzes." *China Foundry* 5 (3): 199–204. doi:10.1118/1.4932367.

Awalluddin, Dinie, Mohd Azreen Mohd Ariffin, Mohd Hanim Osman, Mohd Warid Hussin, Mohamed A. Ismail, Han-Seung Lee, and Nor Hasanah Abdul Shukor Lim. 2017. "Mechanical Properties of Different Bamboo Species." *MATEC Web of Conferences* 138: 01024. doi:10.1051/matecconf/201713801024.

Bacon, Alice Louise. 2004. "A Technical Study of Alloy Compositions of 'Brass' Wind Musical Instruments (1651-1867) Utilizing Non-Destructive X-Ray Fluorescence." London: University of London. doi:9781303759659.

Balsamo, R. A., C. Vander Willigen, A. M. Bauer, and J. Farrant. 2006. "Drought Tolerance of Selected Eragrostis Species Correlates with Leaf Tensile Properties." *Handbook of Environmental Chemistry, Volume 5: Water Pollution* 97 (6): 985–91. doi:10.1093/aob/mcl068.

Beliayev, Viktor M. 1990. "Modal Systems in the Traditional Music of the USSR [Ладовые Системы в Музыке Народов СССР]." In *Viktor Mikhailovich Beliayev [Виктор Михайлович Беляев]*, edited by Irina Travkina, 223–377. Moscow: Sovetskii Kompozitor [Советский композитор].

Berry, Wallace. 1987. *Structural Functions in Music*. New York, USA: Dover Publications.

Bucur, Voichita. 1995. *The Acoustics of Wood*. Berlin: Springer.

———. 2016. *Handbook of Materials for String Musical Instruments*. Berlin: Springer. doi:10.1007/978-3-319-32080-9.

Callister, William D., and David G. Rethwisch. 2014. *Materials Science and Engineering: An Introduction.* 9th ed. John Wiley & Sons Inc.

Comberiati, Carmelo Peter. 1983. "On the Threshold of Homophony: Texture in Sixteenth-century Lute Music." *Journal of Musicological Research* 4 (3–4): 331–51. doi:10.1080/01411898308574535.

Cremer, L, M Heckl, and BAT Petersson. 2005. *Structure-Borne Sound.* 3rd ed. New York: Springer.

Dahlquist, Erik. 2013. *Technologies for Converting Biomass to Useful Energy*. Boca Raton, FL: CRC Press.

Demany, Laurent, and Catherine Semal. 2013. "Dividing Attention between Two Segregated Tone Streams." *Proceedings of Meetings on Acoustics* 19: 050078–050078. doi:10.1121/1.4798800.

Dmitriyev, Anatoly N. 1962. *Polyphony as the Factor of Form Creation: Theoretic Exploration Based on the Data of Russian Classical and Soviet Music [Полифония Как Фактор Формообразования: Теоретическое Исследование На Материале Русской Классической и Советской Музыки]*. Edited by Muzgiz [Музгиз]. Leningrad.

Dunlop, J. I. 1978. "Damping Loss in Wood at Mid Kilohertz Frequencies." *Wood Science and Technology* 12 (1). Springer-Verlag: 49–62. doi:10.1007/BF00390010.

Dunsby, Jonathan. 1989. "Considerations of Texture." *Music and Letters* 70 (1): 46–57. doi:10.1093/ml/70.1.46.

Eneh, C. T. M., M. K. H. Malo, J. P. Karjalainen, J. Liukkonen, J. Töyräs, and J. S. Jurvelin. 2016. "Effect of Porosity, Tissue Density, and Mechanical Properties on Radial Sound Speed in Human Cortical Bone." *Medical Physics* 43 (5): 2030–39. doi:10.1118/1.4942808.

Esteves, Bruno M, and Helena M Pereira. 2009. "Wood Modification by Heat Treatment: A Review." *Bioresources* 4 (1965): 370–404.

Fletcher, Neville H. 2012. "Materials in Musical Instruments." Vol. 40. Canberra: Australian National University. https://www.acoustics.asn.au/journal/2012/2012_40_2_Fletcher.pdf.

Fletcher, Neville H., and Thomas D. Rossing. 1998. *The Physics of Musical Instruments*. New York, London: Springer Science.

Frayonov, V. P. 1981. "Texture [Фактура]." Edited by Yurii Keldysh. *Encyclopedia of Music [Музыкальная Энциклопедия]*. Moscow, Russia: Soviet Encyclopedia [Советская энциклопедия]. http://www.music-dic.ru/html-music-enc/f/7832.html.

Fukada, Eiichi. 1951. "The Vibrational Properties of Wood. II." *Journal of the Physical Society of Japan* 6 (6). The Physical Society of Japan: 417–21. doi:10.1143/JPSJ.6.417.

Garcia, Manuel. 1847. *Mémoire Sur La Voix Humaine: Présenté à l'Académie Des Sciences En 1840*. Paris: E. Duverger.

Gough, Colin. 2014. "Musical Acoustics." In *Springer Handbook of Acoustics*, 567–701. New York, NY: Springer. doi:10.1007/978-1-4939-0755-7_15.

Green, Douglas, and Evan Jones. 2015. *The Principles and Practice of Tonal Counterpoint*. Abingdon, Oxfordshire: Routledge.

Gunji, Sumi. 1980. "An Acoustical Consideration of Xöömij." In *Musical Voices of Asia*, edited by Richard Emmert and Yuki Minegishi, 135–41. Tokyo: Heibonsha Publishers.

Halpern, Andrea R., Jeffrey S. Martin, and Tara D. Reed. 2008. "An ERP Study of Major-Minor Classification in Melodies." *Music Perception: An Interdisciplinary Journal* 25 (3): 181–91. doi:10.1525/mp.2008.25.3.181.

Hamayon, Roberte, and Mireille Helffer. 1973. "A Propos de 'Musique Populaire Mongole': Enregistrements de Lajos Vargyas." *Etudes Mongoles* 4: 145–80.

Haynes, W.M. 2014. *CRC Handbook of Chemistry and Physics*. https://www.taylorfrancis.com/books/9781482208689.

Hibbeler, Russell C. 2014. *Mechanics of Materials*. 9th ed. Upper Saddle River, NJ: Prentice Hall.

Holz, D. 1996. "Acoustically Important Properties of Xylophone-Bar Materials: Can Tropical Woods Be Replaced by European Species?" *Acta Acustica* 82 (6). S. Hirzel Verlag: 878–84.

Huron, David. 1989. "Voice Denumerability in Polyphonic Music of Homogeneous Timbres." *Music Perception: An Interdisciplinary Journal* 6 (4): 361-- 382.

———. 2001. "Tone and Voice: A Derivation of the Rules of Voice-Leading from Perceptual Principles." *Music Perception* 19 (1): 1–64. doi:10.1525/mp.2001.19.1.1.

Ikhtisamov, Khamza S. 1988. "On the Problem of Comparative Investigation of Two-Part Throat Singing and Instrumental Music of Turkic and Mongol Peoples [К Проблеме Сравнительного Изучения Двухголосного Гортанного Пения и Инструментальной Музыки у Тюркских и Монгольских Народов]." In *Folk Musical Instruments and Instrumental Music [Народные Музыкальные Инструменты и Инструментальная Музыка]*, edited by Yevgenii Gippius, 2:197–216. Moscow: Sovetskii Kompozitor [Советский композитор].

Janssen, Jules J.A. 1991. *Mechanical Properties of Bamboo*. Vol. 37. Dordrecht, the Netherlands: Springer.

Johnston, Richard, Michael John Simmons, and Frank Ford. 2005. *Acoustic Guitar: An Historical Look at the Composition, Construction, and Evolution of One of the World's Most Beloved Instruments*. Milwaukee, WI: Hal Leonard Books.

Kausel, Wilfried. 2018. *Vibrations and Waves*. Edited by Rolf Bader. *Springer Handbook of Systematic Musicology*. Berlin: Springer-Verlag.

Kholopova, Valentina. 1979. *Texture: An Essay [Фактура: Очерк]*. Moscow: Muzyka [Музыка].

———. 2002. *Theory of Music: Melos, Rhythm, Texture, Thematicism [Теория Музыки: Мелодика, Ритмика, Фактура, Тематизм]*. Saint Petersburg: Lan.

Kinsler, Lawrence E. 2004. *Fundamentals of Acoustics*. 4th ed. New York: John Wiley.

Kolltveit, Gjermund. 2004. "Jew's Harps in European Archaeology: A Brief Summary of a Research Project." *Journal of the International Jew's Harp Society* 1: 79–85.

Kubojima, Yoshitaka, Yoko Inokuchi, Youki Suzuki, and Mario Tonosaki. 2010. "Shear Modulus of Several Kinds of Japanese Bamboo Obtained by Flexural Vibration Test." *Journal of Wood Science* 56 (1): 64–70. doi:10.1007/s10086-009-1047-z.

Kudriavtsev, Aleksandr V., and Valentina A. Taranuschenko. 1956. *Theory of Music [Теория Музыки]*. Vol. 2. Moscow: State Edition of Cultural Education Literature [Госуд. Изд-во культурно-просветительской литературы].

Lakes, Roderic. 1993. "Materials with Structural Hierarchy." *Nature* 361 (6412): 511–15. doi:10.1038/361511a0.

Leaver, Amber M., and Andrea R. Halpern. 2004. "Effects of Training and Melodic Features on Mode Perception." *Music Perception: An Interdisciplinary Journal* 22 (1): 117–43. doi:10.1525/mp.2004.22.1.117.

Levin, Theodore Craig, and Valentina Suzukei. 2006. *Where Rivers and Mountains Sing: Sound, Music, and Nomadism in Tuva and Beyond*. Bloomington, IN: Indiana University Press.

Levy, Janet M. 1982. "Texture as a Sign in Classic and Early Romantic Music." *Journal of the American Musicological Society* 35 (3): 482–531. doi:10.1525/jams.1982.35.3.03a00040.

Li, Xiaobo. 2004. "Physical, Chemical, and Mechanical Properties of Bamboo and Its Utilization Potential for Fiberboard Manufacturing." Baton Rouge, Louisiana: Louisiana State University. doi=10.1.1.553.9762.

Mamcheva, Natalia A. 2005. "Nivkh Jew's Harps [Нивхские Варганы]." *Courier of Sakhalin Museum* 12: 271–84.

———. 2012. *Musical Instruments in Nivkh Traditional Culture*. Yuzhno-Sakhalinsk: GUP Sakhalinskaya Regional Press.

Mazel, Lev, and Viktor Tzukkerman. 1967. *Analysis of Muscal Works [Анализ Музыкальных Произведений]*. Moscow: Muzyka [Музыка].

Nadolski, M. 2017. "The Evaluation of Mechanical Properties of High-Tin Bronzes." *Archives of Foundry Engineering* 17 (1): 127–30. doi:10.1515/afe-2017-0023.

Natali, A.N., and E.A. Meroi. 1989. "A Review of the Biomechanical Properties of Bone as a Material." *Journal of Biomedical Engineering* 11 (4). Elsevier: 266–76. doi:10.1016/0141-5425(89)90058-7.

Nazaikinsky, Yevgenij V. 1972. *On Psychology of Human Musical Perception [О Психологии Музыкального Восприятия]*. Moscow: Muzyka.

———. 1982. *Logic of Musical Composition [Логика Музыкальной Композиции]*. Moskva: Muzyka.

Niklas, Karl J., and Hanns-Christof Spatz. 2012. *Plant Physics*. Chicago, IL: University of Chicago Press.

Nikolsky, Aleksey. 2015. "Evolution of Tonal Organization in Music Mirrors Symbolic Representation of Perceptual Reality. Part-1: Prehistoric." *Frontiers in Psychology* 6 (1405). doi:http://dx.doi.org/10.3389/fpsyg.2015.01405.

Noorden, Leon van. 1975. *Temporal Coherence in the Perception of Tone Sequences*. Vol. 3. Eindhoven, Holland: Institute for Perceptual Research.

Norton, M. P., and D. G. Karczub. 2003. *Fundamentals of Noise and Vibration Analysis for Engineers*. Cambridge: Cambridge University Press. doi:10.1017/CBO9781139163927.

O'reagain, Peter J. 1993. "Plant Structure and the Acceptability of Different Grasses to Sheep." Vol. 46.

Ostrovsky, Aron L. 1976. *The Course of Music Theory [Курс Теории Музыки]*. Leningrad: Muzyka [Музыка].

Palmer, Caroline, and Susan Holleran. 1994. "Harmonic, Melodic, and Frequency Height Influences in the Perception of Multivoiced Music." *Perception & Psychophysics* 56 (3): 301–12. doi:10.3758/BF03209764.

Pegg, Carole. 1992. "Mongolian Conceptualizations of Overtone Singing (Xöömii)." *British Journal of Ethnomusicology* 1 (1): 31–54. doi:10.1080/09681229208567199.

Piston, Walter. 1955. *Orchestration*. New York, NY: Norton.

Pyle, Robert W. 1998. "The Effect of Wall Materials on the Timbre of Brass Instruments." *The Journal of the Acoustical Society of America* 103 (5). Acoustical Society of America: 2834–2834. doi:10.1121/1.421388.

———. 2009. "Does a Brass-instrument's Timbre Depend on the Alloy from Which It Is Made?" *The Journal of the Acoustical Society of America* 125 (4). Acoustical Society of America: 2597–2597. doi:10.1121/1.4783883.

Ratner, Leonard G. 1980. *Classic Music: Expression, Form, and Style*. New York, NY: Schirmer Books.

Rostoker, William, Bennet Bronson, and James Dvorak. 1984. "The Cast-Iron Bells of China." *Technology and Culture* 25 (4): 750–67. doi:10.2307/3104621.

Rybakov, S. G. 1897. *Music and Songs of Uralic Muslims, Accompanied with the Essay on Their Lifestyle [Музыка и Песни Уральских Мусульман с Очерком Их Быта]*. Vol. 2. Sankt-Petersburg: The notes of the Imperial Academy of Science.

Schoeffler, Michael, Fabian-Robert Stöter, Harald Bayerlein, Bernd Edler, and Jürgen Herre. 2013. "An Experiment about Estimating the Number of Instruments in Polyphonic Music: A Comparison Between Internet and Laboratory Results." In *Proceedings of 14th International Society for Music Information Retrieval Conference*, edited by Jônatas Manzolli,

389–94. Curitiba, Brazil: ISMIR. http://www.audiolabs-erlangen.com/experiments/wice/.
Skrebkov, Sergei. 1973. *Artistic Principles of Musical Styles [Художественные Принципы Музыкальных Стилей]*. Moscow: Muzyka [Музыка].
Skrebkova-Filatova, Marina S. 1985. *Texture in Music [Фактура в Музыке]*. Moscow: Muzyka [Музыка].
Sliker, Alan, and Ying Yu. 1993. "Elastic Constants for Hardwoods Measured from Plate and Tension Tests." *Wood and Fiber Science* 25 (1). The Society: 8–22.
Spatz, Hanns-Christof, E. J. O'Leary, and Julian F. V. Vincent. 1996. "Young's Moduli and Shear Moduli in Cortical Bone." *Proceedings: Biological Sciences* 263. Royal Society: 287–94. doi:10.2307/50610.
Sposobin, Igor V., Sergei V. Yevseyev, and Iosif I. Dubovsky. 1935. *The Practical Course of Harmony [Практический Курс Гармонии]*. Vol. 2. Moscow: Muzgiz.
"Standard Practice for Measuring Ultrasonic Velocity in Materials." 2002. West Conshohocken, PA: Annual book of ASTM standards.
Stoter, Fabian-Robert, Michael Schoeffler, Bernd Edler, and Jurgen Herre. 2013. "Human Ability of Counting the Number of Instruments in Polyphonic Music." *The Journal of the Acoustical Society of America* 133 (5): 3366–3366.
Strafford, K.N., R. Newell, Katarina Audy, and Jaromir Audy. 1996. "Analysis of Bell Material from the Middle Ages to the Recent Time." *Endeavour* 20 (1). Elsevier Current Trends: 22–27. doi:10.1016/0160-9327(96)10003-X.
Suits, Juhan. 2007. "The Pärnu Type of Wedged Jew´s Harp: Quest for Historical Sources and Manufacturing Techniques." Telemark, Norway: Telemark University College.
Tiulin, Yuri N. 1937. *Teaching of Harmony [Учение о Гармонии]*. Moscow: Muzyka [Музыка].
Tongeren, Mark C. van. 2004. *Overtone Singing: Physics and Metaphysics of Harmonics in East and West*. Amsterdam: Fusica.
Tovey, Donald Francis. 1941. *Musical Textures. Alsop Lectures, University of Liverpool, 1938*. Oxford UK: Oxford University Press.
Trainor, Laurel J., Céline Marie, Ian C. Bruce, and Gavin M. Bidelman. 2014. "Explaining the High Voice Superiority Effect in Polyphonic Music: Evidence from Cortical Evoked Potentials and Peripheral Auditory Models." *Hearing Research* 308. Elsevier B.V: 60–70. doi:10.1016/j.heares.2013.07.014.
Tsoumis, George T. 1991. *Science and Technology of Wood: Structure, Properties, Utilization*. New York: Van Nostrand Reinhold.
Tylecote, Ronald F. 2002. *A History of Metallurgy*. London: Maney Pub., for the Institute of Materials.
Tzukkerman, Viktor. 1975. "Timbre and Texture in Orchestration by Rimsky-Korsakov [Тембр и Фактура в Оркестровке Римского-Корсакова]." In *Musical Theoretic Essays and Etudes [Музыкально-Теоретические Очерки и Этюды]*, 2:341–457. Moscow: Muzyka [Музыка].
Vargyas, Lajos. 1968. "Performing Styles in Mongolian Chant." *Journal of the International Folk Music Council* 20. International Council for Traditional Music: 70. doi:10.2307/836076.
Vos, Piet G., and Paul P. Verkaart. 1999. "Inference of Mode in Melodies." *Music Perception: An Interdisciplinary Journal* 17 (2): 223–39. doi:10.2307/40285892.
Waltham, Chris, and Shigeru Yoshikawa. 2018. *Construction of Wooden Musical Instruments*. Edited by Rolf Bader. *Springer Handbook of Systematic Musicology*. Berlin: Springer-Verlag. doi:10.1007/978-3-662-55004-5.
Wegst, Ulrike G.K. 2006. "Wood for Sound." *American Journal of Botany* 93 (10): 1439–48. doi:10.3732/ajb.93.10.1439.
———. 2008. "Bamboo and Wood in Musical Instruments." *Annual Review of Materials Research* 38 (1): 323–49. doi:10.1146/annurev.matsci.38.060407.132459.
Weiss, Stanley, Marylin C. Zimmerman, Robert D. Harten, F. G. Alberta, and Alain Meunier. 1998. "The Acoustic and Structural Properties of the Human Femur." *Journal of Biomechanical Engineering* 120 (1): 71–76.
Whitehouse, James W., and David B. Sharp. 2008. "A Psychoacoustical Investigation into the Effect of Wall Material on the Sound Produced by Lip-Reed Instruments." *The Journal of the Acoustical Society of America* 123 (5): 3120. doi:10.1121/1.2933035.
Zhang, J., R. J. Perez, and E. J. Lavernia. 1993. "Documentation of Damping Capacity of Metallic, Ceramic and Metal-Matrix Composite Materials." *Journal of Materials Science* 28 (9). Kluwer Academic Publishers: 2395–2404. doi:10.1007/BF01151671.
