# Supplementary material for: The Overlooked Tradition of “Personal Music” and Its Place in the Evolution of Music
Source: Front Psychol. 2020 Feb 18;10:3051. doi: 10.3389/fpsyg.2019.03051 (PMC7040865; doi:10.3389/fpsyg.2019.03051)
Supplement: Data Sheet 2 — List of audio examples. [file Data_Sheet_2.docx]

## List of audio examples for “The overlooked tradition of ‘personal music’”

1. Timbre-based vocal music: throat-singing song, “Seagull,” in archaic style, by Anna Ankhani, a 70-year-old Koryak woman from a remote reindeer-breeding settlement, Khailino, in Northern Kamchatka (reachable only by aerial transportation). Singing involves throat-rasping and double phonation that allows the singer to produce sounds an octave below her speaking voice range. <http://chirb.it/n5JNvk>
2. Modern style Koryak “Festive song” by Maria Appolon, a 48-year-old woman from a little town, Ossora, a seaport at Bering Sea. Maria is a legislator in the local district Council and a member of the folk ensemble, “Agya”, that often performs at international festivals, special events, and for tourists. Her singing exhibits traits of “frequency-based music”: no timbral effects, clear discrete pitches that are interconnected without gliding that is so typical for traditional singing in Kamchatka, hexatonic mode, consisting of 2 motifs (a descending trichord and an ascending tetrachord) which retain amazing precision in intonation, without noticeable fluctuations, and strict formulaic structure. <http://chirb.it/FK7A8A>
3. “Serenading” *khomus* (Yakut JH): a romantic duet in traditional style, performed by Erkin Alekseyev and Tokuiaana Nikolayeva. Music proceeds in a responsorial setting, but ends with simultaneous playing that reflects the union of feelings. <http://chirb.it/rH6bFD>
4. “Mary had a little lamb,” produced on khomus by Erkin Alekseyev. This music demonstrates inauthentic, Western-style treatment of JH as a “frequency-based” musical instrument whose purpose is to accurately reproduce the pitches of “tunes”. <http://chirb.it/HNcBsq>
5. “*Tuluktan doborum*” (“Friendly bullfinch”) – spontaneously improvised simultaneous singing and playing on khomus by Agrafena Ptitsyna from Megino-Kangalasskii district of Yakutia. The performer starts in the style of *degeren* (rhythm-oriented, metrically regular), but after 30 seconds switches to another style, *dieretii* (smooth, metrically free style), and thereafter keeps alternating between the two. Instrumental and vocal parts interfere with each other: khomus as though obstructs attempts of the voice to carry out a coherent song. <http://chirb.it/2D3pJh>
6. Dinimiaku’s children personal song, created and directed to Dinimiaku by her father, Tubiaku Kosterkin, from the settlement Ust’-Avam in Taimyr, in Nganasan. The song uses the timbral markers: contrasting “clean” high and “dirty” low registers connected together by sliding, and embellished with the “tremolo” effect. According to the performer, the song expresses tenderness and playful teasing. The lyrics question if the girl is upset at the old grouchy woman (Dobzhanskaya 2014, 159). <http://chirb.it/7htOzK>
7. Derkuptie’s children personal song, performed as if “coming from him” to the house guests, by his mother, Valentina Kosterkina, in Nganasan. The song is “infantilized”: raised in pitch and “whiny” – it emphasizes the descending sliding intonations, as though gently “complaining.” The lyrics imply that the infant-boy is overwhelmed by the visitors’ attention and wishes to be left alone (Dobzhanskaya 2014, 156). <http://chirb.it/zsxAEm>
8. Adult personal song of Ver’a Nenyang, performed by his daughter, Liubov’ Nenyang. Nenets masculine personal songs are characterized by praising one’s own strength, luck, and smartness (Nenyang 2006). Nenets adult personal songs usually oppose two contrasting vocal registers, joined by strong portamento, use of few “degrees” (3 in this example), and exaggerated intonations - the melodic intervals between the degrees are stretchable within the range of about 700 cents (Dobzhanskaya 2017). <http://chirb.it/dgenGN>
9. *Murun Yrya* (“nasal” song). Old age personal song of Maria Sleptsova-Kustui Maaia, of Yakut-Evenk ancestry, reproduced by her granddaughter, Marina Vasilyeva. Her grandmother used to sing this particular arrangement during knitting. The lyrics list many different garments made by Maria in the past and explain what they are good for (Dyakonova 2014). The song is characterized by nasalization and *kylysakh*, applied to the simple formula of 3 pitches (“degrees”). <http://chirb.it/4Fc24f>
10. Reproduction of *menerik yrya*, usually performed during the attacks of *meneriyi* by the anonymous old Yakut from Tattinskii ulus. He described his singing later as a reaction to seeing an evil spirit *abaasy* in the corner of his yurt and, causing him shriek in deep fear, trying to scare the spirit away. His yelling is interspersed with singing out the lines, supposedly pronounced by *abaasy*. Characteristic is the “dialogic” representation of at least two characters. <http://chirb.it/tIandL>
11. Another *menerik yrya*, imitated by Vissarion Gavrilyev from the Maar settlement in Niurbinskii ulus - according to his experience of frequently witnessing *meneriya* of his neighbor, an old woman. The lyrics are more comprehensible than in the example above and present an argument between the patient and the spirit *ichchi*. The song is characterized by the alternations of a recitative-like excited singing/talking and brief tremolo motifs in a free metric setting, interrupted (rather than accompanied) by spontaneous clapping. <http://chirb.it/mr28fk>
12. Genuine *tyyl yrya*, captured by Eduard Alekseyev from an overnight recording of Prokopii Sleptsov from the settlement Druzhina, Abyiskii ulus. Upon listening to this recording, Sleptsov remembered his dream of hunting a moose, but could not recognize the language of his singing (presumably, Yukaghir, a native tongue of his mother, that he later forgot). Singing is based on a brief descending gliding motif consisting of 2 pitches (degrees), possibly with a third complementary degree. <http://chirb.it/086zkG>
13. Reproduction of *tyyl yrya* of an old woman, a relative of the famous Yakut singer, Luka Turnin, who had overheard her singing on numerous occasions. The song is based on the repetitions of a brief formula of 6 tones, engaging 3 pitches (degrees) – most likely a personal motif of the old woman. The lyrics complain about disappointing the barn-master spirit, and promise to please the spirit with a gift. <http://chirb.it/cg0cvL>
14. Personal song of a Nenets old woman, Utchi, from the Kazym river region, covered with taiga (courtesy of Triinu Ojamaa). The melodic contour of this song’s formula is exceedingly simple – engaging only 3 degrees within the range of only 274 cents: the lowest (228-231 Hz), the middle (249-252 Hz) and the highest (c. 254-267 Hz) degrees. <http://chirb.it/0wM28B>
15. *Vyvko* – the Nenets buzzer used to imitate wind. In the past this had to do with the rituals of calling on rain, but now it is primarily a children toy, promptly made from a thread and a button. <http://chirb.it/mcGarg>
16. *Symysky* - the Khakass male maral call, made from a piece of birch bark. <http://chirb.it/8zt1tw>
17. *Temir-khomus* (Altaic JH) is imitating something that human voice cannot - the sound of the water stream, Tuva. <http://chirb.it/aa35p2>
18. The comparative demonstration of the principal khomus articulations: a) front versus b) back, c) high versus d) low vowels - performed by Ivan Alekseyev. Each of these 4 “poles” of JH articulations is characterized by salience of a particular register in generating a respective “JH formant”: 2.4-3.1 kHz for “front,” 0.3-1.6 kHz for “back,” 1.2-2.5 kHz for “high,” and 0.6-0.9 kHz for “low” vowels. The most similar are the “back” and “low” vowels, distinguished by greater intensity of the lowest 1^st^, 2^nd^, 3^rd^ and 4^th^ harmonics of the “back” vowel versus the “low” vowel which has much narrower bandwidth of its lowest formant. <http://chirb.it/Az51G7>
19. “Front” syllables alone. They require tension in throat, face, lips, jaw, cheeks and tongue – isolating the mouth chamber. Erkin Alekseyev describes his sensations while playing or hearing this articulation as though tasting an extremely sour apple. <http://chirb.it/HNwHpq>
20. “Back” syllables. They require relaxation in vocal apparatus. Erkin Alekseyev describes his sensations as “comfortable” to the extent of feeling “lazy.” <http://chirb.it/MCzDkN>
21. “High” syllables. They resemble “front” articulation, except that facial muscles remain relaxed. Erkin Alekseyev experiences this configuration as though “smiling” to oneself as in a situation when finding something funny. Emotionally, this state can be charged with joyfulness/playfulness (positive) or sarcasm (negative) – depending on how strained the larynx is. <http://chirb.it/n7z749>
22. “Low” syllables. They strongly activate the soft palate, configuring it into a “cupola.” Majority of khomus performers associate it with the sound of a big church-bell – and imagine something sublime and lofty while playing or listening to it. Like “high syllables,” sensing “low syllables” can appear “positive” or “negative” – depending on whether it is accompanied by the exertion of larynx and the discomfort resulting from this. <http://chirb.it/6Iy1z8>
23. Demonstration of a typical session of “playing for oneself” on Yakut khomus by Ivan Alekseyev. <http://chirb.it/bg32m9>
24. “*Hyttya-hyttya, syrdyk kүmmүt*” (Summer is coming), Yakut folk song, sung by Fedora Gogoleva, describes a bright sun looking out from the skies and sending its warmth to mark the beginning of summer. The song is based on a simple 3-degree formula with regular dance-like rhythm in the *degeren* style. However, timbrally, music stays in the *tangalai yryata* style – “palatal singing,” where tongue is abruptly pushed to the soft palate while taking a loud inspiration. <http://chirb.it/vpnhv1>
25. “*Hyttya-hyttya, syrdyk kүmmүt*” improvisation on khomus, Fedora Gogoleva. The same performer takes the melodic formula of the song above (Ex.24) and elaborates its motifs/articulations (*naigryshi*), arranged to form a proprietary JH composition that is intended to express joy and happiness. The JH version loses the traits of “palatal singing,” but keeps the *degeren* style rhythm. <http://chirb.it/dp391O>
26. “Personal song” of reindeer Urdy, performed “on behalf of him” by his owner, Valentina Kosterkina, in Nganasan. The lyrics describe reindeer’s exhaustion from work, complaints on a dog that likes to bite his legs and anticipation of a good rest (Dobzhanskaya 2014, 124). <http://chirb.it/M46DrK>
27. Nivkh JH PN by Vera Khein, as presented in her original improvisation which she named “The flatfish dance.” This piece, like many others that were recorded by Natalia Mamcheva from Vera Khein’s, is based on her personal motif consisting of two descending intervals: of a 3^rd^ and a 2^nd^ (e.g., E-C-Bb), where the latter is often marked by the shorter rhythm of the middle tone (C) and its multiple repetitions, marking the lowest tone (Mamcheva 2012, 297-300). <http://chirb.it/aIrCvG>
28. Nivkh JH PN by V.M. Persina, as presented in her improvisation (No.130 from Appendix 1, Mamcheva 2012, 301). It uses 2 motifs: of an ascending 2^nd^ (passing C-D-E or auxiliary C-D-C) and of an ascending auxiliary 3^rd^ (E-G-E), where C and E are marked as anchors, and the middle tones (D and E) are often given shorter rhythmic values. <http://chirb.it/2L65H3>
29. The Nivkh rhythmic formula “*Kan Vai*,” typical for various instruments and vocal music, in JH music is characterized by the onomatopoeic dog-like articulation of “khav-khav” (Mamcheva 2012, 119). This formula distinguishes the genre of a dog-racing music, used in festive competitions that are held by Nivkhi, Ulchi and Negidals. This genre is also used during the sacrifice of a dog in an annual bear festival (103). <http://chirb.it/q2xxbJ>
30. Grass JH, Nivkh *koka chnyr*, played by Zoya Angiun. The music features 5-part “scattered” polyphony, where all 5 registral components keep changing their pitch continuously and haphazardly throughout the music. Playing involves some poorly controlled random changes throughout the full spectrum of *koka chnyr*. <http://chirb.it/M6kNww>
31. Bamboo JH, frame-shaped, Ainu *mukkuri*, played by an anonymous performer. The music features 4-part “duplum” polyphony (2 continuous melodies in “alto” and “soprano,” dynamically equal), with ostinato figuration above the bass fundamental tone. Both melodies differ tonally: the upper melody features mostly semitones, whereas the lower is strictly anhemitonic. Playing involves continuous control of both melodies. <http://chirb.it/ktqFmJ>
32. Wooden JH, frame-shaped, Itelmen *varyga*, played by V. Ponomaryova. The music features 5-part “triplum” polyphony (3 continuous melodies in “tenor,” “alto” and “soprano,” the latter substantially quieter than the former two), with the pedal “chord” (cluster of overtones), sometimes diversified with “auxiliary” chords in the uppermost registral component. Playing involves continuous control of two lower melodies. The third melody (“soprano”) seems to be the “satellite” of the lower two. <http://chirb.it/gxhHrw>
33. Bone JH, frame-shaped, Mansi tumran, played by O. Tikhonova. The music features 4-part “triplum” polyphony (“tenor,” “alto” and “soprano,” the latter substantially quieter than the former two). The functionality of parts closely resembles the wooden JH - except that the uppermost pedal part is absent. <http://chirb.it/e6reg6>
34. Bronze JH, bow-shaped, Magyar doromb, reconstructed by Sergei Pyzhov from the archaeological find by Andrei Danich at Boyanovsky burial in Komi region, dated by X century AD (Danich 2016), played by Aksenty Beskrovny (used with his kind permission). The music features 3-part “homophony,” with the single melodic line in “tenor,” the pedal drone interval of 5^th^ in “bass,” and sostenuto “chords” in “alto.” <http://chirb.it/chLLBh>
35. Grass JH bass component. <http://chirb.it/0c4A60>
36. Grass JH tenor component. <http://chirb.it/3sK4DL>
37. Grass JH alto component. <http://chirb.it/mwAMgm>
38. Grass JH soprano component. <http://chirb.it/wKrcPB>
39. Grass JH descant component. <http://chirb.it/2O2Kma>
40. Bamboo JH bass component. <http://chirb.it/IHsCtI>
41. Bamboo JH tenor component. <http://chirb.it/3CKMmh>
42. Bamboo JH alto component. <http://chirb.it/espmII>
43. Bamboo JH soprano component. <http://chirb.it/gxzJNp>
44. Wooden JH bass component. <http://chirb.it/eeDfLC>
45. Wooden JH tenor component. <http://chirb.it/veEM5m>
46. Wooden JH alto component. <http://chirb.it/KMa5xq>
47. Wooden JH soprano component. <http://chirb.it/LmwOFy>
48. Wooden JH descant component. <http://chirb.it/r0rxDJ>
49. Bone JH bass component. <http://chirb.it/Ph47qs>
50. Bone JH tenor component. <http://chirb.it/bgq7P1>
51. Bone JH alto component. <http://chirb.it/nFNA9D>
52. Bone JH soprano component. <http://chirb.it/7tG0BN>
53. Bronze JH bass component. <http://chirb.it/r03G3q>
54. Bronze JH tenor component. <http://chirb.it/6Bzdyy>
55. Bronze JH alto component. <http://chirb.it/s3ByGq>
56. The *topahti* – a personal Nootka song of inherited origin, performed by Joe Titian. This topahti was given at the inter-tribal marriage between Nootka and Kwakiutl as a dowry, and permitted for performance only by its owner and her children (Halpern 1974). <http://chirb.it/NvahDq>

Bibliography}

Danich, Andrei V. 2016. “Research of the Bayanovskii Burial Ground [Исследования Баяновского Могильника].” In *Works of the Kama Archaeological-Ethnographic Expedition of the Perm’ State Pedagogical University [Труды Камской Археолого-Этнографической Экспедиции ПГГПУ]*, edited by Andrei M. Belavin, 11:36–43. Perm’, Russia: Perm’ State University.

Dobzhanskaya, Oksana. 2014. *The Singers and Songs of Avam Tundra: Musical Folklore of Nganasan [Певцы и Песни Авамской Тундры. Музыкальный Фольклор Нганасан]*. Norilsk, Russia: Apeks.

———. 2017. “Personal Songs of Tundra Nenets of Taimyr [Личные Песни Тундровых Ненцев Таймыра].” *Culture and Civilization* 7 (2B): 565–75.

Dyakonova, Varvara. 2014. “Data on Musical Culture of Sakha People of Verkhoyansk Region of Sakha Republic (Yakutia) [Материалы По Музыкальной Культуре Саха Верхоянского Района Республики Саха (Якутия)].” *The Courier of the Arctic State Institute of Art and Culture* 1 (6): 108–13.

Halpern, Ida. 1974. “Nootka Indian Music of the Pacific North West Coast.” New York: Smithsonian Folkways Recordings. FW04524 / FE 4524.

Mamcheva, Natalia A. 2012. *Musical Instruments in Nivkh Traditional Culture*. Yuzhno-Sakhalinsk: GUP Sakhalinskaya Regional Press.

Nenyang, Liubov’. 2006. *Songs over White Silence: Adult Personal and Children’s Songs-Niukubts of Taimyr Nenets in Russian and Nenets Languages [Песни Над Белым Безмолвием: Взрослые Личные и Детские Песни-Нюкубц Таймырских Ненцев На Русском и Ненецком Языках]*. Krasnoyarsk, Russia: KP Plus.
