# Supplementary figures and images for: The Overlooked Tradition of “Personal Music” and Its Place in the Evolution of Music
Source: Front Psychol. 2020 Feb 18;10:3051. doi: 10.3389/fpsyg.2019.03051 (PMC7040865; doi:10.3389/fpsyg.2019.03051)

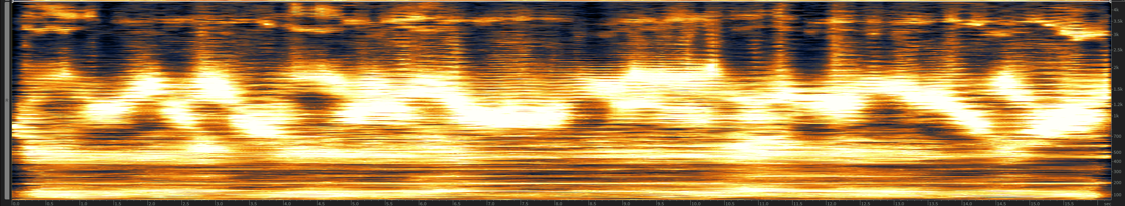

Supplement: Presentation 3 — Figures for Appendix. [file Presentation_3.zip › Figures for Appendix/Reduced Figures/Fig.6-varyga.png]

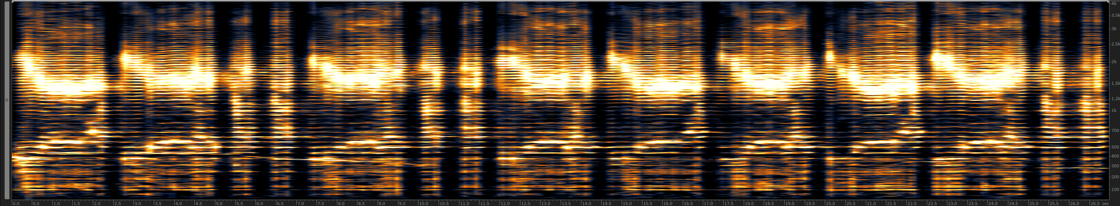

Supplement: Presentation 3 — Figures for Appendix. [file Presentation_3.zip › Figures for Appendix/Reduced Figures/Fig.7-jigatch.png]

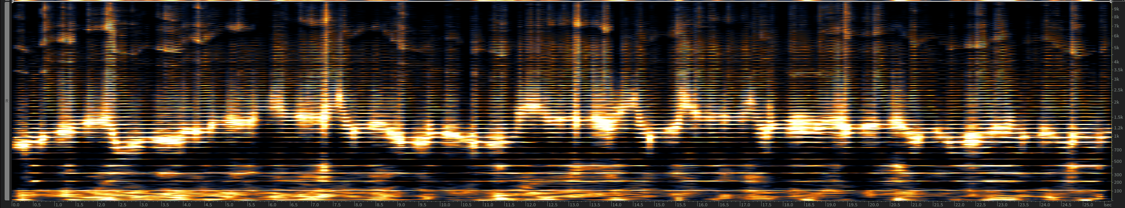

Supplement: Presentation 3 — Figures for Appendix. [file Presentation_3.zip › Figures for Appendix/Reduced Figures/Fig.16-doromb.png]

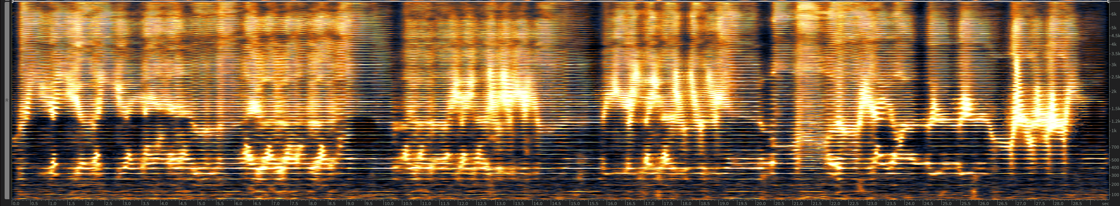

Supplement: Presentation 3 — Figures for Appendix. [file Presentation_3.zip › Figures for Appendix/Reduced Figures/Fig.15-vargan.png]

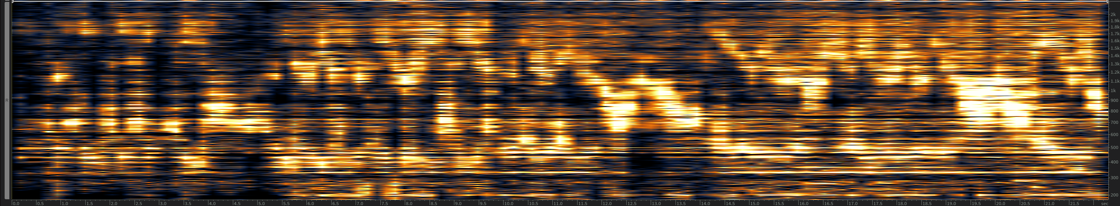

Supplement: Presentation 3 — Figures for Appendix. [file Presentation_3.zip › Figures for Appendix/Reduced Figures/Fig.9-tumran.png]

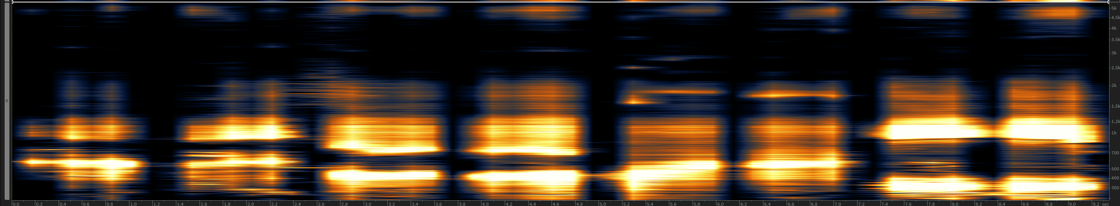

Supplement: Presentation 3 — Figures for Appendix. [file Presentation_3.zip › Figures for Appendix/Reduced Figures/Fig.10-khomus.png]

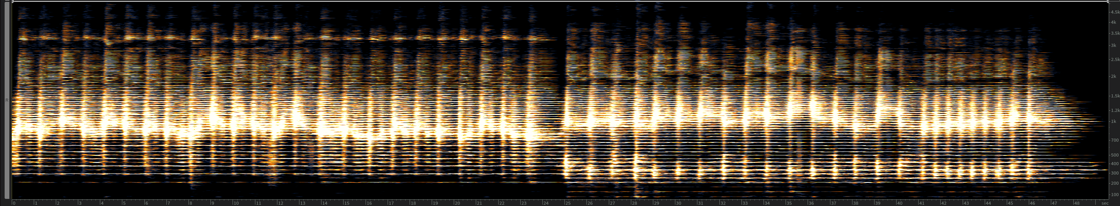

Supplement: Presentation 3 — Figures for Appendix. [file Presentation_3.zip › Figures for Appendix/Reduced Figures/Fig.22-khomus.png]

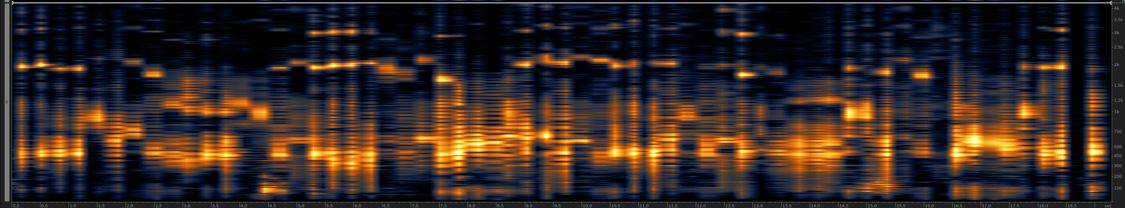

Supplement: Presentation 3 — Figures for Appendix. [file Presentation_3.zip › Figures for Appendix/Reduced Figures/Fig.1-koka.png]

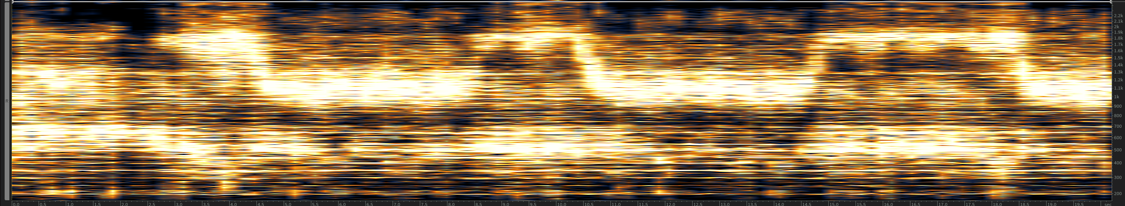

Supplement: Presentation 3 — Figures for Appendix. [file Presentation_3.zip › Figures for Appendix/Reduced Figures/Fig.8-tumran.png]

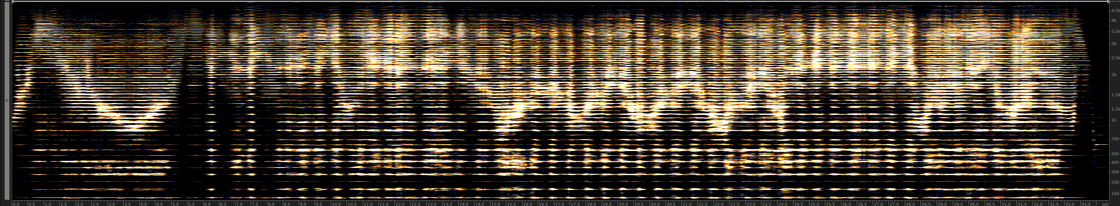

Supplement: Presentation 3 — Figures for Appendix. [file Presentation_3.zip › Figures for Appendix/Reduced Figures/Fig.23-vargan.png]

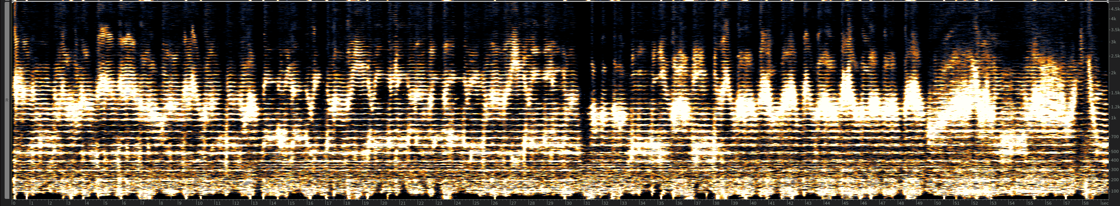

Supplement: Presentation 3 — Figures for Appendix. [file Presentation_3.zip › Figures for Appendix/Reduced Figures/Fig.19-kunkai.png]

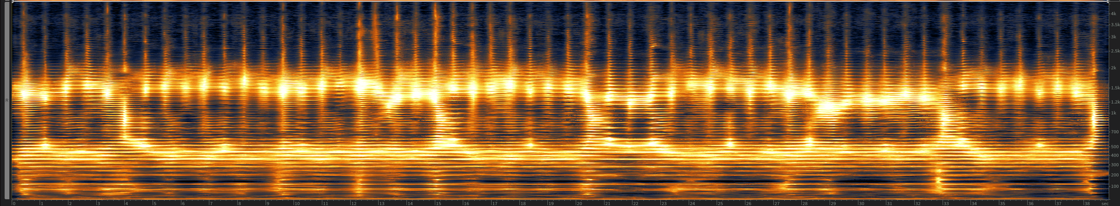

Supplement: Presentation 3 — Figures for Appendix. [file Presentation_3.zip › Figures for Appendix/Reduced Figures/Fig.14-kanga.png]

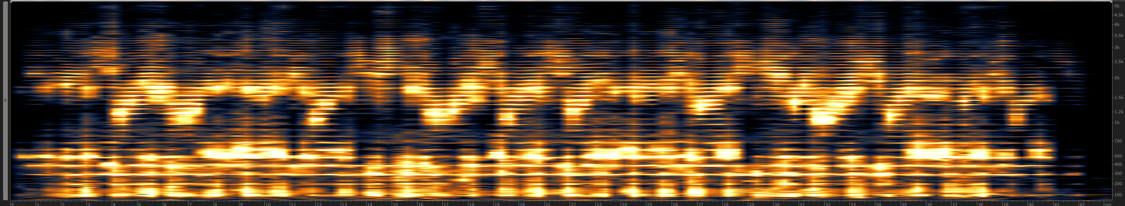

Supplement: Presentation 3 — Figures for Appendix. [file Presentation_3.zip › Figures for Appendix/Reduced Figures/Fig.2-mukkuri.png]

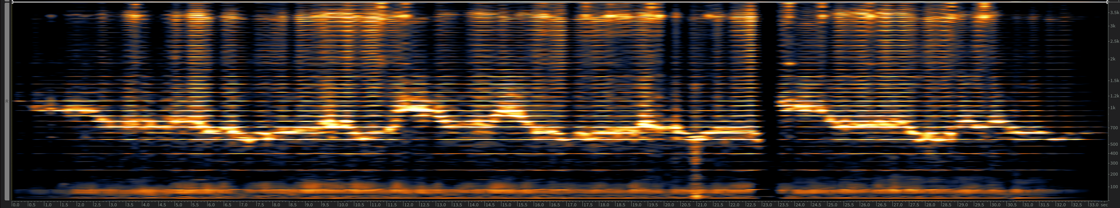

Supplement: Presentation 3 — Figures for Appendix. [file Presentation_3.zip › Figures for Appendix/Reduced Figures/Fig.3-cheler.png]

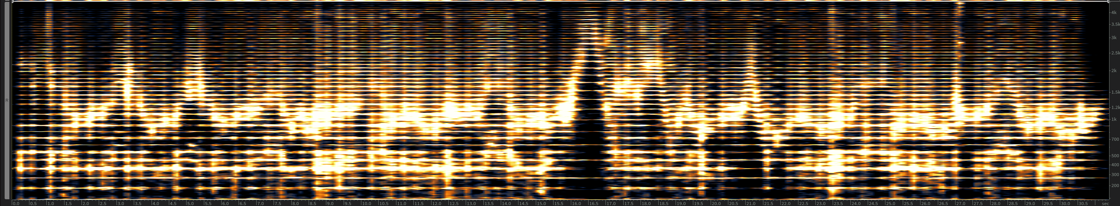

Supplement: Presentation 3 — Figures for Appendix. [file Presentation_3.zip › Figures for Appendix/Reduced Figures/Fig.17-murchunga.png]

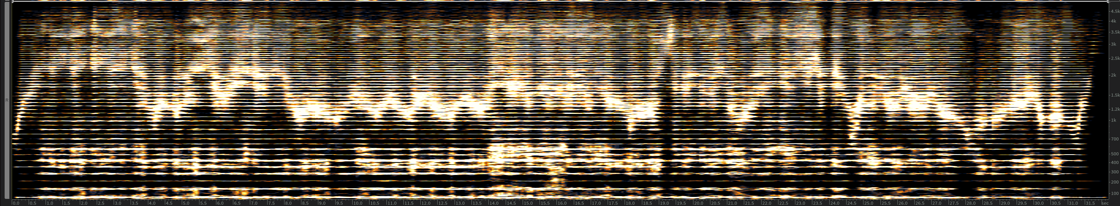

Supplement: Presentation 3 — Figures for Appendix. [file Presentation_3.zip › Figures for Appendix/Reduced Figures/Fig.21-vargan.png]

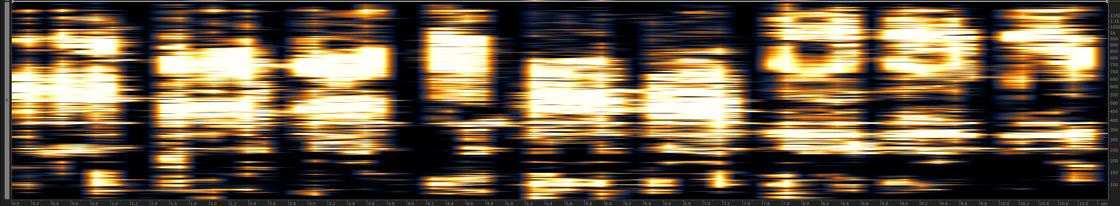

Supplement: Presentation 3 — Figures for Appendix. [file Presentation_3.zip › Figures for Appendix/Reduced Figures/Fig.12-kuluzun.png]

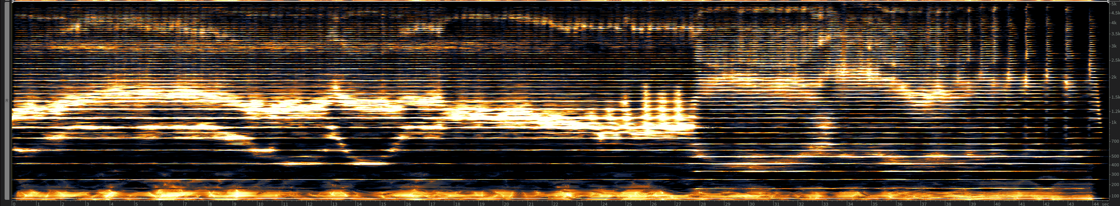

Supplement: Presentation 3 — Figures for Appendix. [file Presentation_3.zip › Figures for Appendix/Reduced Figures/Fig.5-mukkuri1-2.png]

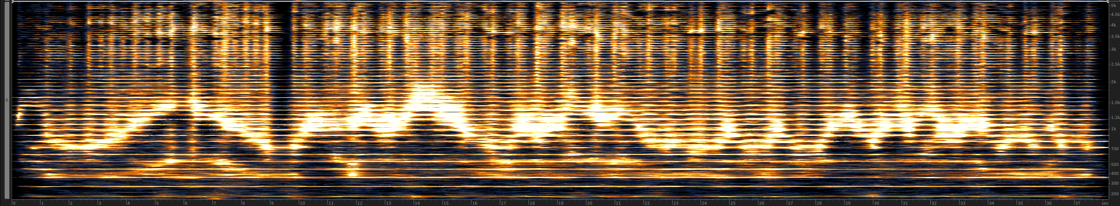

Supplement: Presentation 3 — Figures for Appendix. [file Presentation_3.zip › Figures for Appendix/Reduced Figures/Fig.18-aman.png]

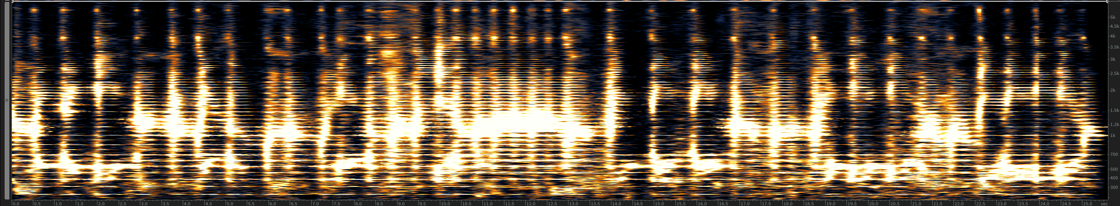

Supplement: Presentation 3 — Figures for Appendix. [file Presentation_3.zip › Figures for Appendix/Reduced Figures/Fig.20-zakanga.png]

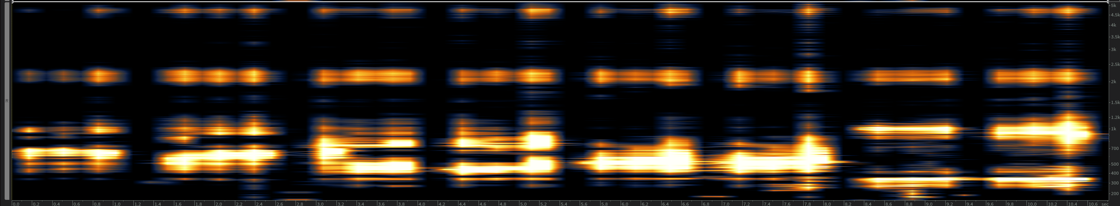

Supplement: Presentation 3 — Figures for Appendix. [file Presentation_3.zip › Figures for Appendix/Reduced Figures/Fig.11-khomus.png]

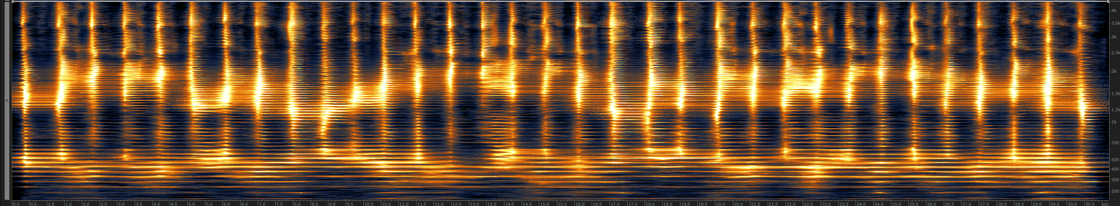

Supplement: Presentation 3 — Figures for Appendix. [file Presentation_3.zip › Figures for Appendix/Reduced Figures/Fig.13-kanga.png]

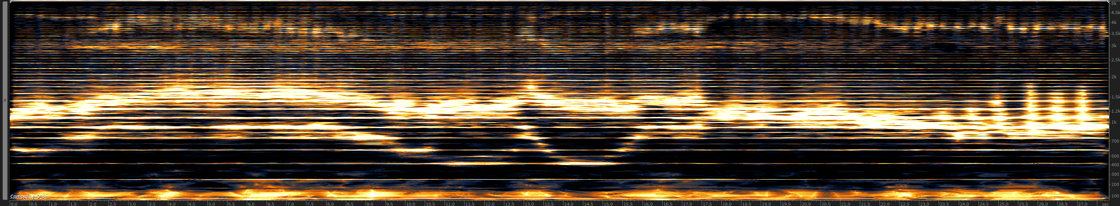

Supplement: Presentation 3 — Figures for Appendix. [file Presentation_3.zip › Figures for Appendix/Reduced Figures/Fig.4-mukkuri.png]
